# Genomic decoding of *Theobroma grandiflorum* (cupuassu) at chromosomal scale: evolutionary insights for horticultural innovation

**DOI:** 10.1093/gigascience/giae027

**Published:** 2024-06-05

**Authors:** Rafael Moysés Alves, Vinicius A C de Abreu, Rafaely Pantoja Oliveira, João Victor dos Anjos Almeida, Mauro de Medeiros de Oliveira, Saura R Silva, Alexandre R Paschoal, Sintia S de Almeida, Pedro A F de Souza, Jesus A Ferro, Vitor F O Miranda, Antonio Figueira, Douglas S Domingues, Alessandro M Varani

**Affiliations:** Embrapa Amazônia Oriental, 66095-903 Belém, PA, Brazil; Laboratório de Bioinformática e Computação de Alto Desempenho (LaBioCad), Faculdade de Computação (FACOMP), Universidade Federal do Pará, 66075-110 Belém, PA, Brazil; Departamento de Biotecnologia Agropecuária e Ambiental, Universidade Estadual Paulista (UNESP), Faculdade de Ciências Agrárias e Veterinárias, 14884-900 Jaboticabal, SP, Brazil; Departamento de Biotecnologia Agropecuária e Ambiental, Universidade Estadual Paulista (UNESP), Faculdade de Ciências Agrárias e Veterinárias, 14884-900 Jaboticabal, SP, Brazil; Departamento de Biotecnologia Agropecuária e Ambiental, Universidade Estadual Paulista (UNESP), Faculdade de Ciências Agrárias e Veterinárias, 14884-900 Jaboticabal, SP, Brazil; Departamento de Biologia, Universidade Estadual Paulista (UNESP), Faculdade de Ciências Agrárias e Veterinárias, 14884-900 Jaboticabal, SP, Brazil; Departamento de Ciência da Computação (DACOM), Grupo de e Bioinformática e Reconhecimento de Padrões (bioinfo-cp), Universidade Tecnológica Federal do Paraná (UTFPR), 80230-901 Cornélio Procópio, PR, Brazil; Artificial Intelligence and Informatics, The Rosalind Franklin Institute, OX110QX Didcot, UK; Laboratório de Bioinformática e Computação de Alto Desempenho (LaBioCad), Faculdade de Computação (FACOMP), Universidade Federal do Pará, 66075-110 Belém, PA, Brazil; Laboratório de Bioinformática e Computação de Alto Desempenho (LaBioCad), Faculdade de Computação (FACOMP), Universidade Federal do Pará, 66075-110 Belém, PA, Brazil; Departamento de Biotecnologia Agropecuária e Ambiental, Universidade Estadual Paulista (UNESP), Faculdade de Ciências Agrárias e Veterinárias, 14884-900 Jaboticabal, SP, Brazil; Departamento de Biologia, Universidade Estadual Paulista (UNESP), Faculdade de Ciências Agrárias e Veterinárias, 14884-900 Jaboticabal, SP, Brazil; Centro de Energia Nuclear na Agricultura (CENA), Universidade de São Paulo, 13416-000 Piracicaba, SP, Brazil; Departamento de Genética, Universidade de São Paulo (USP), Escola Superior de Agricultura Luiz de Queiroz (ESALQ), 13418-900 Piracicaba, SP, Brazil; Departamento de Biotecnologia Agropecuária e Ambiental, Universidade Estadual Paulista (UNESP), Faculdade de Ciências Agrárias e Veterinárias, 14884-900 Jaboticabal, SP, Brazil

**Keywords:** Amazon basin, bioeconomy, cupuassu, fruit pulp and seed development, genome evolution, gene loss and retention, positive selection, plant secondary metabolites

## Abstract

**Background:**

*Theobroma grandiflorum* (Malvaceae), known as cupuassu, is a tree indigenous to the Amazon basin, valued for its large fruits and seed pulp, contributing notably to the Amazonian bioeconomy. The seed pulp is utilized in desserts and beverages, and its seed butter is used in cosmetics. Here, we present the sequenced telomere-to-telomere genome of cupuassu, disclosing its genomic structure, evolutionary features, and phylogenetic relationships within the Malvaceae family.

**Findings:**

The cupuassu genome spans 423 Mb, encodes 31,381 genes distributed in 10 chromosomes, and exhibits approximately 65% gene synteny with the *Theobroma cacao* genome, reflecting a conserved evolutionary history, albeit punctuated with unique genomic variations. The main changes are pronounced by bursts of long-terminal repeat retrotransposons at postspecies divergence, retrocopied and singleton genes, and gene families displaying distinctive patterns of expansion and contraction. Furthermore, positively selected genes are evident, particularly among retained and dispersed tandem and proximal duplicated genes associated with general fruit and seed traits and defense mechanisms, supporting the hypothesis of potential episodes of subfunctionalization and neofunctionalization following duplication, as well as impact from distinct domestication process. These genomic variations may underpin the differences observed in fruit and seed morphology, ripening, and disease resistance between cupuassu and the other Malvaceae species.

**Conclusions:**

The cupuassu genome offers a foundational resource for both breeding improvement and conservation biology, yielding insights into the evolution and diversity within the genus *Theobroma*.

**Key points:**

- The chromosome scale of *Theobroma grandiflorum* was generated and revealed a 65% synteny with *Theobroma cacao*.
- Long terminal repeat retrotransposon expansion was a pivotal factor for postdivergence genomic evolution between *Theobroma* species.
- Comparative genomics provided evolutionary insights of the genes associated with key agronomic traits.
- Positive selection pressure of retained duplicated genes was implicated in adaptive functions and fruit seed trait diversity.
- The cupuassu genome is a genetic resource for breeding and to boost Brazilian Amazonian bioeconomy.

## Data Description

**Figure 1:**
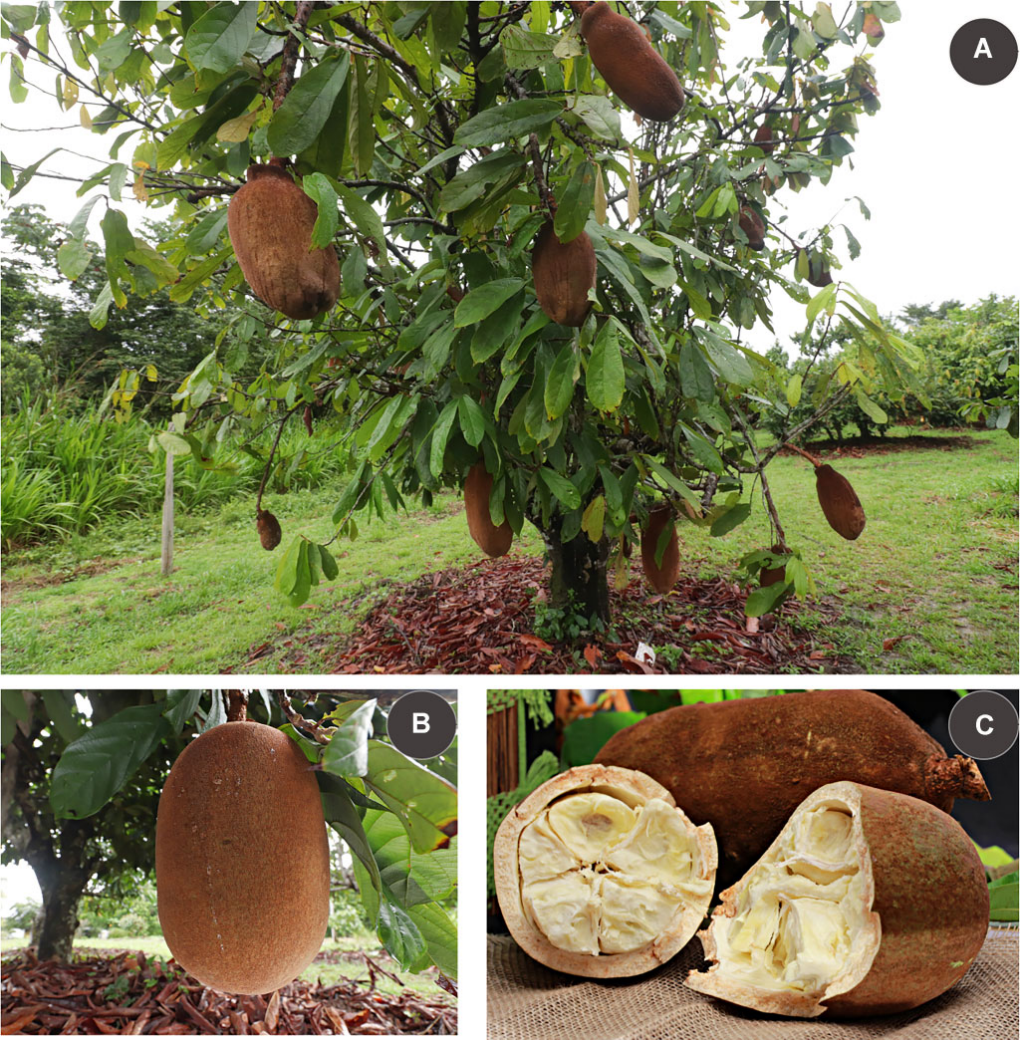
(A) *T. grandiflorum* tree displaying fruits. (B) Detailed view of a cupuassu fruit. (C) Cupuassu fruit opened to reveal the internal pulp. Image credits: Ronaldo Rosas.

Cupuassu (Fig. 1), a fruit-bearing tree closely related to cacao and native to the Amazon, is highly valued for its flavorful seed-pulp and fatty seeds, extensively used in the food and cosmetics industries. We conducted a comprehensive sequencing of the cupuassu genome employing technologies of PacBio HiFi, genome-wide chromatin interaction analysis via Hi-C alongside Illumina sequencing. We generated a total of 1.4 million HiFi reads and 445 million Hi-C paired reads, which were assembled into a chromosome-scale assembly. Furthermore, to assist the gene prediction and annotation, we generated transcriptomic data from young and fresh leaf tissues using PacBio HiFi Iso-seq and Illumina RNA-seq, yielding 4.5 million and 46 million paired reads, respectively. Approximately 25% of the cupuassu genome consists of gene-coding regions, encompassing a total of 31,381 genes. Comparative genomics analyses revealed that the cupuassu genome shares a high gene synteny and nucleotide similarity with cacao, but it also exhibits distinctive features. Notably, repetitive DNA elements, which account for at least 54% of the genome, have significantly influenced its genomic structure. Furthermore, specific genes responsible for its fruit and seed characteristics, as well as disease resistance, were identified. Overall, this work generated data that not only deepen our knowledge of cupuassu genetics but also illuminate broader aspects of plant evolution and diversity in the Amazon. It lays the groundwork for advanced breeding programs and promises to contribute significantly to the Amazonian bioeconomy.

## Context

The genus *Theobroma* L. (Malvaceae) originated in the Neotropical Region, with the Amazon basin as its main ecosystem. Among the 22 *Theobroma* species [1, 2], 2 species, *Theobroma cacao* L. (cacao) and *Theobroma grandiflorum* (Willd. ex Spreng.) K.Schum. (cupuassu), are of significant economic importance. Both of them are diploid (2*n* = 2× = 20), presenting an average genome size around 450 Mb [3]. These species display distinct fruit and seed morphologies, which are likely the most valued parts by humans and other dispersers [4]. Cacao seeds are the main component for the chocolate and confectionery industries. In contrast, cupuassu seed pulp is used in desserts and beverages. Additionally, cupuassu seeds can be processed to create a butter highly prized in the cosmetic industry and “cupulate,” a product akin to chocolate [5].

Cupuassu, domesticated from *Theobroma subincanum* Mart. by Amazon indigenous populations approximately 5,000 to 8,000 years ago, has spread geographically mainly in the past 2 centuries [6]. In Brazil, cupuassu is especially important for small-scale farmers in agroforest systems in Pará, Amazonas, and Bahia, the leading states in its production [7]. In 2022, Brazilian cupuassu production reached about 28,800 tons of fresh seeds from 8,900 hectares, averaging 3.2 tonnes per hectare (State Secretariat for Agricultural Development, Agricultural Indicators, Belém, PA, Brazil, 2022).

Both cacao and cupuassu face substantial threats from various fungal and viral pathogens. Specifically, the witches’ broom disease (WBD) and frosty pod (FP) pose major challenges in the Americas. Both diseases are caused by 2 basidiomycete species, *Moniliophthora perniciosa* (Stahel) Aime & Phillips-Mora and *Moniliophthora roreri* (Cif.) H.C.Evans, Stalpers, Samson & Benny, respectively. These diseases significantly reduce pod yield and the overall health of infected plants, resulting in substantial economic losses [8]. While breeding programs have identified resistant cacao and cupuassu genotypes [7, 8], managing WBD and FP remains challenging [9, 10], impacting local producers and family farmer systems.

Numerous sequencing initiatives have been undertaken for cacao to provide insights into the genome biology and plant–pathogen interactions, as well as assist breeding over the past 15 years [11–16]. To date, 37 chromosome-scale *T. cacao* genomes are publicly accessible, encompassing a range of genotypes from widely cultivated to wild-collected accessions. Additionally, the genome sequence of *Herrania umbratica* R.E.Schult, a sister genus to *Theobroma* (both representatives of the Theobromateae tribe), known as “monkey cacao,” which exhibits unique morphology [17], is also available.

In parallel, recent investigations have delved into the genomic architecture of *T. grandiflorum*, ranging from developing the first genetic map [18], the chloroplast and mitochondrial genomes [19, 20], and in comparative transcriptomics [10, 21]. These latter studies shed light on the interaction between cupuassu and *M. perniciosa*, setting the groundwork for breeding programs and transgenic approaches. However, limited genomic data for *T. grandiflorum* persist, leaving gaps in understanding its genome evolution, biology, and potential comparison with *T. cacao*, a key crop in the genus.

In this study, we present a detailed analysis of the *T. grandiflorum* genome, assembling a high-quality telomere-to-telomere (T2T) chromosome-scale genome. Our comparative genomic approach reveals important genomic features, distinguishing it from related species like *T. cacao* and *H. umbratica*. These insights provide critical targets for breeding and are of significant importance for evolutionary biology, biotechnology, conservation, and horticulture research.

## Methods

### Plant sampling, DNA and RNA extraction, and sequencing

Leaf samples of the cupuassu (NCBI:txid108881) clone 1074, susceptible to WBD [18], were collected at the “Embrapa Amazônia Oriental” collection in Belém, PA, Brazil (1.4359°S, 48.4495°W), and cataloged at the Herbarium JABU [22], Universidade Estadual Paulista, Jaboticabal campus (Voucher JABU1370). The samples underwent a 24-hour dark incubation, were flash-frozen in liquid nitrogen, and were transported to the Arizona Genomics Institute (Tucson, USA) for analysis. High molecular weight (HMW) DNA were extracted using a modified CTAB protocol [23], as well as assessed for integrity and concentration via Qubit dsDNA High-Sensitivity Assay (Thermo Fisher Scientific) and NanoDrop ND-1000 (NanoDrop Technologies). DNA quality and size were confirmed with Femto Pulse and pulse-field gel electrophoresis (Femto Pulse System; Agilent Technologies). The DNA was sheared to 10–30 Kb using a Covaris g-TUBE (Covaris, Inc.), purified, and sequenced on a PacBio Sequel IIe platform (PacBio). GenomeScope 2.0 [24] and KMC v3.2.1 [25] were employed to evaluate the ploidy and size of genome.

Total RNA was extracted and purified using the PureLink Plant RNA Reagent (Thermo Fisher Scientific) and Takara NucleoSpin RNA Clean-up (Takara Bio). RNA integrity was confirmed by a 2100 Bioanalyzer (Agilent Technologies), and only samples with an RNA integrity number above 7 proceeded to sequencing. Iso-seq library preparation and sequencing were performed on a PacBio Sequel IIe, while Illumina sequencing (2 × 100 bp) was conducted on a HiSeq 2000 platform (Illumina) at NGS Soluções Genômicas, Brazil.

For HiC library preparation and sequencing, samples were processed at Novogene Bioinformatics Technology using the Proximo Hi-C Kit. The quality control was conducted using Phase Genomics' hic_qc scripts [26].

### Genome assembly and quality evaluation

PacBio HiFi reads were assembled employing Hifiasm (RRID:SCR_021069) v0.19.3-r572 [27] with default parameters. Contaminants were removed using kraken2 [28] and “extract_kraken_reads.py” v1.2 [29], with the PlusPFP index database (version 5/17/2021) [30]. The primary assembly was indexed with BWA (RRID:SCR_010910) v0.7.17-r1188 [31], and *Dpn*II restriction sites were created using the Juicer pipeline v1.6 [32]. Genome scaffolding and chromosomal reconstruction were achieved using 3D-DNA v180419 [33] and manually corrected with Juicebox Assembly Tools v3.1.4 [32]. The final chromosome-level assembly was refined using the “run-ASM-pipeline-post-review.sh” script from 3D-DNA and “close_scaffold_gaps.sh” from the MaSuRCA assembler package (RRID:SCR_010691) v4.1.0 [34]. The adopted chromosome numbering was based on that used for *T. cacao*.

For the *H. umbratica* accession Fairchild (BioProject: PRJNA383741), we reassembled the genome using the MaSuRCA hybrid approach with PacBio CLR and Illumina reads. Genome scaffolding for this genotype employed the Arima Genomics’ mapping pipeline [35] and YaHS v1.1 [36].

The *T. grandiflorum* assembled genome quality and completeness were validated using Merqury (RRID:SCR_022964) v1.3 [37], Inspector v1.2 [38], LTR Assembly Index (LAI) [39], and BUSCO v5.4.5 against the embryophyta_odb10 database [40, 41].

### Transcriptome and Iso-seq assembly

Iso-seq transcripts fasta file was generated using the SMRT Link 12.0 (PacBio) with default parameters. *De novo* assembly of RNA-seq short reads and HiFi reads was performed using the Trinity pipeline v2.14.0 [42]. For genome-guided transcriptome assembly, the short reads and HiFi reads were separately aligned to the chromosome-scale genome using histat2 v2.2.1 [43] and minimap2 v2.24-r1122 [44], respectively. The aligned BAM files from both read types were then merged using StringTie2 v2.2.1 [45] to produce a GTF file, which was utilized in the genome annotation process.

We employed the PASA (RRID:SCR_014656) v2.5.3 pipeline [46], integrating Iso-seq fasta, *de novo*, and genome-guided assemblies with StringTie2, along with TransDecoder (RRID:SCR_017647) v5.7.0 [47] to create a comprehensive transcriptome database and to annotate transcript structures (Supplementary Information 1). This methodology was applied to both *T. cacao* v2 (Belizian Criollo B97-61/B2 cultivar) [12] and *H. umbratica* (Fairchild) transcriptomes, using public short reads from the GenBank Sequence Read Archive (Supplementary Table S1).

The completeness of the assembled transcriptome was assessed using BUSCO v5.4.5 against the embryophyta_odb10 database in transcriptome mode.

### Genome annotation and comparative analyses

The genome annotation was carried out in 2 phases, following best practices in plant genome annotation [48]. Detailed methodologies are delineated in Supplementary Information 1.

In the first phase, transposable elements (TEs) and other repetitive sequences were identified and annotated utilizing an in-house pipeline [49] based on the Extensive *de novo* TE Annotator (EDTA) v2.0.1 [50]. Subsequently, the soft-masked genome sequence was further annotated through the integration of gene predictors and combination tools and functional annotation software, including BRAKER (RRID:SCR_018964) v3.0.4 [51], EVidence Modeler v2.1.0 [52], PASA, and BLAST2GO Basic v6.0 [53]. Identification of telomeric and centromeric repeats was accomplished using the quarTeT tool (RRID:SCR_025258) (commit: e1a2f72) [54] and the Centromics pipeline [55, 56], respectively.

The genome map was created using shinyCircos-V2.0 [57]. Whole-genome duplication (WGD) and positive selection analyses followed established methods [58]. In summary, WGD-derived gene pairs were identified using the *DupGen_finder* pipeline [59]. For each duplicate pair of duplicated gene, the protein sequences were aligned using MAFFT (RRID:SCR_011811) v7.490 [60] with the L-INS-i option. These protein alignments were then converted into a codon alignment using PAL2NAL v14 [61]. The nonsynonymous (Ka) and synonymous (Ks) substitution rates were calculated using the γ-MYN method [62], as implemented in KaKs_Calculator 2.0 [63] by applying the Tamura–Nei model [64]. Ks values exceeding 5.0 were omitted from subsequent analyses to avoid complications arising from saturated substitutions at synonymous sites. Macrosynteny and microsynteny were analyzed using MCScanX (commit: b1ca533) [65], SynVisio [66], and the Python version of MCscan [67, 68], with synteny percentages computed using custom Python scripts based on MCscan outputs.

Chromosome plots were generated with the jcvi miscellaneous plotting tool [69] and the MG2C tool v2.1 [70]. TE distribution relative to genes was determined using TE_Density (commit: 09b3e90) [71]. The TE distribution plot was generated with RAWgraphs v2.0 [72]. Orthologous gene clusters (gene families) were identified using OrthoFinder2 algorithm v2.5.5 [73] and OrthoVenn3 [74] with diamond v2.0.14 in super-sensitive mode [75]. Gene family evolution was analyzed using CAFE 5 v1.1 [76]. For comparative purposes and to root the phylogenetic tree, the cotton D genome (*Gossypium raimondii*) v. 2.1 [77] and *Arabidopsis thaliana* (version Araport11) [78] were employed as outgroup. The divergence time between the species selected were estimated using the TimeTree5 resource [79].

Gene Ontology (GO) enrichment analyses were performed with GOATOOLS (commit: eff7681) [80], considering only results with a *P* value below 0.05 after false discovery rate correction with a Benjamini–Hochberg significance test. Targeted comparative analyses focused on genes and functions previously related to seed traits and fruit characteristics, such as aroma, quality, maturation, and flavor, incorporating components like purine alkaloids, flavonoids, terpenoids, and fatty acids [11, 81]. This was supplemented by literature and GO searches through the QuickGO platform [82].

### Data validation and quality control

#### High-resolution chromosome-level genome assembly of T. grandiflorum

**Figure 2:**
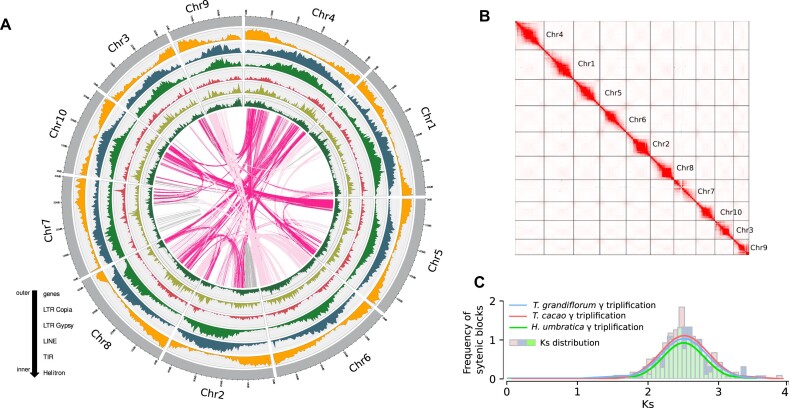
(A) Depiction of the genomic landscape of *T. grandiflorum*, illustrating gene and TE density across the 10 chromosomes. (B) High-throughput chromosome conformation capture (Hi-C) contact map revealing the assembled chromosomes of *T. grandiflorum*. (C) Whole-genome duplication analyses indicating the shared whole-genome triplication among *T. grandiflorum, T. cacao*, and *H. umbratica* and confirming the absence of additional WGD events in these species.

The chromosome-level genome assembly of *T. grandiflorum* was achieved by integrating HiFi sequencing reads with Hi-C data. The total size of the assembled genome was 423 Mb, consisting of 10 chromosome-level scaffolds with lengths ranging from 28 to 53 Mb and heterozygosity rate of 0.61% (Fig. 2A, B, Table 1, and Supplementary Table S2). This assembly represents approximately 94% of the haploid genome size estimated by flow cytometry [3]. The average GC content of the cupuassu genome is 34.01%, comparable to *H. umbratica* (33.76%) and to *T. cacao* (32.14%). Moreover, the *T. grandiflorum* assembly is almost gap free, presenting only 3 gap regions located close to the telomeric repeats of chromosomes 6 and 8, and in the centromeric region of the chromosome 5, associated with a long terminal repeat (LTR)/large retrotransposon derivative (LARD) element (Supplementary Table S3). Telomeric repeats were identified at both ends of 7 chromosomes, whereas a single telomeric repeat was observed at one end of the remaining 3 chromosomes (Supplementary Table S4). Centromeric repeats, identified on all chromosomes (Supplementary Table S5), largely align with heterochromatic bands previously established through cytogenetic studies [83]. The chromosome-level assembly displays an elevated BUSCO score (98.4%) and LAI (15.6), both compatible to a reference quality genome. Furthermore, the assembly evaluation using Merqury and Inspector shows a very high genome completeness, mapping rate, and depth, as well as very low error rates, revealing a high accuracy of the assembled *T. grandiflorum* genome.

**Table 1:** Statistics of *T. grandiflorum* genome sequencing and assembly

Genome Sequencing Statistics	Value
HiC sequencing	
Number of HiC reads	445,532,022 (2 × 150 bp)
Average Phred value	Q38
GC content of the HiC reads	38%
Same strand high-quality read pairs*	24.33% (expected >1.5%)
Informative read pairs**	45.87% (expected >5%)
HiFi sequencing	
Number of HiFi reads	1,983,315 (30 Gbp)
N50 HiFi reads	15,327 bp
Average Phred value	Q60
GC content of the HiFi reads (%)	35.29
*k*-mer heterozygosity rate	0.61%
Genome assembly statistics	
Genome length	423,916,809 bp
GC content of the genome (%)	34.01
Assembly gaps	3
Chromosomes	10
Predicted centromeres	10 (one for each chromosome)
Predicted telomeres	17
BUSCO analysis	embryophyta_odb10 (1,614)
Complete	98.4% (1,588)
Complete and single copy	97.5% (1,574)
Complete and duplicated	0.9% (14)
Fragmented	0.9% (15)
Missing	0.7% (11)
LAI***	15.6
Merqury analysis	
Estimate base-level quality value (QV)	67.907
*k*-mer completeness	88.4602
*k*-mer error rate	0.0000161919%
Inspector analysis	
Mapping rate	95.99%
Depth	68.1158
QV	47.8364
Error rate (*E*, from QV = −10log_10_*E*)	0.00165%

* High-quality read pairs have minimum mapping quality ≥20, have maximum edit distance ⇐ 5, and are not duplicates.

** Informative read pairs are read pairs that have MAPQ >0, are not PCR duplicates, and map to different contigs or >10 kb apart.

*** To enhance long terminal repeat (LTR) identification in *T. grandiflorum*, the maximum distance between LTRs was set to 20,000 base pairs, which is expected to increase the size of intact elements. Consequently, this adjustment is anticipated to result in a marginally higher LTR Assembly Index (LAI) value [34]. For further details, please refer to Supplementary Information 1.

### Structural annotation and gene arrangement

A total of 31,381 protein-coding genes, corresponding with up to 25% of the entire genome length, were identified (Table 2 and Supplementary Table S6). The structural gene annotation achieved a BUSCO completeness of 99.8%, indicating a high-quality annotation. Through RNA-seq and Iso-seq read mapping, 46,625 complete coding sequences (CDSs) were determined, confirming the functional isoforms in the gene models. The average gene length was 3,374 bp and CDS length 1,331 bp with 6 exons, values similar to *T. cacao* [11]. Furthermore, their distribution is evenly spread across the 10 chromosomes.

**Table 2:** *T. grandiflorum* transcriptome sequencing and annotation features

Features	Value
HiFi sequencing Iso-seq	
Number of Iso-seq reads	4,632,516
N50 HiFi reads	2,050 bp
Average Phred value	Q80
GC content of the Iso-seq reads (%)	44
RNA-seq—Illumina	
Number of reads (2 × 100 bp)	46,414,378
GC content of the reads (%)	44
BUSCO analysis (transcriptome)	embryophyta_odb10 (1,614)
Complete	98.7% (1,593)
Complete and single copy	22.5% (363)
Complete and duplicated	76.2% (1,230)
Fragmented	0.5% (8)
Missing	0.8% (13)
Genome annotation	
Number of genes	31,381
Number of CDSs (including isoforms)	46,671
Complete CDS	46,625
Start, no stop CDS	8
Stop, no start CDS	22
No stop, no start CDS	16
Mean gene length	3,374 bp
Mean CDS length	1,331 bp
Mean exons per gene	6
Mean introns per gene	5
tRNAs	446
snRNAs	976
miRNAs	109
% of genome covered by genes	25%
% of genome covered by CDS	14.70%
% of genome covered by TEs	53.93%
Class I elements	43.86%
LTR Gypsy	13.18%
LTR Copia	18.31%
LTR nonautonomous	12.37%
Non-LTR	0.58%
Class II elements	2.36%
TIRs	1.21%
Helitron	1.15%
Other repeats	7.13%
BUSCO analysis (annotation)	
Complete	99.8% (1,610)
Complete and single copy	59.5% (960)
Complete and duplicated	40.3% (650)
Fragmented	0.1% (1)
Missing	0.3% (3)

The gene arrangement and distribution in *T. grandiflorum* and *T. cacao* genomes show a similar pattern according to their closely related evolutionary ties. This pattern includes genes from various duplications (whole genome, tandem, proximal, transposed, dispersed) (Supplementary Table S6). Analysis of Ks values and the distribution of WGD-derived gene pairs within syntenic blocks employing Gaussian mixture models unveiled a distinct Ks peak at 2.5. This peak corresponds with the core eudicot γ whole-genome triplication (WGT) event (Fig. 2C). This observed peak is corroborated by prior studies that have identified the γ WGT event across a diverse range of plant species [58]. The core eudicot γ WGT is estimated to have occurred approximately 117 million years ago (mya) during the Lower Cretaceous [84]. This event predates the more recent species differentiation, which, according to Timetree of Life Database [79] and previous molecular dating studies [85], occurred at ∼14 mya for *Theobroma* species and ∼18 mya between the genera of *Theobroma* and *Herrania*, both during the Miocene epoch.

The cupuassu genome contains 402 genes that have originated through RNA-mediated duplication, referred to as retrocopies, comprising 197 chimeric genes, 37 pseudogenes, and 168 retrogenes. A comparative analysis of these retrocopies with *T. cacao* and *H. umbratica* highlighted unique retrocopies in each Theobromateae: 67 in *T. grandiflorum*, 50 in *T. cacao*, and 34 in *H. umbratica* (Supplementary Table S7). Interestingly, some of the unique retrocopies are linked to potential fruit and seed quality traits and plant development. For instance, a number of exclusive retrocopies in these species are related to serine/threonine–protein kinase, which is important for signal transduction and plays relevant roles in pathogen defense and fruit abscission [86]. Furthermore, retrocopies associated with chalcone metabolism in *T. grandiflorum* (TgrandC1074G00000001563) and embryo sac development in *T. cacao* (Tcacao-CriolloG00000031869) were also identified. Additionally, unique retrocopied transcription factors were noted, such as an auxin response factor in *T. grandiflorum* (TgrandC1074G00000000856) and WER-like transcription factors in *T. cacao* (Tcacao-CriolloG00000008811). *H. umbratica* unique retrocopies include genes linked to a caffeic acid 3-O-methyltransferase-like activity (HumbraticaG00000009034) and polygalacturonase (HumbraticaG00000026833), potentially affecting fruit traits.

In *T. cacao*, noncoding RNAs (ncRNAs) have been proposed as primary regulators of gene expression [11]. In cupuassu, our annotation identified 1,178 long noncoding RNAs (lncRNAs), 1,058 small nucleolar RNAs (snoRNAs), 446 transfer RNAs (tRNAs), 126 microRNAs (miRNAs), 48 small nuclear RNAs (snRNAs), and 17 small RNAs (sRNAs). Moreover, the primary sites for 5S and 45S ribosomal DNA (rDNA) were mapped to chromosomes 2 and 7, respectively, corroborating previous rDNA localization using fluorescence *in situ* hybridization [83]. Overall, ncRNAs are relatively evenly distributed across the chromosomes. Notably, chromosome 7 has the lowest counts of tRNAs, miRNAs, and sRNAs but holds the major rDNA (45S) locus (Supplementary Fig. S1 and Supplementary Table S8).

### TE distribution and impact in the cupuassu genome architecture and function

**Figure 3:**
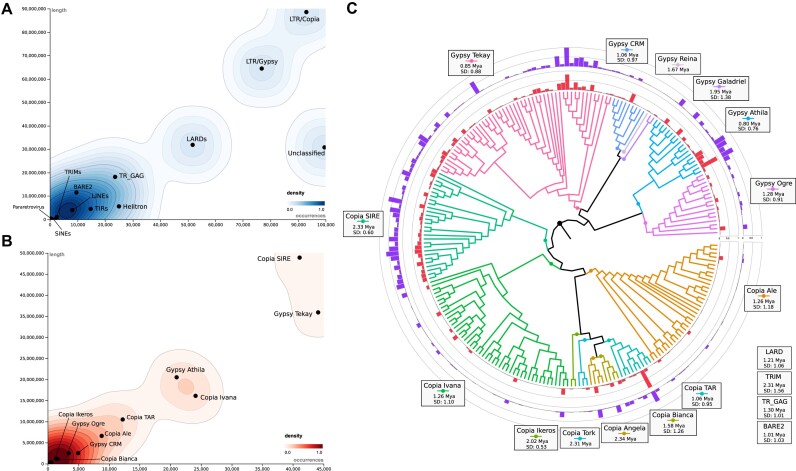
Transposable element distribution in *T. grandiflorum*. (A) Distribution of autonomous and nonautonomous TE from class I and class II. (B) Distribution of all evolutionary lineages of LTR elements. (C) Phylogenetic analysis and distribution of each full-length LTR element identified in *T. grandiflorum*. The age of LTR insertions was estimated using the default rate of 1.3 × 10^–8^ substitutions per site per year, making this calculation an approximate estimation.

TE and repetitive elements constitute roughly 54% of the *T. grandiflorum* genome. The most abundant TEs were LTR *Copia*, LTR *Gypsy*, and the nonautonomous LARD elements (Fig. 3A and Supplementary Table S9). Notably, LTR *Copia* SIRE and LTR *Gypsy* Tekay were the most prevalent lineages, accounting for up to 49 and 36 Mb of the genome (Fig. 3B). Evolutionarily, LTR *Copia* elements had 2 significant peaks of expansion at 0.3 and 1.8 mya, whereas the LTR *Gypsy* elements showed a single peak at around 0.3 mya (Supplementary Fig. S2). Comparative analyses reveal that the estimated ages of LTR expansions peaks in *T. grandiflorum, T. cacao*, and *H. umbratica* predate the species differentiation by more than 10 million years, as evidenced by previous molecular dating approaches [85]. This finding underscores the potential significance of LTR elements in driving genomic evolution postdivergence within the Theobromateae tribe. Moreover, the insertion ages of LTR *Gypsy* and *Copia* elements in the analyzed genomes generally exhibit patterns similar to those observed in several plant families, including flowering plants families Brassicaceae, Fabaceae, Musaceae, Poaceae, Salicaceae and Solanaceae, and also non-flowering plants as Funariaceae and Selaginellaceae [87].

While the LTR *Copia* SIRE and LTR *Gypsy* Tekay elements are notably abundant in *T. grandiflorum*, they display unique expansion pattern and ages (Fig. 3C). Almost all members of *Copia SIRE* exhibit expansion, whereas only a subset of *Gypsy Tekay* elements shows a similar expansive trend. In contrast, certain LTR lineages, particularly *Copia TAR* and *Gypsy Athila*, have undergone significant proliferative events, marking their distinctive expansion. Interestingly, despite the high membership of *Copia Ivana, Ale*, and *Gypsy Ogre*, these lineages exhibit limited proliferation. In contrast, the class II elements were less prominent, as observed in other plant genomes, including *T. cacao* [11, 88]. For instance, the MuDR/Mutator lineage is the most abundant, covering 881 Kb (0.27%) of the cupuassu genome.

The distribution of TEs across cupuassu chromosomes is uniform among all TE classes and lineages (Supplementary Table S8). The density of TEs around gene regions reflects their overall abundance in the genome, with LTR *Copia*, LTR *Gypsy*, and LARDs being concentrated near genes, typically located around 1.5 Kb at both up- and downstream (Supplementary Fig. S3). This distribution pattern supports the idea that TEs are advantageously located, rather than randomly, possibly impacting gene expression patterns and their regulator networks [89].

### *Theobroma grandiflorum* exhibits elevated syntenic relationships with cacao and *H. umbratica*

**Figure 4:**
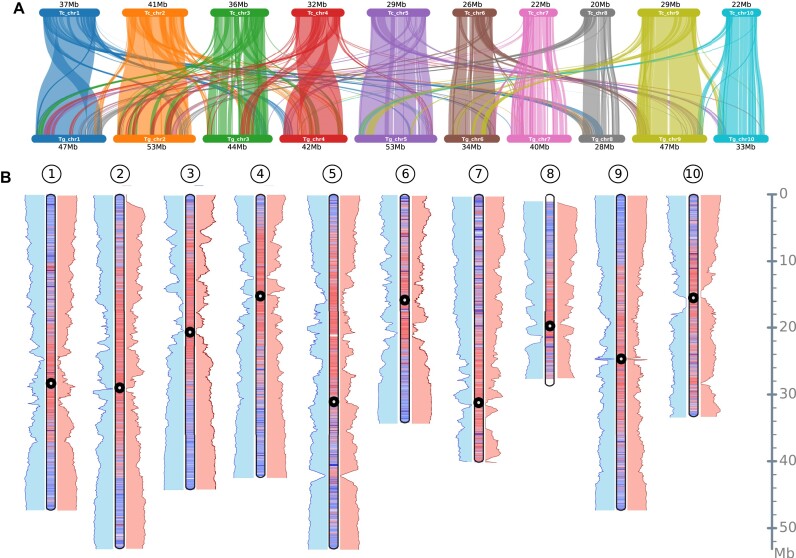
Comparative genomic analysis of *T. grandiflorum* with *T. cacao* and *H. umbratica*. (A) Macrosyntenic patterns between *T. grandiflorum* and *T. cacao*, revealing conserved genome structures. (B) Comparative idiogram map between *T. grandiflorum* and *T. cacao*, as well as between *T. grandiflorum* and *H. umbratica*. The idiograms illustrate gene-rich regions (blue), TE-rich regions (red), and potential location of centromeres (black circles) identified by the quarTeT and Centromics tools. Blue bars on the left of each idiogram represent microsynteny between *T. grandiflorum* and *T. cacao*, while red bars on the right indicate microsynteny between *T. grandiflorum* and *H. umbratica*.

At the macrosyntenic level, both *Theobroma* species exhibit significant genomic conservation, suggesting minimal rearrangements (Fig. 4A), an observation that corroborates with the published high-density cupuassu genetic map [18]. Notable variation occurs primarily within the pericentromeric and predicted centromeric regions, characterized by an elevated TE density, and other TE-dense regions (Fig. 4B). This pattern is consistent with what is commonly found in plant genomes, and it has been previously observed in the cacao genome [11].

A closer inspection at the microsyntenic level among *T. grandiflorum, T. cacao*, and *H. umbratica* reveals a marked gene synteny and collinearity, especially at the subtelomeric regions (Fig. 4B and Supplementary Fig. S4). These genomes conserve at least 65% of gene synteny (Supplementary Table S8). Interestingly, transposed gene pairs between these species are comparatively infrequent (around 7% on average).

### Microsyntenic insights into the self-incompatibility loci of *Theobroma* and *Herrania*

**Figure 5:**
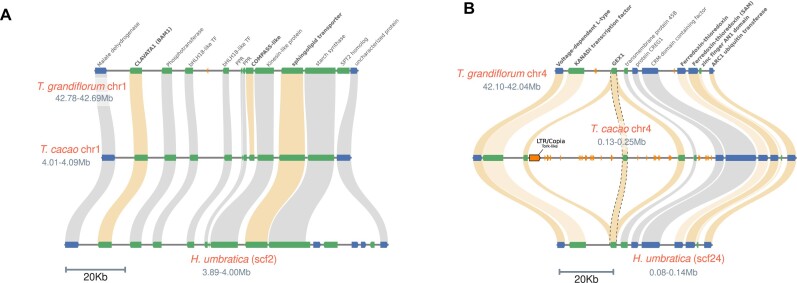
Microsyntenic analysis of the self-incompatibility loci (CH1 and CH4) in *Theobroma* and *Herrania*. (A) CH1 loci. (B) CH4 loci. Genes marked in bold are considered central to self-incompatibility reactions, as previously described [79]. The GEX1 locus, containing the complete and homologous genes, is marked with dotted lines.

Previous research identified 2 self-incompatibility loci in cacao, CH1 and CH4, with CH4 primarily linked to fruit drop [90]. Microsyntenic comparison of these loci in *T. grandiflorum* and *H. umbratica* revealed distinct patterns (Fig. 5A, B). CH1 is highly conserved across the 3 genomes, except for a missing COMPASS-like H3K4 histone methylase gene in *H. umbratica*, crucial in cellular network [91]. CH4, however, varies significantly; *T. grandiflorum* and *H. umbratica* sequences are conserved, but the one in *T. cacao* contains 2 additional truncated GEX1 gene copies (Supplementary Fig. S5), presumably affecting gametophyte and embryo development, as well as possibly affecting fruit setting and late incompatibility in *T. cacao* [90, 92]. The CH4 locus in cacao also features many TE remnants and a complete LTR-RT from the Copia/Tork lineage close to a truncated copy of GEX1.

Cupuassu and cacao notably differ for fruit abscission. Cupuassu fruits naturally abscise when ripe, whereas cacao fruits need to be harvested from the tree [93, 94]. We speculate that the multiple copies of the cacao GEX1 gene, including the 2 truncated ones, together with the proximity of TE at the CH4 loci, could either affect GEX1 expression or produce nonfunctional GEX1 proteins. This potential effect may be linked to the lack of fruit abscission phenotype in cacao, although this hypothesis needs further experimental investigation to be confirmed.

### Comparative analyses reveal exclusive cupuassu genes and distinct patterns of gene family expansion and contraction associated with fruit quality traits and defense mechanisms

**Figure 6:**
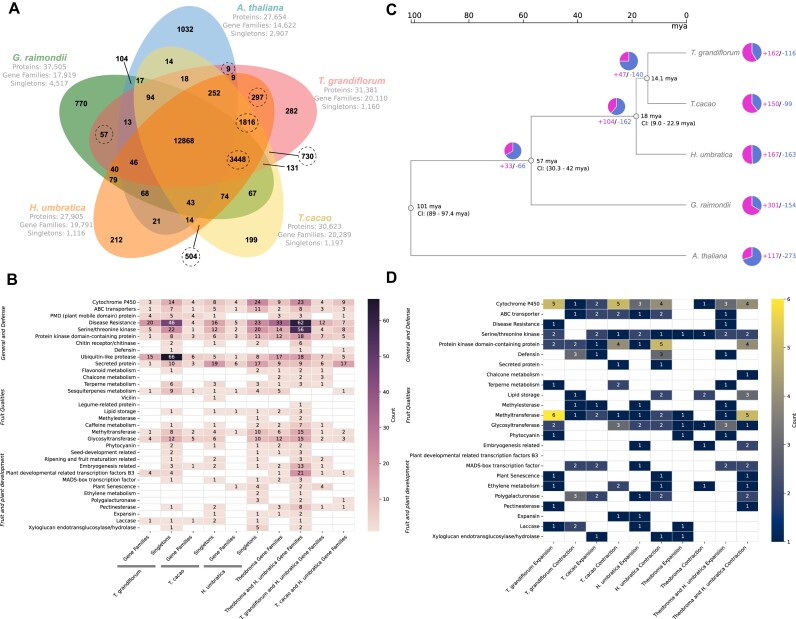
Comparative analyses across Malvaceae species focusing on functions related to plant differentiation, fruit and seed development, and organoleptic and physicochemical qualities. (A) A Venn diagram illustrates the shared and exclusive orthologous clusters (gene families) identified across 4 Malvaceae species and *Arabidopsis thaliana*. (B) The identification of gene families and singletons encompasses a range of functions with predicted roles in various aspects of plant and fruit development. These include cytochrome P450 and ABC transporters, which are pivotal in synthesizing secondary metabolites and nutrient uptake, respectively, influencing plant growth and fruit quality. Plant mobile domain (PMD) proteins and disease resistance genes play roles in stress response and plant health, indirectly impacting fruit quality. Serine/threonine kinase, protein kinase domain–containing proteins, and several metabolism-related genes (flavonoid, chalcone, terpene, sesquiterpenes) regulate pathways critical for plant growth, development, and the organoleptic properties of fruits. Genes related to defense mechanisms (chitin receptor/chitinase, defensin, ubiquitin-like protease) and cell wall composition (methylesterase, polygalacturonase, pectinesterase, expansin, laccase, xyloglucan endotransglucosylase/hydrolase) are also identified, reflecting their roles in maintaining plant health and influencing fruit texture and firmness. Furthermore, genes involved in seed development (vicilin, legume-related protein, lipid storage) and various transcription factors (including MADS-box) are noted for their influence on plant growth and developmental processes. (C) A phylogenetic tree delineates the evolutionary timeline of the Malvaceae species, with *A. thaliana* serving as the outgroup. An accompanying pie chart displays the proportions of gene families that have expanded or contracted, indicating evolutionary dynamics. The divergence time and its confidence interval, when available, were obtained from the TimeTree5 database. (D) The analysis of expanded and contracted gene families focuses on their common functions and roles, as detailed in B, shedding light on the evolutionary adaptations of these species.

A total of 282 exclusive gene families and 1,160 singletons were identified in *T. grandiflorum* (Fig. 6A), whereas 730 gene families are shared between *T. grandiflorum* and *T. cacao*, and 297 gene families are shared between *T. grandiflorum* and *H. umbratica*. Collectively, the three genomes share 1,816 gene families. The shared gene families among the three species exhibit only 2 significant GO enrichment: one related to pollen recognition (GO:0048544) and the other associated with protein localization to the cell surface (GO:0034394). Further GO enrichment analyses did not identify any statistically significant enrichment among the other shared and exclusive gene families. Among the exclusive and shared gene families and singletons, many are linked to fruit quality, maturation, development of organoleptic characteristics, general plant development, and resistance to pathogens (Fig. 6B and Supplementary Tables S10 and S11).

Moreover, the analysis of gene expansion and contraction revealed distinct patterns across Malvaceae (Fig. 6C). Although the GO enrichment analyses did not indicate any other statistically significant enrichment, we were able to determine specific gene functions related to important agronomical traits, indicating groups of gene families that were expanded and contracted in each species (Fig. 6D and Supplementary Table S12).

We found that unique profiles of singletons and gene families (both expanded and contracted) are primarily categorized as cytochrome P450, ABC transporters, and other functions related to plant development and pathogen defense. This indicates specific adaptations and responses to domestication, environmental changes, and response to various stresses. For instance, numerous gene families and singletons genes belonging the PMD domain-containing protein identified uniquely in *T. grandiflorum* and *T. cacao* likely play a role in developmental control [95], while the singletons genes encoding to chitin receptor/chitinase (i.e., TgrandC1074G00000003550 and TgrandC1074G00000000752) may be crucial for fungal resistance.

Exclusive gene profiles associated with fruit and seed quality, notably in lipid storage and secondary metabolite functions, were identified (Fig. 6B, D). The storage lipids in seeds are key components of the quality of cocoa butter and chocolate in cacao, as well as in cupulate and cosmetic products in cupuassu [11, 96]. Additionally, unique gene profiles involved in flavonoid, terpenoid, and sesquiterpene metabolism might contribute to the distinct aromas of cacao and cupuassu. Furthermore, different profiles in purine alkaloid metabolism could explain the flavor differences between both *Theobroma* species.

Moreover, distinct pattern of enzymes, such as methyltransferase, glycosyltransferase, and phytocyanin, were identified, all crucial to secondary metabolism and related to fruit traits. Methyltransferases are key in secondary metabolite metabolism (phenylpropanoids, flavonoids, alkaloids), affecting flavor, pulp, and seed testa color [97–99]. Glycosyltransferases, catalyzing glycosylation reactions for various substrates, including plant hormones and secondary metabolites, affect fruit ripening and seed development [100, 101]. Additionally, the unique gene pattern of phytocyanin, involved in growth and stress resilience [102], may be linked to the adaptability in challenging environmental conditions.

These findings corroborate the hypothesis that, despite a high number of shared gene families among Malvaceae genomes, each species exhibits unique gene families and singleton genes, and specific instances of gene family expansion and contraction, which affect developmental, defense, and adaptive functions, as well as biosynthetic pathways. Such gene families and singletons are potentially associated with the unique fruit morphologies observed, which, in turn, may affect the specific traits of each Theobromateae species, like flavor, aroma, and bioactive compound content.

### Arrangement and distribution of duplicated genes reveals evolutionary insights into fruit and seed quality and defense mechanism origins

**Figure 7:**
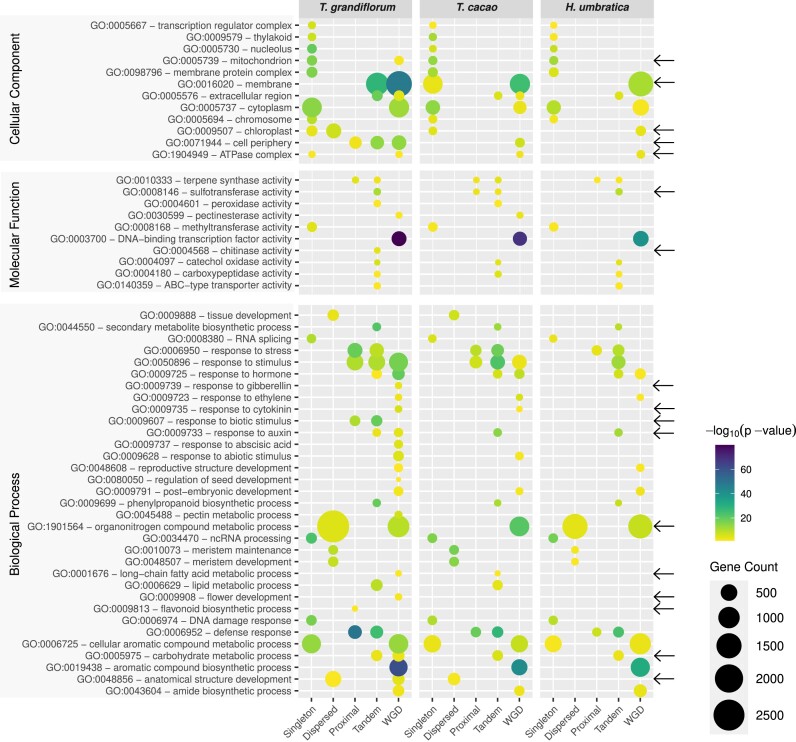
Gene Ontology enrichment and comparative analysis across *T. grandiflorum, T. cacao*, and *H. umbratica*. Black arrows highlight GO terms that are exclusively enriched in *T. grandiflorum*, in either duplicated genes or singletons. These terms provide insights into the unique biological processes, cellular components, and molecular functions connected with fruit and seed quality and defense mechanism that are particularly prominent in *T. grandiflorum* compared to the other species.

The gene arrangement and distribution in the genome of the *T. grandiflorum, T. cacao*, and *H. umbratica* was evaluated by comprehensive GO enrichment analyses (Fig. 7 and Supplementary Table S13). The analysis centered on GO terms, both directly and indirectly associated with fruit and seed quality as well as defense mechanisms, and it uncovered distinct functional variations across different types of gene duplications. The duplications include whole-genome duplications (WGD events), as well as tandem, proximal, dispersed, and singleton duplicates, highlighting the complex evolutionary dynamics influencing these key traits.

### Cellular component ontology trends and variations

Most of the selected “cellular component” GOs tend to be predominantly enriched in singleton genes in the three genomes. However, *T. grandiflorum* uniquely exhibited enrichment in WGD-derived genes associated with “mitochondrion” (GO:0005739) and tandem genes linked to “membrane” (GO:0016020). In contrast, *T. cacao* showed an enrichment of “membrane-associated” (GO:0016020) singleton genes. Additionally, *T. grandiflorum* also showed enrichment to “chloroplast” (GO:0009507) in dispersed duplicates and “cell periphery” (GO:0071944) in proximal and tandem duplicated genes, with an exclusive enrichment of “ATPase complex” (GO:1904949) in singleton genes (cellular component panel of Fig. 7).

### Molecular function ontology trends and variations

Generally, “molecular function” GOs show enrichment in tandem duplicates. In particular, “methyltransferase activity” (GO:0008168), which is implicated in various physiological processes, including fruit development [97], is found to be enriched among singleton genes. Furthermore, “DNA-binding transcription factor activity” (GO:0003700) is enriched in WGD-derived genes. Notably, “sulfotransferase activity” (GO:0008146), potentially influencing flavonoid metabolism [103], was enriched in both proximal and tandem duplicates of *T. cacao*. In contrast, this activity was enriched exclusively in tandem duplicates in the cupuassu and *H. umbratica* genomes. Furthermore, “Chitinase activity” (GO:0004568), likely associated with defense against fungal pathogens [21], was enriched only in *T. grandiflorum* tandem duplicated genes (molecular function panel of Fig. 7).

### GO terms related to fruit and seed traits

Numerous GO terms potentially related to fruit and seed traits were identified as enriched in duplicated genes. This is particularly prominent for “terpene synthase activity” (GO:0010333), which shows enrichment in both tandem and proximal duplicates. Additionally, GO terms associated with “secondary metabolite biosynthesis” (GO:0044550), “lipid metabolic process” (GO:0006629), “phenylpropanoid biosynthesis” (GO:0009699), “catechol oxidase activity” (GO:0004097), and “carboxypeptidase activity” (GO:0004180) were predominantly enriched in tandem genes. Notably, GO terms related to the “organonitrogen compound metabolic process” (GO:1901564) and “long-chain fatty acid metabolic process” (GO:0001676) showed diverse enrichment patterns across species. This finding is particularly noteworthy due to the distinct differences in fatty acid composition between cacao and cupuassu seeds. Specifically, cacao seeds exhibit a higher concentration of saturated fatty acids, predominantly palmitic and stearic acids, followed by desaturated fatty acids, including oleic and linoleic acids [104]. In contrast, cupuassu and *Herrania* are characterized by a richness in desaturated fatty acids and long-chain fatty acids [105]. Moreover, “flavonoid biosynthetic process” (GO:0009813) was observed to be enriched in proximal duplicated genes exclusively within *T. grandiflorum*. This indicates a divergent evolutionary trajectory in comparison to that of cacao, wherein flavonoids are ubiquitously present in cacao seeds. Such an observation lends additional support to the hypothesis of unique evolutionary pathways and distinct domestication processes characterizing these species.

### GO terms related to fruit aroma and ripening process

The “cellular aromatic compound metabolic process” (GO:0006725), which may affect fruit aroma and plant defense [106], was enriched in singletons and WGD-derived genes in all three species. “Pectinesterase activity” (GO:0030599), potentially related to fruit ripening and cell wall fortification [107], was enriched in WGD-derived genes of *T. grandiflorum* and *T. cacao* but not in *H. umbratica* (Fig. 7). The enrichment of “pectinesterase activity” (GO:0030599) in WGD-derived genes may suggests a possible evolutionary advantage in the postduplication genomic landscape of *T. cacao* and *T. grandiflorum*, reflecting in variations in their fruit maturation timelines, cell wall composition, and responses to environmental stresses.

### GO terms related to fruit morphology and hormonal response

Genes associated with “meristem maintenance and development” (GO:0048507 and GO:0010073) and “anatomical structure development” (GO:0048856) were predominantly enriched in dispersed duplicates in the three Theobromateae species. In contrast, *T. grandiflorum* genes related to “seed development” (GO:0080050) and “flower development” (GO:0009908) showed enrichment in WGD-derived genes. These WGD-derived genes were also enriched in terms related to “hormonal responses” (GO:0009725) and “DNA binding transcription factor activity” (GO:0003700), while singleton genes showed enrichment in “ncRNA processing” (GO:0034470), “RNA splicing” (GO:0008380), and “DNA damage responses” (GO:0006974). These findings indicate that tandem and WGD-derived genes may have contributed to the evolution of complex reproductive structures and the fine-tuning of hormonal regulation. For instance, the “response to gibberellin” (GO:0009739) is exclusively enriched in the WGD-derived genes of *T. grandiflorum*. Furthermore, in *T. grandiflorum*, the “response to auxin” (GO:0009733) is enriched in both tandem and WGD duplicates, whereas it appears to be exclusively enriched in WGD-derived genes of *T. cacao* and *H. umbratica*. Meanwhile, singleton genes might play a pivotal role in gene regulation and response to environmental stimuli, underscoring the multifaceted genetic mechanisms underlying plant development and adaptability (Fig. 7).

### GO terms related to defense response and stress reaction

Genes involved in “defense response” (GO:0006952) and “response to stress” (GO:0006950) were enriched in proximal and tandem repeated genes in the three species . The “response to biotic stimulus” (GO:0009607) was also enriched in these gene types, whereas the “response to abiotic stimulus” (GO:0009628) was more prevalent in WGD-derived genes. These observed gene enrichment patterns suggest a functional specialization among gene duplication types in plant response mechanisms. For instance, proximal and tandem repeated genes are primarily associated with defense responses and stress management, indicating their crucial role in immediate and localized reaction to biotic stressors. Conversely, genes derived from WGD show a higher association with responses to abiotic stimuli, suggesting that WGD events may have equipped plants with enhanced capabilities to adapt to a broader range of environmental challenges. This dichotomy underscores the complexity of plant defense mechanisms and highlights the evolutionary significance of gene duplication in developing versatile and robust response strategies to both biotic and abiotic stresses.

### Positively selected retained dispersed, proximal, and tandem duplications: potential drivers of fruit and pathogen resistance evolution?

From a general evolutionary perspective, genes derived from WGD events are typically ancient and often well integrated into the existing genetic framework, which allows ample time to functionally diverge [108]. In contrast, genes from tandem, proximal, and dispersed duplications are generally younger, often emerging in response to environmental challenges and stressors [109, 110] and possibly influenced by the domestication process. In parallel, singleton genes, often originating from genome fractionation events after WGD, play crucial roles in core cellular functions and essential physiological processes [111–113].

During the evolutionary timeframe and through domestication, new genes were likely created by duplication and lost over time. Interestingly, some duplicated genes are retained and can acquire new roles (neofunctionalization) or specialize in aspects of their original function (subfunctionalization), contributing to morphological innovations and the development of new functionalities, including the enhancement of disease resistance, and increased stress adaptability [112, 114].

**Figure 8:**
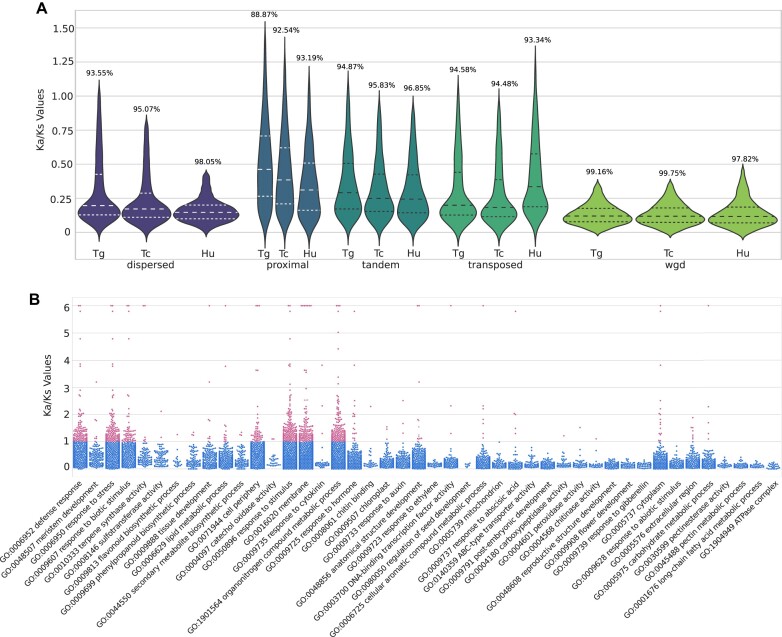
Positive selection analysis of duplicated genes in *T. grandiflorum, T. cacao*, and *H. umbratica*. (A) A violin plot displays the distribution of the Ka/Ks ratios for gene pairs resulting from dispersed, proximal, and tandem duplication in *T. grandiflorum, T. cacao*, and *H. umbratica*. The number above each plot indicates the percentage of duplicated genes under purifying selection. (B) A swarmplot illustrates the Ka/Ks ratio distributions for selected Gene Ontology (GO) terms associated with fruit traits and defense mechanisms in *T. grandiflorum*. This plot provides insights into the selective pressures acting on genes related to these specific functions.

To contextualize these evolutionary processes, we evaluated the Ka/Ks rate across different gene duplication types (Fig. 8A). A significant majority of duplicated genes in *H. umbratica* (97.39%), *T. cacao* (95.37%), and *T. grandiflorum* (93.85%) are under purifying selection, a trend consistent with observations in other plant species [58]. WGD-derived genes in all species exhibit strong purifying selection with a mean Ka/Ks of 0.132. Dispersed duplicates largely follow this trend (mean Ka/Ks of 0.165), with occasional peaks suggesting a balance between purifying and positive selection.

Although the majority of proximal (mean Ka/Ks = 0.444), tandem (mean Ka/Ks = 0.331), and transposed (mean Ka/Ks = 0.329) gene pairs demonstrate a trend to be under purifying selection, there is an evident trend toward greater tolerance to variation. These findings support the hypothesis of postspeciation adaptation in these gene groups, likely related to diversification or domestication effect.

Indeed, upon detailed examination, a significant portion of duplicated genes in *T. grandiflorum* (6.15%), *T. cacao* (4.62%), and *H. umbratica* (2.6%)—associated with GO terms related to plant defense, fruit, and seed traits—were found to be under positive selection (Fig. 8B, Supplementary Fig. S6, Supplementary Table S14).

For instance, in the evolutionary battle between plants and their adversaries, defense-related genes often undergo positive selection [115, 116]. This is exemplified by several clusters of tandemly duplicated genes linked to defense responses and plant disease resistance, which demonstrate strong positive selection in *T. grandiflorum* (139 genes), *T. cacao* (40 genes), and *H. umbratica* (17 genes). Notably, in *T. grandiflorum*, a significant concentration of these genes is found in chromosomes 6, 7, and 10 (Supplementary Fig. S7).

A cluster of genes on chromosome 6 of *T. grandiflorum* corresponds with an identified cupuassu WBD-resistance quantitative trait locus (QTL) [18]. Within this QTL, the *TgPR3* gene encoding a chitinase was associated with WBD resistance [21]. The sequenced cupuassu genome displays the chitinase gene (TgrandC1074G00000024418), which is encircled by a multitude of disease resistance genes located within this QTL. Some of these disease resistance genes are tandemly duplicated and exhibit signs of positive selection, suggesting a robust assembly of disease resistance genes in this specific QTL (Supplementary Table S15). However, it is essential to recognize that the cupuassu genome under analysis is from a *M. perniciosa*–susceptible genotype. As a result, the evolutionary gene pattern identified may not necessarily confer resistance to WBD but could potentially be associated with resistance to other pathogens.

In the “terpene synthase activity” (GO:0010333), tandem arrays encoding a number of delta-cadinene synthases are under positive selection across the three species. This enzyme plays a role in sesquiterpene biosynthesis, crucial for plant defense and the production of compounds like gossypol in cotton seeds [117, 118]. It was also considered a key candidate for studying cacao–insect resistance interplay [11]. Interestingly, *T. grandiflorum* uniquely harbors tandem repeated genes encoding a probable terpene synthase (TgrandC1074G00000007568 and TgrandC1074G00000007569), hinting at regulatory role in terpenoid biosynthesis with potential ramifications for fruit aroma and flavor. Conversely, *T. cacao* possesses positively selected tandem repeated genes encoding a potential nerolidol synthase (Tcacao-CriolloG00000024422 and Tcacao-CriolloG00000024423). In cacao, this enzyme contributes to linalool biosynthesis, producing volatile monoterpenes. Linalool can be abundant in cacao seeds and is responsible for their floral aroma in certain genotypes [119]. In grapes, this enzyme enhances the aroma of certain varieties [120]. Additionally, in rice, it is associated with the production of an antibacterial compound effective against bacterial pathogens [121].

Within the “flavonoid biosynthetic pathway” (GO:0009813) of *T. grandiflorum*, a gene encoding a positively selected tandem duplicated naringenin 2-oxoglutarate 3-dioxygenase (TgrandC1074G00000004751 and TgrandC1074G00000004753) may emerges as pivotal in specific flavonoid, anthocyanidin, catechin, and proanthocyanidin biosynthesis. Given naringenin-documented broad-spectrum biological impacts on human health [122], it is conceivable that this gene plays a role in the distinct antioxidant properties of cupuassu [123], further influencing the fruit unique taste and aroma.

Another set of tandemly duplicated genes under positive selection, potentially linked to fruit and seed characteristics, involves those engaged in the “lipid metabolic process” (GO:0006629). Both cupuassu and cacao present a distinct pattern of tandemly duplicated genes, possibly related to their unique seed properties. Specifically, cupuassu has a positively selected and tandemly duplicated gene related to lipid storage in fruits, known as patatin (TgrandC1074G00000017909 and TgrandC1074G00000017911). Originally identified in potato (*Solanum tuberosum* L.) tubers, patatin is renowned for its antioxidant potential [124] and its exceptional nutritional value, making it an appealing food additive due to its solubility and emulsifying properties [125, 126].

In contrast, *T. cacao* features a tandem duplicated phospholipase A1 positively selected (Tcacao-CriolloG00000024071 and Tcacao-CriolloG00000024072), which could modulate the fruit phospholipid profile. For instance, this phospholipase may be involved in linoleic acid metabolism [127], central to the production of desaturated fatty acids present in cacao-derived chocolates [104]. Meanwhile, *T. grandiflorum*, displays a tandem repeated gene encoding a fatty acyl-CoA reductase enzyme (TgrandC1074G00000003252 and TgrandC1074G00000003253), potentially affecting the lipid content and composition of seeds, impacting wax biosynthesis [128] and, by extension, the fruit cuticle, water retention, and shelf life.

Furthermore, *T. cacao* possesses 2 dispersed duplicated and positively selected pectinesterases (Tcacao-CriolloG00000016685 and Tcacao-CriolloG00000021823) that might play a significant role in the ripening of cacao fruit. Interestingly, neither *T. grandiflorum* nor *H. umbratica* exhibit positively selected pectinesterases. This observation may be associated with the behavior of cacao tree fruits, which do not fall when ripe but remain attached to the tree until manually harvested [93].

## Conclusions

Recent advancements in long-read sequencing, chromatin interaction technologies, and comparative genomics have significantly enriched our understanding of genome evolution, particularly in the *Theobroma* genus, and have contributed to insights into phenotypic variation [13, 129]. These tools facilitate in-depth analysis of plant development and the determinants of disease resistance, offering substantial biotechnological implications. They are becoming increasingly essential in crop breeding to address challenges such as climate change and food security.

Our study presents a chromosome-scale genome assembly of *T. grandiflorum*, enhancing its genetic resources for breeding and sustainable horticulture. We have uncovered evolutionary insights into the origins of genes linked to key agronomic traits. Furthermore, we identified unique gene families and singletons in Malvaceae species, which may be instrumental in organ development, defense, adaptation, and distinctive fruit traits. The variation in gene presence or absence (and gene family expansion and contraction) among these species might be associated with unique mechanisms of gene retention and loss, which in turn are closely related to the generation of phenotypic diversity and innovation [130]. Concurrently, we revealed that many retained duplicated genes related to plant defense, fruit, and seed production are under positive selection. This finding also aligns with known processes of phenotypic novelty emergence, leading to speciation and diversification [112, 131, 132]. By providing a comprehensive candidate genes list, we aim not only to support breeding initiatives but also to deepen our understanding of the cupuassu genome biology. We believe that the results presented here lay the groundwork for advanced functional genomic interventions and tailored cultivation methods. This could potentially enhance species conservation and farmer productivity, thereby further impacting the Amazonian bioeconomy. In conclusion, our findings offer valuable insights into the unique evolutionary pathways and domestication of *T. grandiflorum* and *T. cacao*, particularly in terms of pathogen resistance, fruit and seed development, and adaptive strategies after diversification.

## Additional Files

**Supplementary Information 1**. HMW DNA extraction; sequencing QC; bioinformatics procedures used to annotate *Theobroma grandiflorum, T. cacao*, and *Herrania umbratica* genomes; and additional notes.

**Supplementary Fig. S1**. ncRNA distribution in *Theobroma grandiflorum* chromosomes.

**Supplementary Fig. S2**. LTR insertion time of *Gypsy* and *Copia* elements. (A) *T. grandiflorum*, (B) *T. cacao*, and (C) *H. umbratica*. The vertical black line represents the median, and the dotted line represents the mean. The age of LTR insertions was estimated using the default substitution rate of 1.3 × 10^–8^ substitutions per site per year, making this calculation an approximate estimation.

**Supplementary Fig. S3**. TE_density analyses of all *T. grandiflorum* chromosomes.

**Supplementary Fig. S4**. (A) Microsynteny and colinearity example of subtelomeric regions of *T. grandiflorum, T. cacao*, and *H. umbratica*. (B) Microsynteny and colinearity example of pericentromeric regions of *T. grandiflorum, T. cacao*, and *H. umbratica*. Blue represents genes in the forward direction, green indicates genes in the reverse direction, and orange denotes transposable elements (TEs).

**Supplementary Fig. S5**. Alignment of the *GEX1* gene from *CH4* loci.

**Supplementary Fig. S6**. Boxplot and swarmplot showing the the Ka/Ks ratio distributions of the selected GO terms associated with fruit traits and defense mechanisms. (A) *T. cacao*. (B) *H. umbratica*.

**Supplementary Fig. S7**. Genomic mapping of plant disease resistance genes in *T. grandiflorum* chromosomes. Genes under positive selection are shown in red. The cupuassu WBD-resistant QTL is shown in blue.

**Supplementary Table S1**. GenBank SRA accession numbers used for transcriptome assembly. (A) All *T. cacao* RNA-seq data used. (B) *H. umbratica* RNA-seq data used.

**Supplementary Table S2**. Genome assembly statistics and completeness scores of the three genomes (BUSCO scores were retrieved using embryophyta_odb10.

**Supplementary Table S3**. Summary of gaps on the *T. grandiflorum* chromosomes (the genomic coordinates includes ∼500-bp boundaries).

**Supplementary Table S4**. Summary of telomeres on the *T. grandiflorum* chromosomes.

**Supplementary Table S5**. Summary of centromeres on the *T. grandiflorum* chromosomes.

**Supplementary Table S6**. Genome annotation features and statistics of the 3 Theobromateae genomes.

**Supplementary Table S7**. Retrocopies identified in *T. grandiflorum, T. cacao*, and *H. umbratica*, with associated raw data.

**Supplementary Table S8**. Genome structural features and statistics for each *T. grandiflorum* chromosome.

**Supplementary Table S9**. Transposable elements summary table and statistics identified of the 3 Theobromateae genomes.

**Supplementary Table S10**. Exclusive gene families identified for each Theobromateae genome analyzed.

**Supplementary Table S11**. Singletons identified in each Theobromateae genome analyzed.

**Supplementary Table S12**. Expanded and contracted gene families identified in each Theobromateae genome analyzed.

**Supplementary Table S13**. GO enrichment analyses raw data.

**Supplementary Table S14**. Genes and GO terms identified as positively selected by Ka/Ks analysis.

**Supplementary Table S15**. Gene content and features of cupuassu WBD-resistant QTL.

## Abbreviations

BUSCO: Benchmarking Universal Single-Copy Orthologs; CDS: coding sequence; CTAB: cetyltrimethylammonium bromide; FP: frosty pod; GO: Gene Ontology; HiC: chromosome conformation capture techniques; HMW: high molecular weight; Ka: nonsynonymous nucleotide substitutions; Ks: synonymous substitutions; LAI: LTR Assembly Index; LARD: large retrotransposon derivatives; lncRNA: long noncoding RNA; LTR-RT: long terminal repeat retrotransposon; miRNA: microRNA; mya: million years ago; QTL: quantitative trait locus; rDNA: ribosomal DNA; snoRNA: small nucleolar RNA; sRNA: small RNA; snRNA: small nuclear RNA; T2T: telomere-to-telomere; TE: transposable element; TRIM: terminal-repeat retrotransposons in miniature; tRNA: transfer RNA; WBD: witches’ broom disease; WGD: whole-genome duplication; WGT: whole-genome triplication.

## Supplementary Material

giae027_supplement

giae027_GIGA_D_23_00404_Original_Submission

giae027_GIGA_D_23_00404_Revision_1

giae027_GIGA_D_23_00404_Revision_2

giae027_Response_to_Reviewer_Comments_Original_Submission

giae027_Response_to_Reviewer_Comments_Revision_1

giae027_Reviewer_1_Report_Original_Submission Xupo Ding -- 2/21/2024

giae027_Reviewer_1_Report_Revision_1 Xupo Ding -- 3/29/2024

giae027_Reviewer_2_Report_Original_Submission Jian-Feng Mao -- 2/26/2024

## Data Availability

The *T. grandiflroum* sample (GenBank BioSample SAMN37717187) was included at the National Genetic Heritage and Associated Traditional Knowledge Management System (SisGen) under accession #A2A72C6 [133]. The complete genome was deposited at GenBank, BioProject PRJNA691024; the raw reads are available at GenBank Sequence Read Archive (SRA) under the following accession numbers: SRR28330360, SRR28297999, SRR28289108, SRR26316970, and SRR26316971. The genome sequence, gene models, and functional annotation files (GFF3s and FASTAs) are also available at our genome browser web service [134]. Supporting data are also available via the *GigaScience* database, GigaDB [135].
