# Supplementary material for: Genomic decoding of Theobroma grandiflorum (cupuassu) at chromosomal scale: evolutionary insights for horticultural innovation
Source: Gigascience. 2024 Jun 5;13:giae027. doi: 10.1093/gigascience/giae027 (PMC11152179; doi:10.1093/gigascience/giae027)
Supplement: giae027_supplement [file giae027_supplement.zip › FigureS5.pdf]

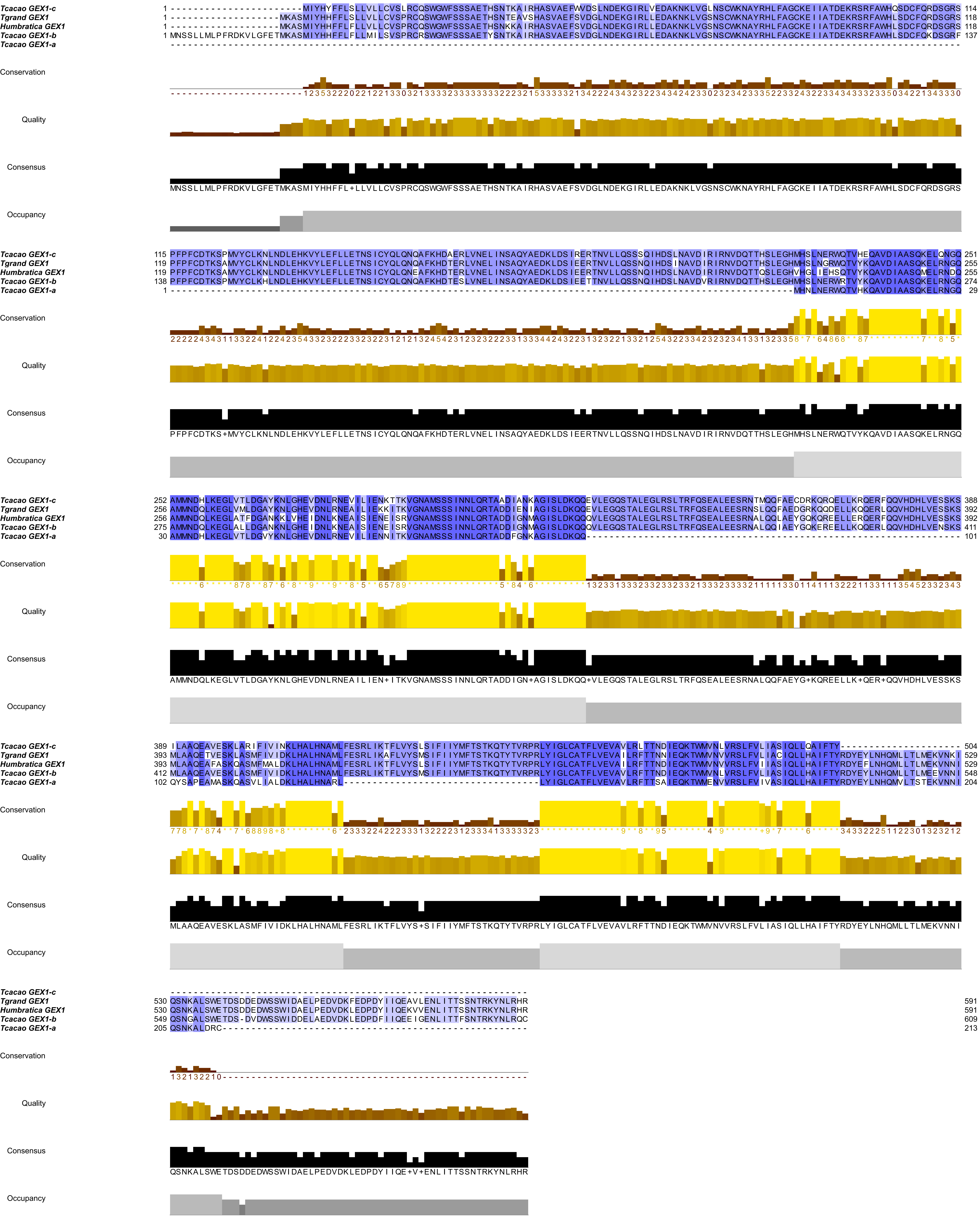

Tcacao GEX1-c 1 --MIYHYFFLSLLVLLCVSLRCQSWGWFSSSAETHSNTKAIRHASVAEFWVDSLNDEKGIRLVEDAKNKLVGLNSCWKNAYRHLFAGCKEIIATDEKRSRFAWHQSDCFQRDSGRS 114
Tgrand GEX1 1 --MKASMIYHHFFLSLLVLLCVSPRCQSWGWFSSSAETHSNTEAVSHASVAEFSVDGLNDEKGIRLLEDAKNKLVGSNSCWKNAYRHLFAGCKEIIATDEKRSRFAWHLSDCFQRDSGRS 118
Humbratica GEX1 1 --MKASMIYHHFFLFLLVLLCVSPRCQSWGWFSSSAETHSNKKAIRHASVAEFSVDGLNDEKGIRLLEDAKNKLVGSNSCWKNAYRHLFAGCKEIIATDEKRSRFAWHLSDCFQRDSGRS 118
Tcacao GEX1-b 1 MNSSLLMLPFRDKVLGFETMKASMIYHHFFLLMILSVSPRCRSWGWFSSSAETYSNTKAIRHASVAEFSVDGLNDEKGIRLLEDAKNKLVGSNSCWKNAYRHLFAGCKEIIATDEKRSRFAWHLSDCFQKDSGRF 137
Tcacao GEX1-a --
Conservation
Quality
Consensus
MNSSLLMLPFRDKVLGFETMKASMIYHHFFL+LLVLLCVSPRCQSWGWFSSSAETHSNTKAIRHASVAEFSVDGLNDEKGIRLLEDAKNKLVGSNSCWKNAYRHLFAGCKEIIATDEKRSRFAWHLSDCFQRDSGRS
Occupancy
Tcacao GEX1-c 115 PFPFCDTKSPMVYCLKNLNDLEHKVYLEFLLETNSICYQLQNQAFKHDAERLVNELINSAQYAEDKLDSIRERTNVLLQSSSQIHDSLNAVDIRIRNVDQTTHSLEGHMHSLNERWQTVHEQAVDIAASQKELQNGQ 251
Tgrand GEX1 119 PFPFCDTKSAMVYCLKNLNDLEHKVYLEFLLETNSICYQLQNQAFKHDTERLVNELINSAQYAEDKLDSIEERTNVLLQSSNQIHDSINAVDIRIRNVDQTTHSLEGHMHSLNGRWQTVYKQAVDIAASQKELRNGQ 255
Humbratica GEX1 119 PFPFCDTKSAMVYCLKNLNDLEHKVYLEFLLETNSICYQLQNEAFKHDTERLVNELINSAQYAEDKLDSIEERTNVLLQSSNQIHDSLNAVDIRIRNVDQTTQSLEGHVHGLIEHSQTVYKQAVDIAASQMELRNGQ 255
Tcacao GEX1-b 138 PFPFCDTKSAMVYCLKNLHDLEHKVYLEFLLETNSICYQLQNQAFKHDTESLVNELINSAQYAEDKLDSIEETTNVLLQSSNQIHDSLNAVDVRIRNVDQTTHSLEGHMHSLNERWRTVYKQAVDIAASQKELRNDQ 274
Tcacao GEX1-a 1 --MHNLNERWQTVHKQAVDIAASQKELRNGQ 29
Conservation
Quality
Consensus
PFPFCDTKS+MVYCLKNLNDLEHKVYLEFLLETNSICYQLQNQAFKHDTERLVNELINSAQYAEDKLDSIEERTNVLLQSSNQIHDSLNAVDIRIRNVDQTTHSLEGHMHSLNERWQTVYKQAVDIAASQKELRNGQ
Occupancy
Tcacao GEX1-c 252 AMMNDHLKEGLVTLDGAYKNLGHEVDNLRNEVILIENKTTKVGNAMSSSINNLQRTAADIANKAGISLDKQQEVLEGQSTALEGLRSLTRFQSEALEESRNTMQQFAECDRKQRQELLKRQERFQQVHDHLVESSKS 388
Tgrand GEX1 256 AMMNDQLKEGLVMLDGAYKNLGHEVDNLRNEAILIEKKITKVGNAMSSSINNLQRTADDIENIAGISLDKQQEVLEGQSTALEGLRSLTRFQSEALEESRNSLQQFAEDGRKQQDELLKQQERLQQVHDHLVESSKS 392
Humbratica GEX1 256 AMMNDQLKEGLATFDGANKKLVHEIDNLKNEAISIENEISRVGNAMSSSINNLQRTADDIGNMAGISLDKQQQVLEGQSTALEGLRSLTRFQSEALEESRNALQQLAEYGQKQREELLERQERLQQVHDHLVESSKS 392
Tcacao GEX1-b 275 AMMNDQLKEGLALLDGANKNLGHEIDNLKNEAISIENEISRVGNAMSSSINNLQRTADDIGNMAGISLDKQQQVLEGQSTALEGLRSLTRFQSEALEESRNALQQIAEYGQKEREELLKQQERLQQVHDHLVENSKS 411
Tcacao GEX1-a 30 AMMNDHLKEGLVTLDGVYKNLGHEVDNLRNEVILIENNITKVGNAMSSSINNLQRTADDFGNKAGISLDKQQ-- 101
Conservation
Quality
Consensus
AMMNDQLKEGLVTLDGAYKNLGHEVDNLRNEAILIEN+ITKVGNAMSSSINNLQRTADDIGN+AGISLDKQQ+VLEGQSTALEGLRSLTRFQSEALEESRNALQQFAEYG+KQREELLK+QER+QQVHDHLVESSKS
Occupancy
Tcacao GEX1-c 389 ILAAQEAVESKLARIFIVINKLHALHNAMLFESRLIKTFLVYSLSIFIIYMFTSTKQTYTVRPRLYIGLCATFLVEVAVLRLTTNDIEQKTWMVNLVRSLFVLIASIQLLQAIFTY-- 504
Tgrand GEX1 393 MLAAQETVESKLASMFIVIDKLHALHNAMLFESRLIKAFLVYSMSIFIIYMFTSTKQTYTVRPRLYIGLCATFLVEVAILRFTTNNIEQKTWMVNVVRSLFVLIACIQLLHAIFTYRDYEYLNHQMLLTLMEKVNKI 529
Humbratica GEX1 393 MLAAQEAFASKQASMFMALDKLHALHNAMLFESRLIKTFLVYSLSIFIIYMFTSTKQTYTVRPRLYIGLCATFLVEVAILRFTTNDIEQKTWMVNVVRSLFVIIASIQLLHAIFTYRDYEFLNHQMLLTLMEKVNNI 529
Tcacao GEX1-b 412 MLAAQEAVESKLASMFIVIDKLHALHNAMLFESRLIKTFLVYSMSIFIIYMFTSTKQTYTVRPRLYIGLCATFLVEVAVLRFTTNDIEQKTWMVNLVRSLFVLIASIQLLHAIFTYRDYEYLNHQMLLTLMEEVNNI 548
Tcacao GEX1-a 102 QYSAPEAMASKQASVLIALDKLHALHNARL--LYIGLCATFLVEVAVLRFTTSAIEQKTWMENVVRSLFVIVASIQLLHAIFTYRDYEYLNHQMVLTSTEKVNNI 204
Conservation
Quality
Consensus
MLAAQEAVESKLASMFIVIDKLHALHNAMLFESRLIKTFLVYS+SIFIIYMFTSTKQTYTVRPRLYIGLCATFLVEVAVLRFTTNDIEQKTWMVNVVRSLFVLIASIQLLHAIFTYRDYEYLNHQMLLTLMEKVNNI
Occupancy
Tcacao GEX1-c
Tgrand GEX1 530 QSNKALSWETDSDDEDWSSWIDAELPEDVDKFEDPDYIIQEAVLENLITTSSNTRKYNLRHR 591
Humbratica GEX1 530 QSNKALSWETDSDDEDWSSWIDAELPEDVDKLEDPDYIIQEKVVENLITTSSNTRKYNLRHR 591
Tcacao GEX1-b 549 QSNGALSWETDS-DVDWSSWIDDELAEDVDKLEDPDFIIQEEIGENLITTFSNTRKYNLRQC 609
Tcacao GEX1-a 205 QSNKALDRC-- 213
Conservation
Quality
Consensus
QSNKALSWETDSDDEDWSSWIDAELPEDVDKLEDPDYIIQE+V+ENLITTSSNTRKYNLRHR
Occupancy
