# Supplementary material for: Genomic decoding of Theobroma grandiflorum (cupuassu) at chromosomal scale: evolutionary insights for horticultural innovation
Source: Gigascience. 2024 Jun 5;13:giae027. doi: 10.1093/gigascience/giae027 (PMC11152179; doi:10.1093/gigascience/giae027)
Supplement: giae027_supplement [file giae027_supplement.zip › FigureS6.pdf]

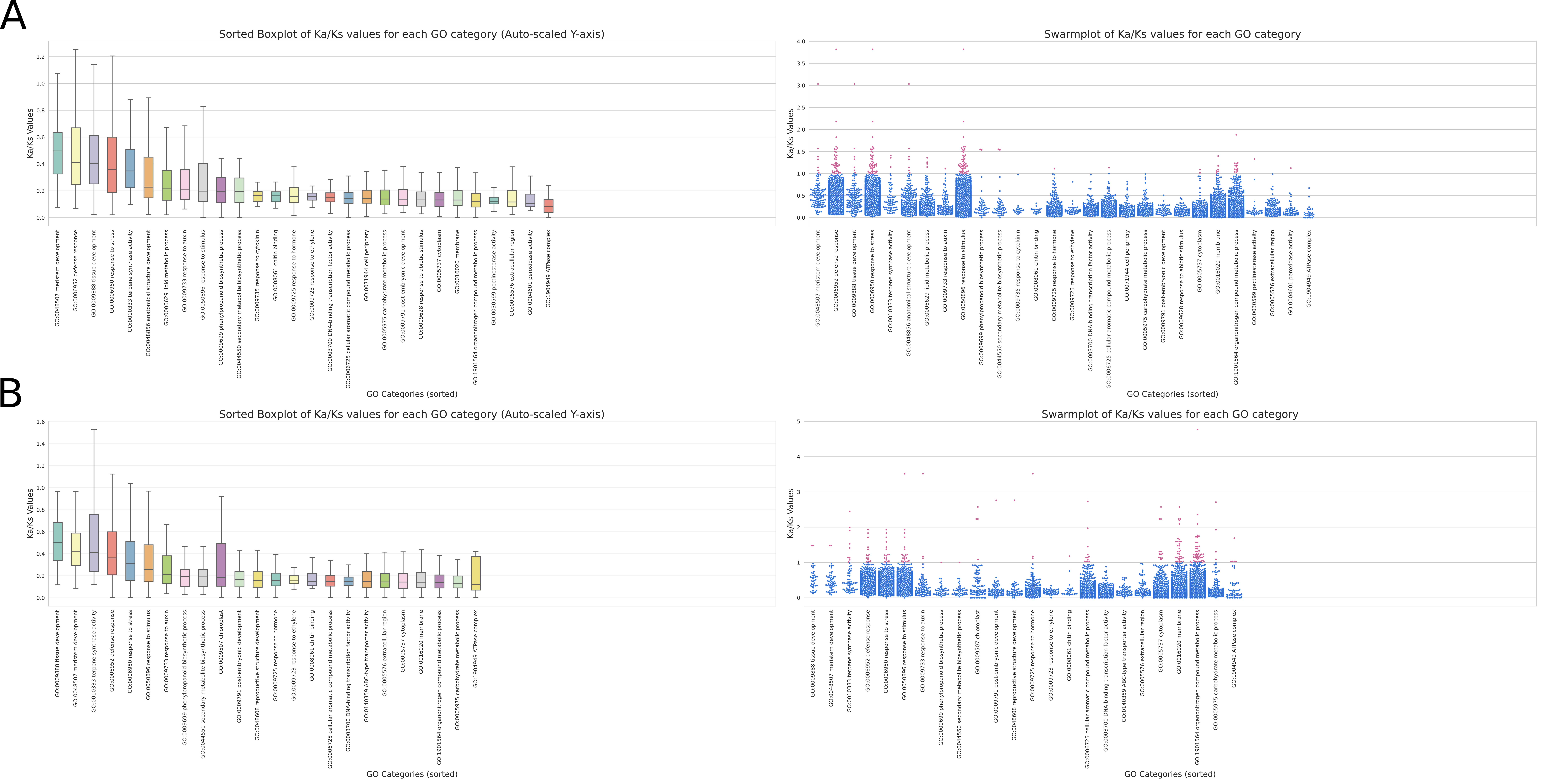
A
Sorted Boxplot of Ka/Ks values for each GO category (Auto-scaled Y-axis)
Ka/Ks Values
GO Categories (sorted)
GO:0048507 meristem development
GO:0006952 defense response
GO:0009888 tissue development
GO:0006950 response to stress
GO:0010333 terpene synthase activity
GO:0048856 anatomical structure development
GO:0006629 lipid metabolic process
GO:0009733 response to auxin
GO:0050896 response to stimulus
GO:0009699 phenylpropanoid biosynthetic process
GO:0044550 secondary metabolite biosynthetic process
GO:0009735 response to cytokinin
GO:0008061 chitin binding
GO:0009725 response to hormone
GO:0009723 response to ethylene
GO:0003700 DNA-binding transcription factor activity
GO:0006725 cellular aromatic compound metabolic process
GO:0071944 cell periphery
GO:0005975 carbohydrate metabolic process
GO:0009791 post-embryonic development
GO:0009628 response to abiotic stimulus
GO:0005737 cytoplasm
GO:0016020 membrane
GO:1901564 organonitrogen compound metabolic process
GO:0030599 pectinesterase activity
GO:0005576 extracellular region
GO:0004601 peroxidase activity
GO:1904949 ATPase complex
Swarmplot of Ka/Ks values for each GO category
Ka/Ks Values
GO Categories (sorted)
B
Sorted Boxplot of Ka/Ks values for each GO category (Auto-scaled Y-axis)
Ka/Ks Values
GO Categories (sorted)
GO:0009888 tissue development
GO:0048507 meristem development
GO:0010333 terpene synthase activity
GO:0006952 defense response
GO:0006950 response to stress
GO:0050896 response to stimulus
GO:0009733 response to auxin
GO:0009699 phenylpropanoid biosynthetic process
GO:0044550 secondary metabolite biosynthetic process
GO:0009507 chloroplast
GO:0009791 post-embryonic development
GO:0048608 reproductive structure development
GO:0009725 response to hormone
GO:0009723 response to ethylene
GO:0008061 chitin binding
GO:0006725 cellular aromatic compound metabolic process
GO:0003700 DNA-binding transcription factor activity
GO:0140359 ABC-type transporter activity
GO:0005576 extracellular region
GO:0005737 cytoplasm
GO:0016020 membrane
GO:1901564 organonitrogen compound metabolic process
GO:0005975 carbohydrate metabolic process
GO:1904949 ATPase complex
Swarmplot of Ka/Ks values for each GO category
Ka/Ks Values
GO Categories (sorted)
