# Supplementary material for: Genomic decoding of Theobroma grandiflorum (cupuassu) at chromosomal scale: evolutionary insights for horticultural innovation
Source: Gigascience. 2024 Jun 5;13:giae027. doi: 10.1093/gigascience/giae027 (PMC11152179; doi:10.1093/gigascience/giae027)
Supplement: giae027_supplement [file giae027_supplement.zip › Supplementary_Information-1-GigaSciences-review3.pdf]

Genomic decoding of *Theobroma grandiflorum* (cupuassu) at chromosomal scale: Evolutionary insights for horticultural innovation

Rafael Moysés Alves[1*], Vinicius A. C. de Abreu[2*], Rafaely Pantoja Oliveira[3], João Victor dos Anjos Almeida[3], Mauro de Medeiros de Oliveira[3], Saura R. Silva[4], Alexandre R. Paschoal[5,6], Sintia S.de Almeida[2], Pedro A. F. de Souza[2], Jesus A. Ferro[3], Vitor F. O. Miranda[4], Antonio Figueira[7], Douglas S. Domingues[8], Alessandro M. Varani[3]

[1] Embrapa Amazônia Oriental, 66095-903 Belém, PA, Brazil

[2] Laboratório de Bioinformática e Computação de Alto Desempenho (LaBioCad), Faculdade de Computação (FACOMP), Universidade Federal do Pará, 66075-110 Belém, PA, Brazil

[3] Departamento de Biotecnologia Agropecuária e Ambiental, Universidade Estadual Paulista (UNESP), Faculdade de Ciências Agrárias e Veterinárias, 14884-900 Jaboticabal, SP, Brazil.

[4] Departamento de Biologia, Universidade Estadual Paulista (UNESP), Faculdade de Ciências Agrárias e Veterinárias, 14884-900 Jaboticabal, SP, Brazil.

[5] Departamento de Ciência da Computação (DACOM), Grupo de e Bioinformática e Reconhecimento de Padrões (bioinfo-cp), Universidade Tecnológica Federal do Paraná (UTFPR), 80230-901 Cornélio Procópio, PR, Brazil

[6] Artificial Intelligence and Informatics, The Rosalind Franklin Institute, OX110QX Didcot, UK

[7] Centro de Energia Nuclear na Agricultura (CENA), Universidade de São Paulo, 13416-000, Piracicaba, SP, Brazil

[8] Departamento de Genética, Universidade de São Paulo (USP), Escola Superior de Agricultura Luiz de Queiroz (ESALQ), 13418-900, Piracicaba, SP, Brazil

* These authors contributed equally to this work

Corresponding author: alessandro.varani@unesp.br

Running title: *Theobroma grandiflorum genome*

List of Supplementary Information Provided

Supplementary Information 1. HMW DNA extraction, Sequencing QC, Bioinformatics procedures used to annotate *Theobroma grandiflorum, T. cacao* and *Herrania umbratica* genomes, and additional notes.

Figures

Figure S1. ncRNA distribution in *Theobroma grandiflorum* chromosomes.

Figure S2. LTR insertion time of *Gypsy* and *Copia* elements. **A.** *Theobroma grandiflorum,* **B.** *T. cacao,* and **C.** *Herrania umbratica.* The vertical black line represents the median, and the dotted line represents the mean. The age of LTR insertions was estimated using the default substitution rate of 1.3×10^{-8} substitutions per site per year, making this calculation an approximate estimation.

Figure S3. TE_density analyses of all *Theobroma grandiflorum* chromosomes.

Figure S4. A. Microsynteny and colinearity example of subtelomeric regions of *Theorboma grandiflorum*, *T. cacao* and *Herrania umbratica*, **B.** Microsynteny and colinearity example of pericentromeric regions of *T. grandiflorum*, *T. cacao* and *H. umbratica.* Blue represents genes in the forward direction, green indicates genes in the reverse direction, and orange denotes transposable elements (TEs).

Figure S5. Alignment of the *GEX1* gene from *CH4* loci generated on Jalview (Procter et al., 2021).

Figure S6. Box-plot and swarmplot showing the the Ka/Ks ratio distributions of the selected GO terms associated with fruit traits and defense mechanisms. A. *Theobroma cacao*, B. *Herrania umbratica*.

Figure S7. Genomic mapping of plant disease resistance genes in *Theobroma grandiflorum* chromosomes. Genes under positive selection are shown in red. The cupuassu WBD-resistant QTL is shown in blue.

Tables

Table S1. GenBank SRA accession numbers used for transcriptome assembly. **A.** All *Theobroma cacao* RNAseq data used. **B.** *Herrania umbratica* RNAseq data used.

Table S2. Genome assembly statistics and completeness scores of the three Theobromateae genomes (BUSCO scores were retrieved using embryophyta_odb10).

Table S3 Summary of gaps on the *T. grandiflorum* chromosomes (the genomic coordinates includes ~500bp boundaries).

Table S4. Summary of telomeres on the *T. grandiflorum* chromosomes.

Table S5. Summary of centromeres on the *T. grandiflorum* chromosomes.

Table S6. Genome annotation features and statistics of the three Theobromateae genomes.

Table S7. Retrocopies identified in *Theobroma grandiflorum*, *T. cacao*, and *Herrania umbratica,* with associated raw data.

Table S8. Genome structural features and statistics for each *Theobroma grandiflorum* chromosome.

Table S9. Transposable elements summary table and statistics identified of the three Theobromateae genomes

Table S10. Exclusive gene families identified for each Theobromateae genome analyzed.

Table S11. Singletons identified in each Theobromateae genome analyzed.

Table S12. Expanded and contracted gene families identified in each Theobromateae genome analyzed.

Table S13. GO enrichment analyses raw data.

Table S14. Genes and GO terms identified as positively selected by Ka/Ks analysis.

Table S15. Gene content and features of cupuassu WBD-resistant QTL.

1. Supplementary Information

1.1. DNA and RNA Sequencing and Quality Check

The High Molecular Weight (HMW) DNA, extracted from fresh leaves using the modified CTAB protocol, yielded a concentration of 174 ng/μL, resulting in a total volume of 200 μL and a total amount of 34.8 μg. The absorbance ratios at 260/280 and 260/230 were 1.74 and 1.43, respectively. The size of the DNA fragments, analyzed using Contour-clamped Homogeneous Electric Field (CHEF) electrophoresis, along with digest quality control, demonstrated the high quality of the obtained HMW DNA (Figure 1).

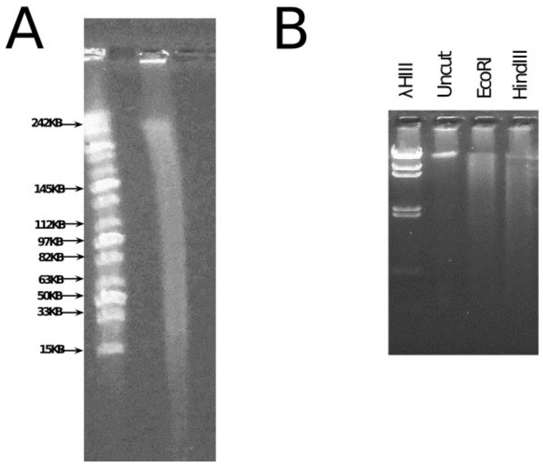

Figure 1. A. Size profile on CHEF. **B.** Digest QC.

For the genome assembly, the PacBio Sequel II sequencing produced 1.9 million reads, totaling 30 Gbp, with lengths varying from 43 bp to 48 Kb and an N50 of 15 Kb. The PHRED scores were high, consistently above 50, as illustrated in Figure 2.

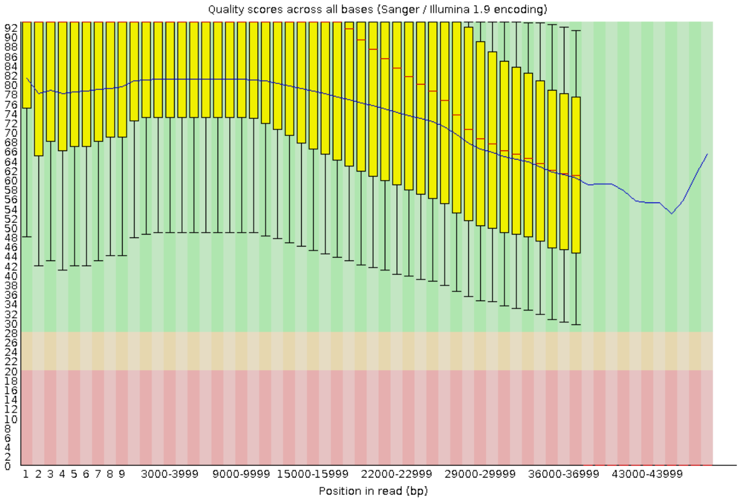

Figure 2. FastQC (**https://github.com/s-andrews/FastQC**) report of the DNA sequencing.

For the Iso-Seq sequencing, half of a PacBio SMRT cell produced 4.6 million reads, totaling 8.1 Gbp, with lengths ranging from 85 to 12,235 bp and an N50 value of 2 Kb. The mean PHRED score exceeded 40. In contrast, Illumina sequencing yielded 46 million paired-end reads, totaling 9 Gbp, each 2x100 bp, with an average PHRED score greater than 32 (Figure 3 and Figure 4). The obtained RNA concentration was 652 ng/µL, in a total volume of 50 µL, and 32.6 µg of total amount, with a RNA Integrity Number (RIN) of 7.7.

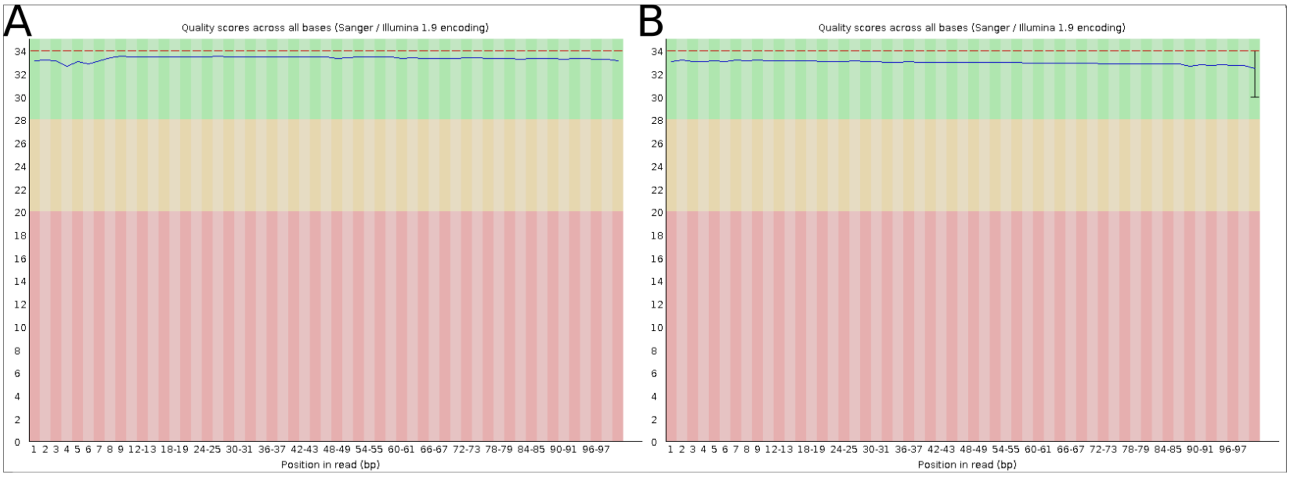

Figure 3. FastQC report of the RNAseq Illumina sequencing. **A.** forward reads, **B.** reverse reads.

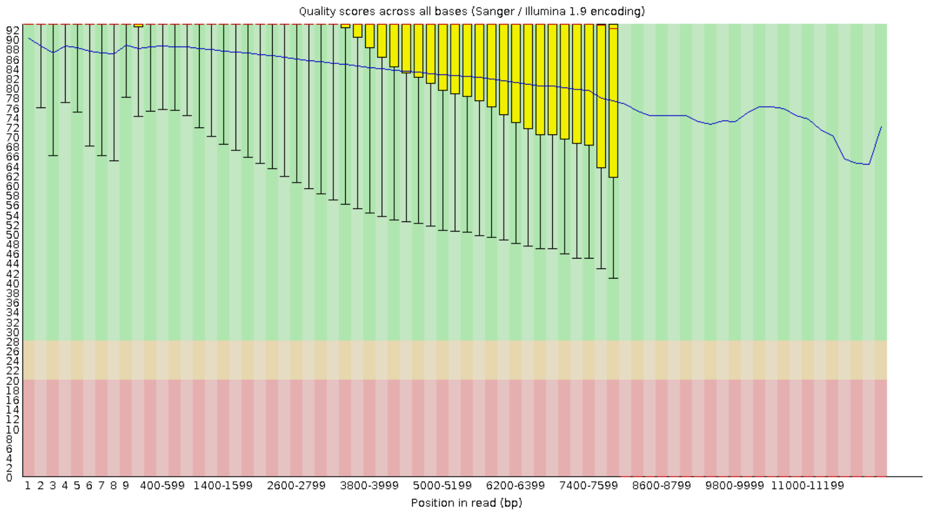

Figure 4. FastQC report of the Iso-Seq sequencing.

For the Hi-C sequencing, the Illumina NovaSeq platform generated 490 million paired-end reads with a PHRED score exceeding 30 (Figure 5).

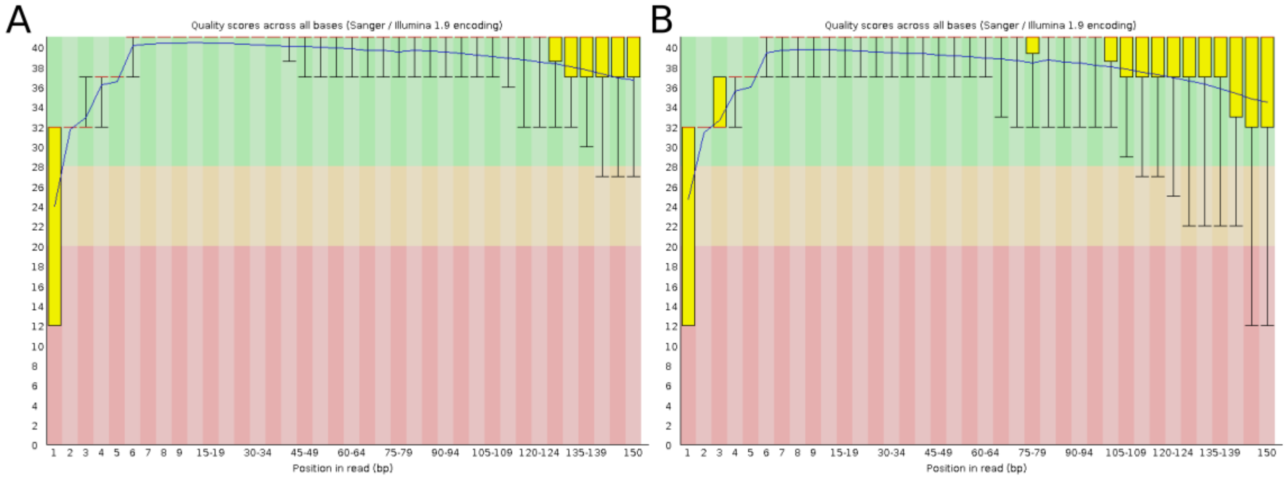

Figure 5. FastQC report of the Illumina HiC sequencing. **A.** forward reads, **B.** reverse reads.

1.2. Transcriptome Assembly

The results of the transcriptome assemblies for *T. grandiflorum*, *T. cacao*, and *H. umbratica*, assembled using the PASA v2.5.3 pipeline [2] are presented in Table 1.

Table1. Summary of the transcriptome assembly.

	T. grandiflorum	*T. cacao*	*H. umbratica*
Total assembled size	160,477,900 bp	579,838,728 bp	672,868,818 bp
Number of contigs	107,001	325,853	338,956
Mean contig size	1,500 bp	1,779 bp	1,985 bp
Median contig size	1,200 bp	848 bp	901 bp
N50	2,316 bp	3,579 bp	4,085 bp
L50	23,595	50,540	54,060
%GC	40.75	39.40	39.64
**BUSCO analysis (embryophyta_odb10)**			
Complete	98.7% (1,593)	99.7% (1610)	99.4% (1,605)
Complete and single copy	22.5% (363)	3.7% (60)	2.9% (47)
Complete and duplicated	76.2% (1,230)	96.0% (1,550)	96.5% (1,558)
Fragmented	0.5% (8)	0% (0)	0.2% (3)
Missing	0.8% (13)	0.3% (4)	0.4% (6)

1.3. Genome Annotation

In the first annotation stage, all Transposable Elements (TEs) were detected and annotated using a modified version of the EDTA pipeline [3] (based on v2.0.1), named hereafter as “Plant_Annotation_TEs”, and available and fully documented at following GitHub repository: https://github.com/amvarani/Plant_Annotation_TEs. This pipeline uses default parameters for identifying LTR elements, with the exception of setting the maximum distance between LTRs to 20,000 bp. Additionally, a second round of LTRharvest is conducted without specifying a motif. In summary, the parameters of LTR_FINDER were maximum distance between LTRs: 20000, minimum distance between LTRs: 1000, maximum LTR Length: 7000, minimum LTR Length: 100, length of exact match pairs: 20, match score: 0.85 and output format: 2. LTRharvest parameters were minimum LTR Length: 100, maximum LTR Length: 7000, maximum distance between LTRs: 20000, minimum length for each TSD: 4, maximum length for each TSD: 6, motif: TGCA, maximum number of mismatches in motif: 1, similarity threshold: 85, number of nucleotides to be searched for TSDs: 10, minimum seed length for exact repeats: 20. The second LTRharvest round employ the same parameters except the *motif* which is unset to find non-canonical motifs. The

LTR_FINDER and LTRharvest prediction are integrated with LTR_retriever in the EDTA pipeline, and the non-canonical motifs detected in the second LTRharvest round is integrated with the LTR_FINDER *-nonTGCA* flag. It is important to note that setting the maximum distance between LTRs to 20,000 bp, which allows for the prediction of longer internal regions, would lead to an increase in the size of intact elements. This, in turn, would result in a higher LTR Assembly Index (LAI) value [4].

This modified pipeline is also capable to deal with SINE and LINE structural identification using the AnnoSINE (commit: 26301e9) [5] and MGEScan-non-LTR v3.0.0 [6], while providing autonomous LTR elements full annotation at superfamily and lineages according the nomenclature proposed by Orozco-Arias et al. [7] using TEsorter v1.4.1 [8] with the 80-80-80 (identity-coverage-length) rule, and non-automous LTR elements full annotation (*e.g*. LARD, TRIM, TR_GAG, and BARE-2). The classification and annotation of non-autonomous LTR elements were based on the complete structure of the elements, as previously proposed [9]. This pipeline also allows to date the insertion time of each LTR elements using EDTA and to investigate the evolutionary history of those elements by applying a phylogenetic approach based on IQ-TREE2 v2.0.7 inference [10] using maximum likelihood, and to generate a soft-masked genome for structural gene annotation. Given the unavailability of a specific mutation rate (r) for many plants, including the *Theobroma* genus, we adopted the default r value of 1.3×10^{-8} substitutions per site per year provided by EDTA, which is typically applied to grasses, to estimate the insertion time of each LTR element. Therefore, the analysis of insertion time represents an approximate estimation.

For structural and functional gene annotation, the second stage generates multiple protein and transcriptome-based evidences using BRAKER 1+2 v3.0.4 [11], BRAKER3 v3.0.4 [12], TSEBRA v1.1.2 [13], Exonerate v2.4.0 [14], GALBA v1.0.7 [15] + miniprot v0.12 [16], GeMoMa v1.9 [17], all benchmarked by the BUSCO v5.4.5 using the embryophyta_odb10 database [18]. Only evidence showing BUSCO completeness score above 90% (protein mode) were considered for

further processing. The approved evidence and transcript structures previously determined using the PASA pipeline were integrated using EvidenceModeler v1.1.0 [19], and further processed in two rounds of the PASA v2.5.3 pipeline to annotate untranslated regions (UTRs), annotation correction, and identification and classification of all identified splicing variations.

A post-processing step was implemented to eliminate false-positive gene predictions that displayed hits to structurally determined TEs as identified in the "Plant_Annotation_TEs" pipeline and potential TE domains according to TEsorter v1.4.1. Moreover, false-positive genes were also removed only if they met all of the following criteria: structures shorter than 250 bp; lack of signal peptides as determined by SignalP 6.0 [20]; no transmembrane domains according to Phobius [21]; no alignments with RNA-seq and Iso-Seq data; and no sequence similarity by BLAST search [22] to entries in the UniProt [23] or NCBI RefSeq [24] plant databases. The BUSCO completeness scores were employed to evaluate the predicted proteins. The gene structure annotation was deemed acceptable only if the score equaled or exceeded the BUSCO genome score.

The approved structural annotation is complemented with the spatial gene arrangement information (*e.g.*, singleton, dispersed, proximal and tandem duplicated, WGD-derived, or transposed genes) generated by MCScanX (commit: b1ca533) [25] and, along with retrocopy identification by RetroScan (commit: cba0f4e) [26] and DupGen_finder (commit: 8001838) [27]. Considering the potential role of retrocopied genes in functional diversification [28], a comparative analysis was conducted, overlapping cupuassu retrocopies with those identified in *T. cacao* or *H. umbratica*

Functional annotation was generated through a BLAST search against public plant databases, such as UniProt (the dataset was accessed in July 2023 from the online database), NCBI RefSeq (the dataset was accessed in July 2023 from the online database), PlantTFDB v5.0 [29], NLR Atlas (the dataset was accessed in July 2023 from the online database) [30] and PRGdb v4.0 [31]. EggNOG-mapper v2.1.4-2-4-gb493df4 [32] and InterProScan v5.61-93.0 [33] to provide

additional functional annotation, including Gene Ontology (GOs), Enzyme Commission number (ECs), Carbohydrate-Active enZYme (CAZyme), PFAM, and InterPro IDs for each gene and detected isoform. Metabolic gene clusters are predicted using PlantiSMASH v1.0 [34]. Functional annotation was performed using Blast2GO Basic v6.0 [35]. GOs terms irrelevant to the Viridiplantae clade were subsequently filtered out via the Blast2GO filtering utility. Validation and the generation of the final GFF3 file, encompassing all annotation data, were facilitated by the AGAT (Another Gtf/Gff Analysis Toolkit) package v1.2.0 (https://github.com/NBISweden/AGAT).

The non-coding RNAs (ncRNAs) were annotated using two strategies. The first strategy relied on a BLAST similarity search against the RNAcentral database (version 22) [36]. The second strategy employed a structural search using the Infernal tool v1.1.5 [37] together with the curated RFAM database (version 14.9, 11/2022, encompassing 4,108 families) [38]. Results were filtered based on specific thresholds: low-quality BLAST outcomes were characterized by a query coverage and identity below 95%, while Infernal outcomes were deemed low-quality if they had a bitscore below the covariance model threshold (parameter-cut_ga). The results from both strategies were then integrated to produce the final ncRNA annotation.

The final annotation output includes annotated GFF3 and FASTA nucleotide and protein files, visualized using the JBrowse2 tool v2.0 [39]. In-house scripts were used to generate the annotation stats. Further details and instructions of the structural and functional annotation pipeline used in this study are provided in Figure 5, or in our GitHub repository: https://github.com/amvarani/Plant_Annotation. These same procedures were employed to annotate the *T. cacao* v2 (Criollo cultivar) [40] and *H. umbratica* (Fairchild cultivar) (https://www.ncbi.nlm.nih.gov/datasets/genome/GCF_002168275.1/) genomes.

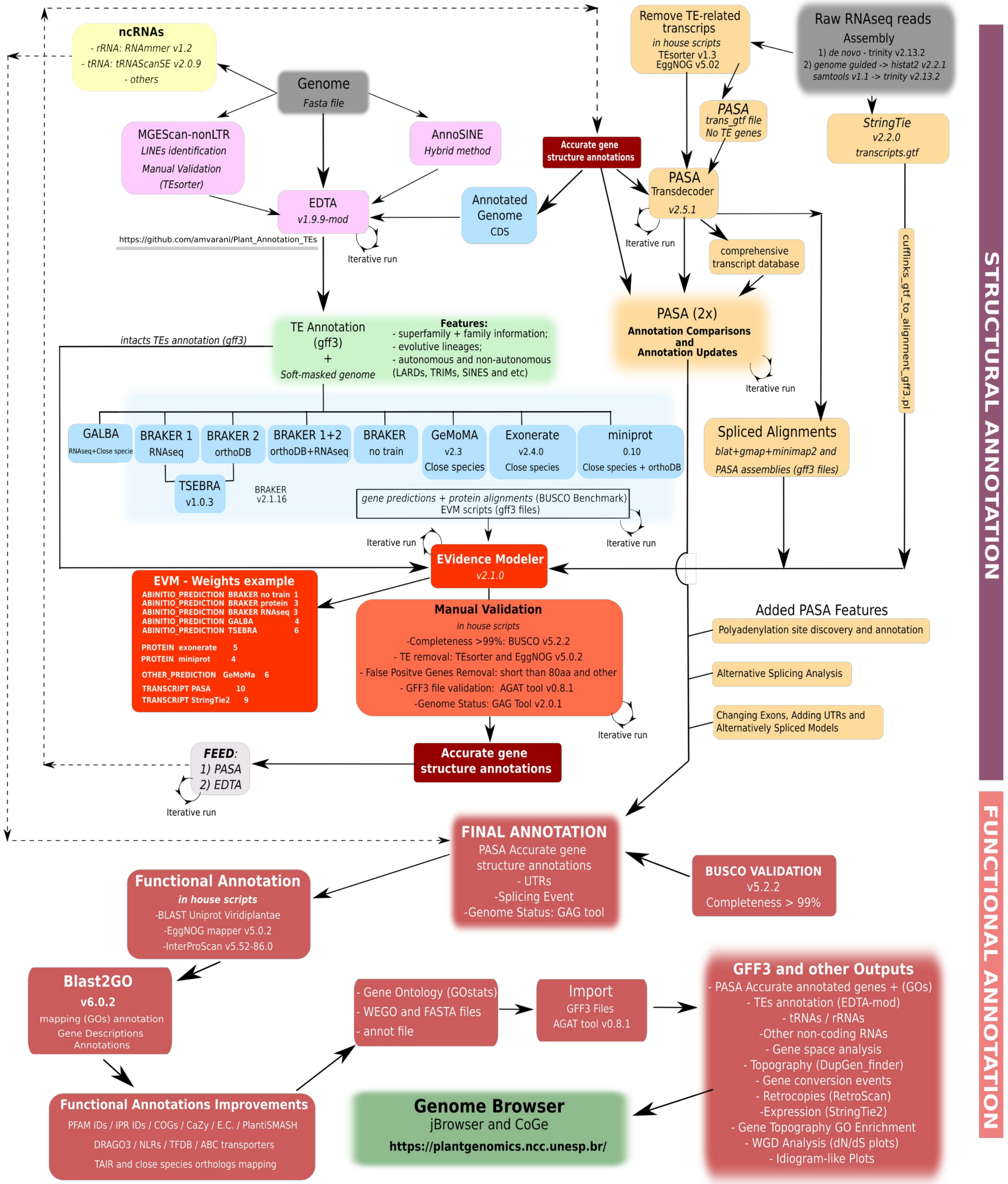

Figure 5. genome annotation pipeline employed in this study. For higher resolution and additional details, please refer to https://github.com/amvarani/Plant_Annotation.

1.4 General Considerations Regarding Comparative Approaches Among *Theobroma grandiflorum*, *T. cacao* v2, and *Herrania umbratica* cultivar Fairchild

The chromosomes and pseudomolecules of *Theobroma cacao* v2 (Belizian Criollo B97-61/B2 cultivar) [40] and *Herrania umbratica* cultivar Fairchild (BioProject: PRJNA383741) are notably shorter than those of *T. grandiflorum*, particularly in the pericentromeric regions (Figure 6). The assembled genomes of *T. cacao* and *H. umbratica* account for only about 70-71% of the estimated genome sizes, as determined by flow cytometry [41,42]. However, previous research indicated minimal variation in chromosome size between *T. cacao* and *T. grandiflorum* [43]. This discrepancy suggests that the genome assemblies of *T. caca*o and *H. umbratica* may be incomplete or unresolved, particularly in complex and highly repetitive regions such as centromeres and pericentromeric regions.

Given that the genomes of *T. cacao* and *H. umbratica* were assembled using earlier sequencing technologies, which are less capable of resolving complex genomic regions, these observations are not surprising. The advances in long-read sequencing technologies have significantly enhanced our ability to resolve intricate genomic regions. This is evident in the fourth version of the *Musa acuminata* genome [44], and in the resolution of the *T. grandiflorum* genome using HiFi reads and HiC technology.

Therefore, it is relevant to point out that a comparative framework among these three species should consider the distinct sequencing and assembly strategies utilized. These methodologies may have a direct impact on the structural genomic comparison.

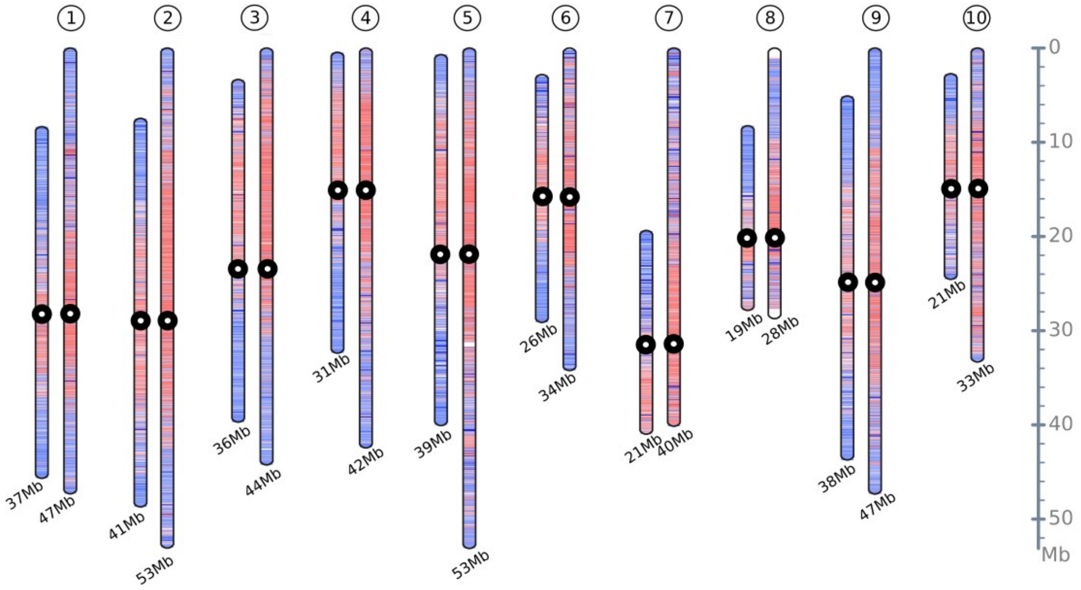

Figure 6. Comparative idiogram map between *Theobroma grandiflorum* (right) and *T. cacao* (left) showing the chromosome sizes. The idiograms illustrate gene-rich regions (blue), TE-rich regions (red), and potential location of centromeres (black circles).

Figure S1. ncRNA distribution in *Theobroma grandiflorum* chromosomes.

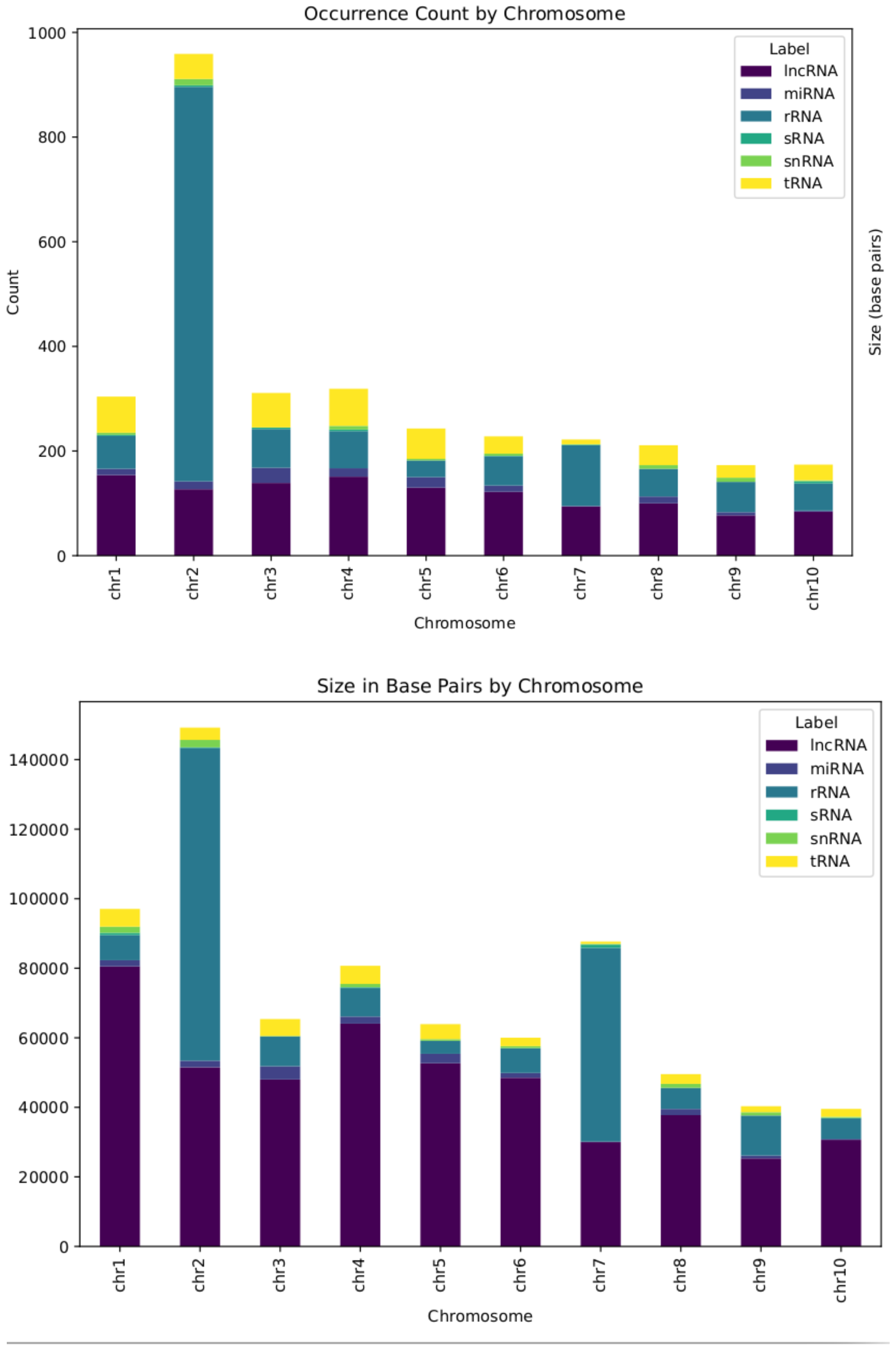

Figure S2. LTR insertion time of *Gypsy* and *Copia* elements. **A.** *Theobroma grandiflorum,* **B.** *T. cacao,* and **C.** *Herrania umbratica.* The vertical black line represents the median, and the dotted line represents the mean. The age of LTR insertions was estimated using the default substitution rate of 1.3×10^{-8} substitutions per site per year, making this calculation an approximate estimation.

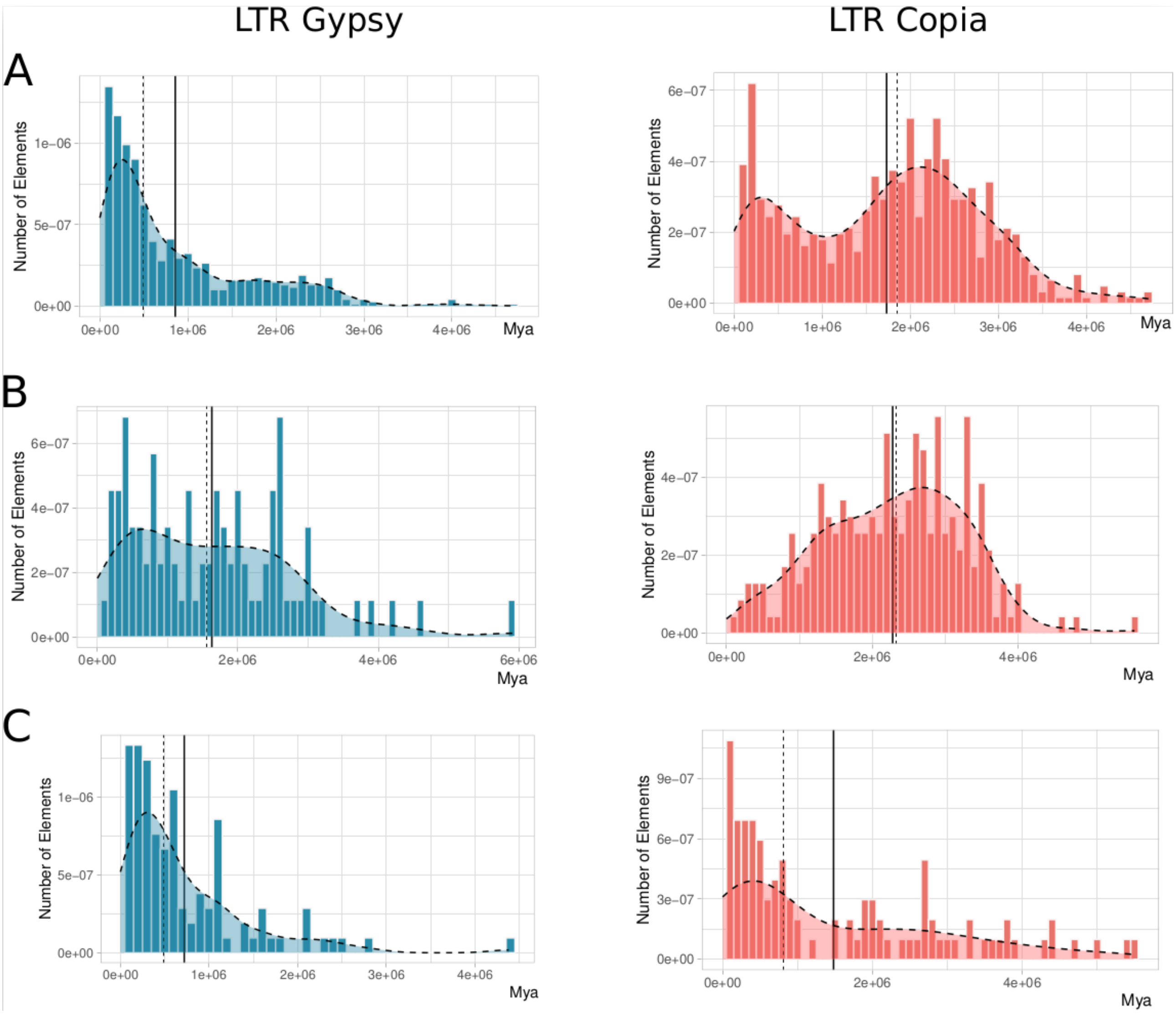

Figure S3. TE_density analyses of all *Theobroma grandiflorum* chromosomes.

Provided as external PDF file

Figure S4. A. Microsynteny and colinearity example of subtelomeric regions of *Theorboma grandiflorum*, *T. cacao* and *Herrania umbratica*, **B.** Microsynteny and colinearity example of pericentromeric regions of *T. grandiflorum*, *T. cacao* and *H. umbratica.* Blue represents genes in the forward direction, green indicates genes in the reverse direction, and orange denotes transposable elements (TEs).

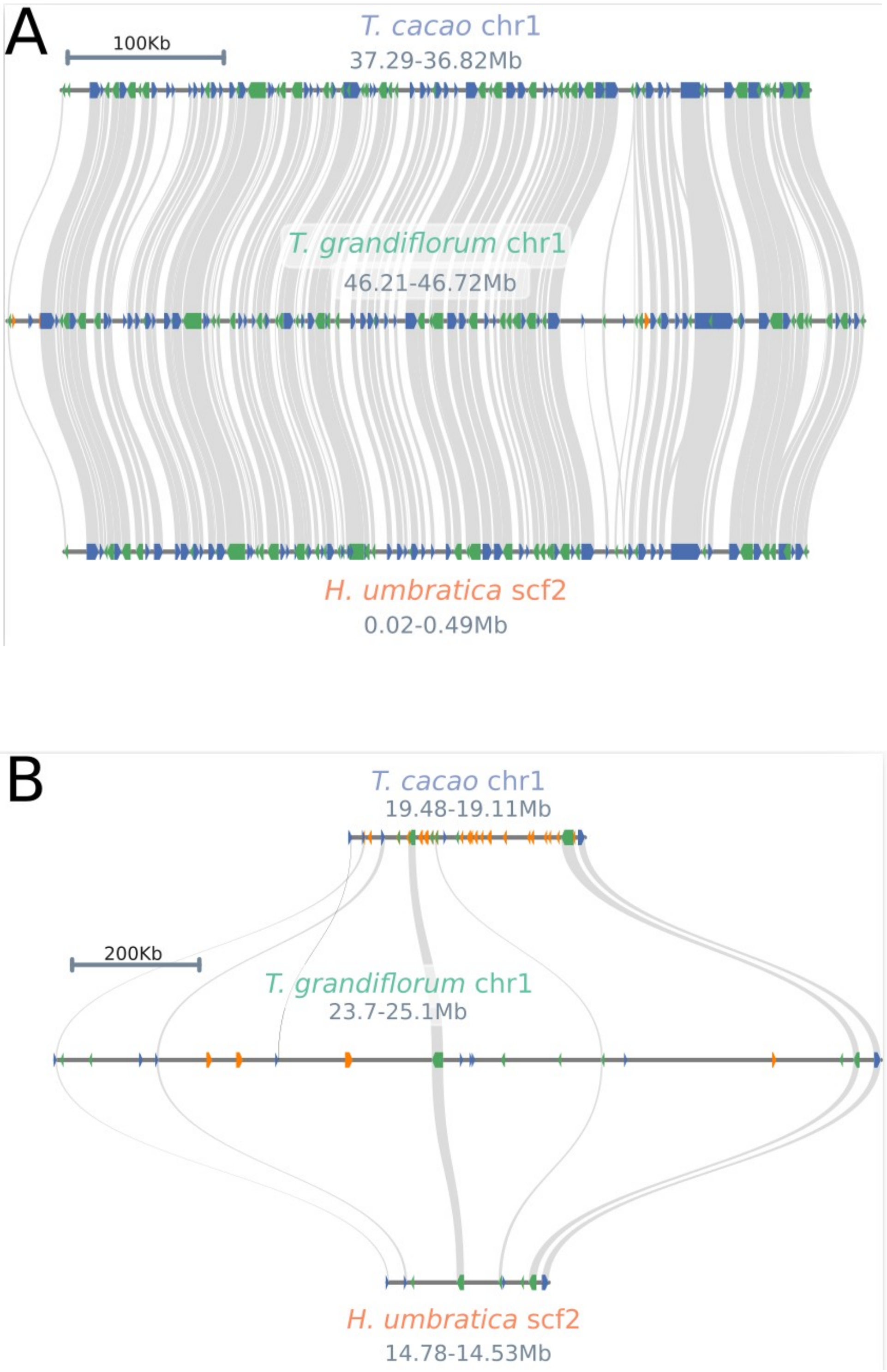

Figure S5. Alignment of the *GEX1* gene from *CH4* loci generated on Jalview (Procter et al., 2021).

Provided as external PDF file

Figure S6. Box-plot and swarmplot showing the the Ka/Ks ratio distributions of the selected GO terms associated with fruit traits and defense mechanisms. A. *Theobroma cacao*, B. *Herrania umbratica*.

Provided as external PDF file

Figure S7. Genomic mapping of plant disease resistance genes in *Theobroma grandiflorum* chromosomes. Genes under positive selection are shown in red. The cupuassu WBD-resistant QTL is shown in blue.

Provided as external PDF file

Table S1. GenBank SRA accession numbers used for transcriptome assembly. **A.** All *Theobroma cacao* RNAseq data used. **B.** *Herrania umbratica* RNAseq data used.

A

SRR7388543	SRR7388568	SRR7388593	SRR11389079	SRR7172375	SRR3217284
SRR7388544	SRR7388569	SRR7388594	SRR11389080	SRR7172376	SRR3217292
SRR7388545	SRR7388570	SRR7388595	SRR11389081	SRR7172377	SRR3217294
SRR7388546	SRR7388571	SRR7388596	SRR11389082	SRR7172378	SRR3217297
SRR7388547	SRR7388572	SRR7388597	SRR11389083	SRR7172379	SRR3217298
SRR7388548	SRR7388573	SRR7388598	SRR11389084	SRR7172380	SRR3217299
SRR7388549	SRR7388574	SRR7388599	SRR7172356	SRR7172381	SRR3217301
SRR7388550	SRR7388575	SRR7388600	SRR7172357	SRR7172382	SRR3217304
SRR7388551	SRR7388576	SRR7388601	SRR7172358	SRR7172383	SRR3217315
SRR7388552	SRR7388577	SRR7388602	SRR7172359	SRR7172384	SRR3217317
SRR7388553	SRR7388578	SRR7388603	SRR7172360	SRR7172385	SRR3217318
SRR7388554	SRR7388579	SRR7388604	SRR7172361	SRR7172386	SRR3217319
SRR7388555	SRR7388580	SRR7388605	SRR7172362	SRR7172387	SRR1034656
SRR7388556	SRR7388581	SRR7388606	SRR7172363	SRR7172388	SRR1036616
SRR7388557	SRR7388582	SRR7388607	SRR7172364	SRR7172389	SRR747762
SRR7388558	SRR7388583	SRR7388608	SRR7172365	SRR7172390	SRR747765
SRR7388559	SRR7388584	SRR7388609	SRR7172366	SRR7172391	SRR747772
SRR7388560	SRR7388585	SRR7388610	SRR7172367	SRR3217276	SRR747773
SRR7388561	SRR7388586	SRR7388611	SRR7172368	SRR3217277	SRR747774
SRR7388562	SRR7388587	SRR7388612	SRR7172369	SRR3217278	SRR747775
SRR7388563	SRR7388588	SRR7388613	SRR7172370	SRR3217279	SRR747776
SRR7388564	SRR7388589	SRR7388614	SRR7172371	SRR3217280	SRR747777
SRR7388565	SRR7388590	SRR11389076	SRR7172372	SRR3217281	SRR747778
SRR7388566	SRR7388591	SRR11389077	SRR7172373	SRR3217282	SRR747779
SRR7388567	SRR7388592	SRR11389078	SRR7172374	SRR3217283	SRR13960577
					SRR13960578
					SRR13960579

B

SRA Accession	Description
SRR5630802	open flowers
SRR5481331	open flowers
SRR5481332	lateral buds
SRR5481333	closed flower buds
SRR5481334	apical stems
SRR5462401	young leaves

Table S2. Genome assembly statistics and completeness scores of the three Theobromateae genomes (BUSCO scores were retrieved using embryophyta_odb10).

**Features**	***T. grandiflorum* C1074** (this work)	***T. cacao* Criollo B97-61/B2 v2** (re-annotation)	***H. umbratica* Fairchild** (re-assembly and re-annotation)
-Genome length	423,916,809 bp	324,761,211 bp	249,829,655 bp
-% assembled by flow cytometry	94%	71%	70%
**--- Primary Assembly**			
-------Assembly size	434,716,736	-	267,828,918
-----Number of contigs	131	-	3464
-----Longest contigs	53,228,916	-	9,298,149
-----Shortest contigs	17,612	-	208
-----Number of contigs > 1K	131	-	3,456
-----Number of contigs > 10K	131	-	1,428
-----Number of contigs > 100K	38	-	320
-----Number of contigs > 1M	14	-	60
-----Number of contigs > 10M	11	-	0
-----Mean contigs size	33,18,448	-	77,318
-----Median contigs size	46,682	-	6,769
-----N50 contigs length	42,442,329	-	1,047,534
-----L50 contigs count	6	-	55
**--- HiC Assembly / GenBank**			
--- Chromosomes / Scaffolds	10	10 + 1 unplaced	621
----- N50/L50	44 Mb / 5	36 Mb / 5	13 Mb / 8
-BUSCO genome	C:98.4% [S:97.5%, D:0.9%], F:0.9%, M:0.7%, n:1614	99.3% [S:98.3%, D:1.0%], F:0.1%, M:0.6%, n:1614	C:98.3% [S:93.2%, D:5.1%], F:0.8%, M:0.9%, n:1614
-BUSCO annotation	C:99.8% [S:59.5%, D:40.3%], F:0.1%, M:0.1%, n:1614	C:99.4% [S:68.2%, D:31.2%], F:0.3%, M:0.3%, n:1614	C:99.1% [S:58.0%, D:41.1%], F:0.5%, M:0.4%, n:1614
-LAI	15.66	9.53	14.02

Table S3 Summary of gaps on the *T. grandiflorum* chromosomes (the genomic coordinates includes ~500bp boundaries).

Gap ID	Chromosome	Start (bp)	End (bp)	Location on chromosome	Characteristics
gap1	chr5	31,826,851	31,828,000	centromeric region	LTR/LARD
gap2	chr6	3,261,901	3,263,050	distal	protein E6-like
gap3	chr8	978,651	979,800	distal	Telomeric repeat

Table S4. Summary of telomeres on the *T. grandiflorum* chromosomes.

Chromosome	Start (bp)	End (bp)	Motif
chr1	7	1,659	(AAACCCT)$_{226}$
chr2	7	9,219	(AAACCCT)$_{1314}$
chr2	53,067,014	53,068,316	(GTTTAGG)$_{145}$
chr3	7	6,363	(AAACCCT)$_{882}$
chr3	44,240,161	44,241,190	(TCAGGGT)$_{133}$
chr4	7	5,586	(ACCCTAA)$_{794}$
chr4	42,432,306	42,434,182	(GGGTTCA)$_{228}$
chr5	7	12,005	(ACCCTAA)$_{1573}$
chr5	53,103,288	53,105,031	(AGGGTTT)$_{246}$
chr6	7	11,697	(CCCTAAA)$_{246}$
chr6	34,212,801	34,216,574	(GGTTTAG)$_{458}$
chr7	40,136,684	40,139,869	(GTTTAGG)$_{452}$
chr8	21	5,124	(CTAAACC)$_{643}$
chr9	7	10,115	(AACCCTA)$_{1436}$
chr9	47,322,555	47,326,986	(GGGTTCA)$_{278}$
chr10	7	20,566	(ACCCTAA)$_{2915}$
chr10	33305034	33316129	(ACCCTAA)$_{1199}$

Table S5. Summary of centromeres on the *T. grandiflorum* chromosomes.

Chromosome	Start (bp)	End (bp)	Size (bp)
chr1	18,880,000	19,600,000	720,000
chr2	28,710,000	29,220,000	510,000
chr3	23,130,000	24,020,000	890,000
chr4	27,170,000	27,620,000	450,000
chr5	31,750,000	32,100,000	350,000
chr6	18,290,000	18,470,000	180,000
chr7	8,850,000	9,310,000	460,000
chr8	8,880,000	9,350,000	470,000
chr9	22,210,000	23,160,000	950,000
chr10	17,690,000	18,820,000	1,130,000

Table S6. Genome annotation features and statistics of the three Theobromateae genomes.

**Features**	***T. grandiflorum* C1074** (this work)	***T. cacao* Criollo B97-61/B2 v2** (re-annotation)	***H. umbratica* Fairchild** (re-assembly and re-annotation)
-Number of genes	31,381	30,623	27905
-- Number of exons	285,619	271540	314599
-- Number of introns	238,948	223339	264443
-- Number of CDSs	46,671	48201	50156
---- complete CDS	46,625	46872	50049
---- start, no stop CDS	8	387	46
---- stop, no start CDS	22	453	46
---- no stop, no start CDS	16	489	15
-- Overlapping genes	3233	5965	4795
-- Contained genes	892	2178	1374
-Shortest gene	153 bp	150 bp	153 bp
-Longest gene	227,387 bp	143,212 bp	121,875 bp
-Longest CDS	16,371 bp	16,368 bp	16,374 bp
-mean gene length	3,374 bp	3,521 bp	3,642 bp
-mean CDS length	1,331 bp	1,214 bp	1,332 bp
-mean exons per gene	6	5	6
-mean introns per gene	5	4	5
-WGD gene pairs	4,996	4,875	8,505
-Tandem gene pairs	3,667	3,476	3,146
-Proximal duplicated gene pairs	3,551	2,562	1,960
-Dispersed duplicated gene pairs	14.567	14,222	9,773
-Singletons	4,600	5,488	4,521
-Transposed gene pairs	2,367	2,703	1,204
-Retrocopies	402	410	309
-- Parental Gene	214	191	169
---- Chimerical	197	215	164
---- Pseudogene	37	40	37
---- Retrogene	168	155	108
% of genome covered by genes	25%	33.2%	40.7%
% of genome covered by CDS	14.70%	18%	26.7%
% of genome covered by TEs	63.37%	44.51%	35.21%
% of Class I Elements	52.84%	35.41%	14.24%
% of LTR Gypsy	15.40%	8.72%	2.85%
% of LTR Copia	21.43%	16.78%	3.77%
% of LTR non-autonomous	14.86%	9.03%	6.78%

% of Class II Elements	2.75%	3.48%	3.02%
% TIRs	1.40%	1.08%	1.34%
% Helitron	1.36%	2.39%	1.67%

Table S7. Retrocopies identified in *Theobroma grandiflorum*, *T. cacao*, and *Herrania umbratica,* with associated raw data.

Provided as external Excel file

Table S8. Genome structural features and statistics for each *Theobroma grandiflorum* chromosome.

Theobroma grandiflorum Genome Features	**chr1**	**chr2**	**chr3**	**chr4**	**chr5**	**chr6**	**chr7**	**chr8**	**chr9**	**chr10**
- Synteny against *T.cacao* (% mean (SD) and % median)	71.05 (35) 86.67	62.02 (40) 76.92	61.15 (42) 80.00	61.42 (39) 75.00	61.24 (40) 76.92	67.57 (37) 83.33	63.18 (38) 75.00	68.95 (37) 84.62	69.38 (36) 85.71	61.76 (38) 73.33
- Synteny against *H. umbratica* (% mean (SD) and % median)	66.93 (36) 81.82	53.61 (40) 62.50	57.01 (42) 71.43	58.21 (40) 71.43	56.93 (40) 67.54	61.87 (38) 75.00	53.11 (40) 63.64	62.01 (38) 75.00	65.27 (37) 80.00	57.62 (38) 66.67
-Genes (count)	4,199	3,764	3,258	3303	3544	2550	2824	2044	3624	2271
-Genes (% bp occupied)	24.28	17.46	19.08	20.41	18.51	21.92	19.87	20.43	21.20	18.18
-tRNAs (count)	74	48	69	75	63	35	9	40	24	34
-snoRNA (count)	144	138	120	116	107	92	85	100	92	64
-miRNA (count)	12	15	29	16	20	12	1	13	6	2
-sRNA (count)	2	3	3	4	-	2	1	-	-	4
-lncRNA (count)	154	127	139	151	130	122	94	100	77	84
-RNA										
--5S (coordinates)		21,423,309 26,917,293								
--45S (coordinates)							4,890 504,355			
-TEs (count)	41799	52670	43272	40554	56794	32476	44759	25008	44134	35774
-TEs (% bp occupied)	48.88	59.59	57.41	56.28	60.35	55.73	61.46	57.03	51.92	59.27
-LTR Copia (count)	8957	12730	10116	9436	12724	7505	9079	5280	9732	7388
-LTR Copia (% bp occupied)	16.64	21.66	20.40	19.33	20.63	19.62	18.36	16.27	17.99	18.45
-LTR Gypsy (count)	7237	10507	8859	7713	10609	6150	7940	4420	7945	6704
-LTR Gypsy (% bp occupied)	10.89	14.39	14.47	13.27	14.19	13.55	15.09	10.77	11.99	14.90
-LARD (count)	4818	6398	5297	5015	7665	3879	6344	3190	4636	4631
-LARD (% bp occupied)	6.04	7.42	7.01	7.36	7.63	6.94	9.60	7.15	6.09	8.44
-TRIM (count)	227	252	188	240	245	166	220	138	244	202
-TRIM (% bp occupied)	0.17	0.17	0.12	0.17	0.16	0.15	0.23	0.16	0.17	0.18
-BARE-2 (count)	572	651	512	539	690	445	450	321	595	352
-BARE-2 (% bp occupied)	1.00	1.12	1.02	1.19	1.07	0.93	0.93	0.82	1.02	1.06
-TR_GAG (count)	2110	3254	2680	2376	3327	1889	2356	1260	2346	1966
-TR_GAG (% bp occupied)	3.62	4.81	5.07	4.73	5.25	4.38	4.48	3.42	3.74	4.51
-LINE (count)	595	708	544	539	873	528	567	377	673	519
-LINE (% bp occupied)	0.82	0.97	0.73	0.90	1.08	0.98	0.84	0.84	0.93	1.00
-SINE (count)	36	44	31	44	48	22	19	24	36	20

-SINE (% bp occupied)	0.01	0.01	0.01	0.01	0.01	0.01	0.001	0.01	0.01	0.001
-pararetrovirus (count)	12	36	13	12	30	9	18	34	26	7
-pararetrovirus (% bp occupied)	0.02	0.10	0.06	0.04	0.08	0.03	0.08	0.18	0.09	0.06
-TIR (count)	941	929	678	780	877	577	681	498	902	618
-TIR (% bp occupied)	1.05	1.05	0.91	1.15	0.97	0.93	1.00	1.03	1.15	1.20
-Helitron (count)	3074	2868	2330	2333	3267	2110	2174	1595	3008	1863
-Helitron (% bp occupied)	1.42	1.20	1.13	1.25	1.25	1.45	1.24	1.18	1.37	1.32
-MITE (count)	812	810	664	721	854	531	692	423	796	573
-MITE (% bp occupied)	0.30	0.26	0.26	0.26	0.34	0.25	0.26	0.25	0.29	0.28
-Unknown (count)	12407	13483	11357	10805	15582	8664	14217	7448	13195	10930
-Unknown (% bp occupied)	6.92	6.42	6.23	6.63	7.71	6.52	9.36	14.95	7.09	7.88

Table S9. Transposable elements summary table and statistics identified of the three Theobromateae genomes

Provided as external Excel file

Table S10. Exclusive gene families identified for each Theobromateae genome analyzed.

Provided as external Excel file

Table S11. Singletons identified in each Theobromateae genome analyzed.

Provided as external Excel file

Table S12. Expanded and contracted gene families identified in each Theobromateae genome analyzed.

Provided as external Excel file

Table S13. GO enrichment analyses raw data.

Provided as external Excel file

Table S14. Genes and GO terms identified as positively selected by Ka/Ks analysis.

Provided as external Excel file

Table S15. Gene content and features of cupuassu WBD-resistant QTL.

Provided as external Excel file

462 **References**

1. Procter JB, Carstairs GM, Soares B, Mourão K, Ofoegbu TC, Barton D, et al.. Alignment of Biological Sequences with Jalview. *Methods Mol Biol*. 2021; doi: 10.1007/978-1-0716-1036-7_13.

2. Haas BJ, Delcher AL, Mount SM, Wortman JR, Smith RK, Hannick LI, et al.. Improving the Arabidopsis genome annotation using maximal transcript alignment assemblies. *Nucleic Acids Res*. 2003; doi: 10.1093/nar/gkg770.

3. Ou S, Su W, Liao Y, Chougule K, Agda JRA, Hellinga AJ, et al.. Benchmarking transposable element annotation methods for creation of a streamlined, comprehensive pipeline. *Genome Biol*. 2019; doi: 10.1186/s13059-019-1905-y.

4. Ou S, Chen J, Jiang N. Assessing genome assembly quality using the LTR Assembly Index (LAI). *Nucleic Acids Res*. 2018; doi: 10.1093/nar/gky730.

5. Li Y, Jiang N, Sun Y. AnnoSINE: a short interspersed nuclear elements annotation tool for plant genomes. *Plant Physiol*. 2022; doi: 10.1093/plphys/kiab524.

6. Rho M, Tang H. MGEScan-non-LTR: computational identification and classification of autonomous non-LTR retrotransposons in eukaryotic genomes. *Nucleic Acids Res*. 2009; doi: 10.1093/nar/gkp752.

7. Orozco-Arias S, Isaza G, Guyot R. Retrotransposons in Plant Genomes: Structure, Identification, and Classification through Bioinformatics and Machine Learning. *Int J Mol Sci*. 2019; doi: 10.3390/ijms20153837.

8. Zhang R-G, Li G-Y, Wang X-L, Dainat J, Wang Z-X, Ou S, et al.. TEsorter: an accurate and fast method to classify LTR-retrotransposons in plant genomes. *Hortic Res*. 2022; doi: 10.1093/hr/uhac017.

9. Mokhtar MM, Alsamman AM, El Allali A. PlantLTRdb: An interactive database for 195 plant species LTR-retrotransposons. *Front Plant Sci*. undefined; 2023; doi: 10.3389/fpls.2023.1134627.

10. Minh BQ, Schmidt HA, Chernomor O, Schrempf D, Woodhams MD, von Haeseler A, et al.. IQ-TREE 2: New Models and Efficient Methods for Phylogenetic Inference in the Genomic Era. *Mol Biol Evol*. 2020; doi: 10.1093/molbev/msaa015.

11. Hoff KJ, Lomsadze A, Borodovsky M, Stanke M. Whole-Genome Annotation with BRAKER. *Methods Mol Biol*. 2019; doi: 10.1007/978-1-4939-9173-0_5.

12. Gabriel L, Brůna T, Hoff KJ, Ebel M, Lomsadze A, Borodovsky M, et al.. BRAKER3: Fully automated genome annotation using RNA-Seq and protein evidence with GeneMark-ETP, AUGUSTUS and TSEBRA. *bioRxiv*. 2023; doi: 10.1101/2023.06.10.544449.

13. Gabriel L, Hoff KJ, Brůna T, Borodovsky M, Stanke M. TSEBRA: transcript selector for BRAKER. *BMC Bioinformatics*. 2021; doi: 10.1186/s12859-021-04482-0.

14. Slater GSC, Birney E. Automated generation of heuristics for biological sequence comparison. *BMC Bioinformatics*. 2005; doi: 10.1186/1471-2105-6-31.

15. Brůna T, Li H, Guhlin J, Honsel D, Herbold S, Stanke M, et al.. Galba: genome annotation with miniprot and AUGUSTUS. *BMC Bioinformatics*. 2023; doi: 10.1186/s12859-023-05449-z.

16. Li H. Protein-to-genome alignment with miniprot. *Bioinformatics*. 2023; doi: 10.1093/bioinformatics/btad014.

17. Keilwagen J, Hartung F, Grau J. GeMoMa: Homology-Based Gene Prediction Utilizing Intron Position Conservation and RNA-seq Data. *Methods Mol Biol*. 2019; doi: 10.1007/978-1-4939-9173-0_9.

18. Manni M, Berkeley MR, Seppey M, Zdobnov EM. BUSCO: Assessing Genomic Data Quality and Beyond. *Curr Protoc*. 2021; doi: 10.1002/cpz1.323.

19. Haas BJ, Salzberg SL, Zhu W, Pertea M, Allen JE, Orvis J, et al.. Automated eukaryotic gene structure annotation using EVidenceModeler and the Program to Assemble Spliced Alignments. *Genome Biology*. 2008; doi: 10.1186/gb-2008-9-1-r7.

20. Teufel F, Almagro Armenteros JJ, Johansen AR, Gíslason MH, Pihl SI, Tsirigos KD, et al.. SignalP 6.0 predicts all five types of signal peptides using protein language models. *Nat Biotechnol*. 2022; doi: 10.1038/s41587-021-01156-3.

21. Käll L, Krogh A, Sonnhammer ELL. A combined transmembrane topology and signal peptide prediction method. *J Mol Biol*. 2004; doi: 10.1016/j.jmb.2004.03.016.

22. Camacho C, Coulouris G, Avagyan V, Ma N, Papadopoulos J, Bealer K, et al.. BLAST+: architecture and applications. *BMC Bioinformatics*. 2009; doi: 10.1186/1471-2105-10-421.

23. UniProt Consortium. UniProt: the Universal Protein Knowledgebase in 2023. *Nucleic Acids Res*. 2023; doi: 10.1093/nar/gkac1052.

24. O'Leary NA, Wright MW, Brister JR, Ciufo S, Haddad D, McVeigh R, et al.. Reference sequence (RefSeq) database at NCBI: current status, taxonomic expansion, and functional annotation. *Nucleic Acids Res*. 2016; doi: 10.1093/nar/gkv1189.

25. Wang Y, Tang H, Debarry JD, Tan X, Li J, Wang X, et al.. MCScanX: a toolkit for detection and evolutionary analysis of gene synteny and collinearity. *Nucleic Acids Res*. 2012; doi: 10.1093/nar/gkr1293.

26. Wei Z, Sun J, Li Q, Yao T, Zeng H, Wang Y. RetroScan: An Easy-to-Use Pipeline for Retrocopy Annotation and Visualization. *Frontiers in Genetics*. 122021;

27. Qiao X, Li Q, Yin H, Qi K, Li L, Wang R, et al.. Gene duplication and evolution in recurring polyploidization–diploidization cycles in plants. *Genome Biology*. 2019; doi: 10.1186/s13059-019-1650-2.

28. Flagel LE, Wendel JF. Gene duplication and evolutionary novelty in plants. *New Phytol*. 2009; doi: 10.1111/j.1469-8137.2009.02923.x.

29. Jin J, Tian F, Yang D-C, Meng Y-Q, Kong L, Luo J, et al.. PlantTFDB 4.0: toward a central hub for transcription factors and regulatory interactions in plants. *Nucleic Acids Res*. 2017; doi: 10.1093/nar/gkw982.

30. Martin EC, Ion CF, Ifrimescu F, Spiridon L, Bakker J, Goverse A, et al.. NLRscape: an atlas of plant NLR proteins. *Nucleic Acids Res*. 2023; doi: 10.1093/nar/gkac1014.

31. Calle García J, Guadagno A, Paytuvi-Gallart A, Saera-Vila A, Amoroso CG, D'Esposito D, et al.. PRGdb 4.0: an updated database dedicated to genes involved in plant disease resistance process. *Nucleic Acids Res*. 2022; doi: 10.1093/nar/gkab1087.

32. Huerta-Cepas J, Szklarczyk D, Heller D, Hernández-Plaza A, Forslund SK, Cook H, et al.. eggNOG 5.0: a hierarchical, functionally and phylogenetically annotated orthology resource based on 5090 organisms and 2502 viruses. *Nucleic Acids Res*. 2019; doi: 10.1093/nar/gky1085.

33. Mulder N, Apweiler R. InterPro and InterProScan: tools for protein sequence classification and comparison. *Methods Mol Biol*. 396:59–702007;

34. Kautsar SA, Suarez Duran HG, Medema MH. Genomic Identification and Analysis of Specialized Metabolite Biosynthetic Gene Clusters in Plants Using PlantiSMASH. *Methods Mol Biol*. 2018; doi: 10.1007/978-1-4939-7874-8_15.

35. Conesa A, Götz S, García-Gómez JM, Terol J, Talón M, Robles M. Blast2GO: a universal tool for annotation, visualization and analysis in functional genomics research. *Bioinformatics*. 2005; doi: 10.1093/bioinformatics/bti610.

36. RNAcentral Consortium. RNAcentral 2021: secondary structure integration, improved sequence search and new member databases. *Nucleic Acids Res*. 2021; doi: 10.1093/nar/gkaa921.

37. Nawrocki EP, Eddy SR. Infernal 1.1: 100-fold faster RNA homology searches. *Bioinformatics*. 2013; doi: 10.1093/bioinformatics/btt509.

38. Kalvari I, Nawrocki EP, Ontiveros-Palacios N, Argasinska J, Lamkiewicz K, Marz M, et al.. Rfam 14: expanded coverage of metagenomic, viral and microRNA families. *Nucleic Acids Res*. 2021; doi: 10.1093/nar/gkaa1047.

39. Diesh C, Stevens GJ, Xie P, De Jesus Martinez T, Hershberg EA, Leung A, et al.. JBrowse 2: a modular genome browser with views of synteny and structural variation. *Genome Biol*. 2023; doi: 10.1186/s13059-023-02914-z.

40. Argout X, Martin G, Droc G, Fouet O, Labadie K, Rivals E, et al.. The cacao Criollo genome v2.0: an improved version of the genome for genetic and functional genomic studies. *BMC Genomics*. 2017; doi: 10.1186/s12864-017-4120-9.

41. Argout X, Salse J, Aury J-M, Guiltinan MJ, Droc G, Gouzy J, et al.. The genome of Theobroma cacao. *Nat Genet*. 2011; doi: 10.1038/ng.736.

42. da Silva RA, Souza G, Lemos LSL, Lopes UV, Patrocínio NGRB, Alves RM, et al.. Genome size, cytogenetic data and transferability of EST-SSRs markers in wild and cultivated species of the genus Theobroma L. (Byttnerioideae, Malvaceae). *PLoS One*. 2017; doi: 10.1371/journal.pone.0170799.

43. Dantas LG, Guerra M. Chromatin differentiation between Theobroma cacao L. and T. grandiflorum Schum. *Genet Mol Biol*. 2010; doi: 10.1590/S1415-47572009005000103.

44. Belser C, Baurens F-C, Noel B, Martin G, Cruaud C, Istace B, et al.. Telomere-to-telomere gapless chromosomes of banana using nanopore sequencing. *Commun Biol*. 2021; doi: 10.1038/s42003-021-02559-3.
