# Supplementary figures and images for: Genomic decoding of Theobroma grandiflorum (cupuassu) at chromosomal scale: evolutionary insights for horticultural innovation
Source: Gigascience. 2024 Jun 5;13:giae027. doi: 10.1093/gigascience/giae027 (PMC11152179; doi:10.1093/gigascience/giae027)

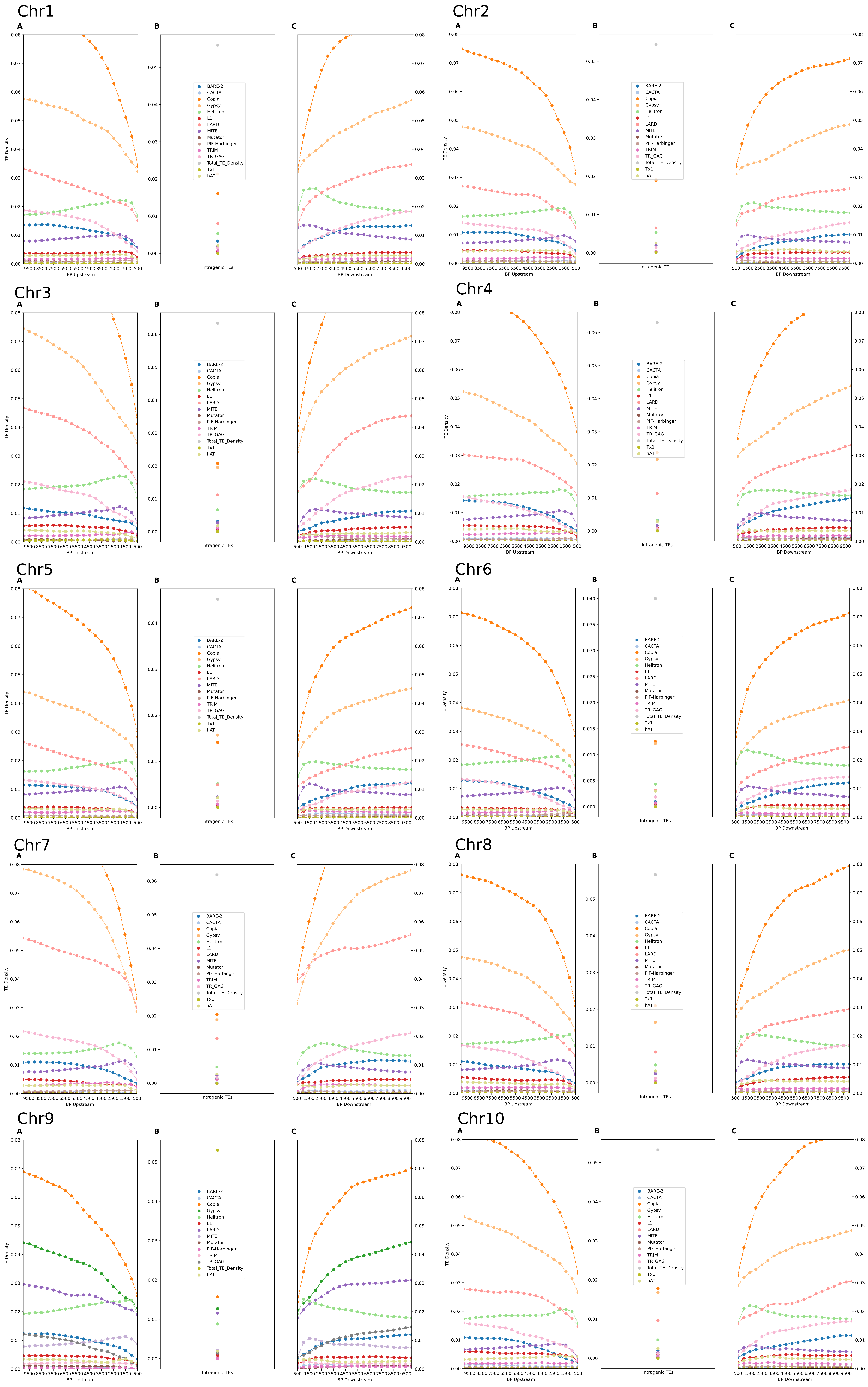

Supplement: giae027_supplement [file giae027_supplement.zip › FigureS3.pdf]

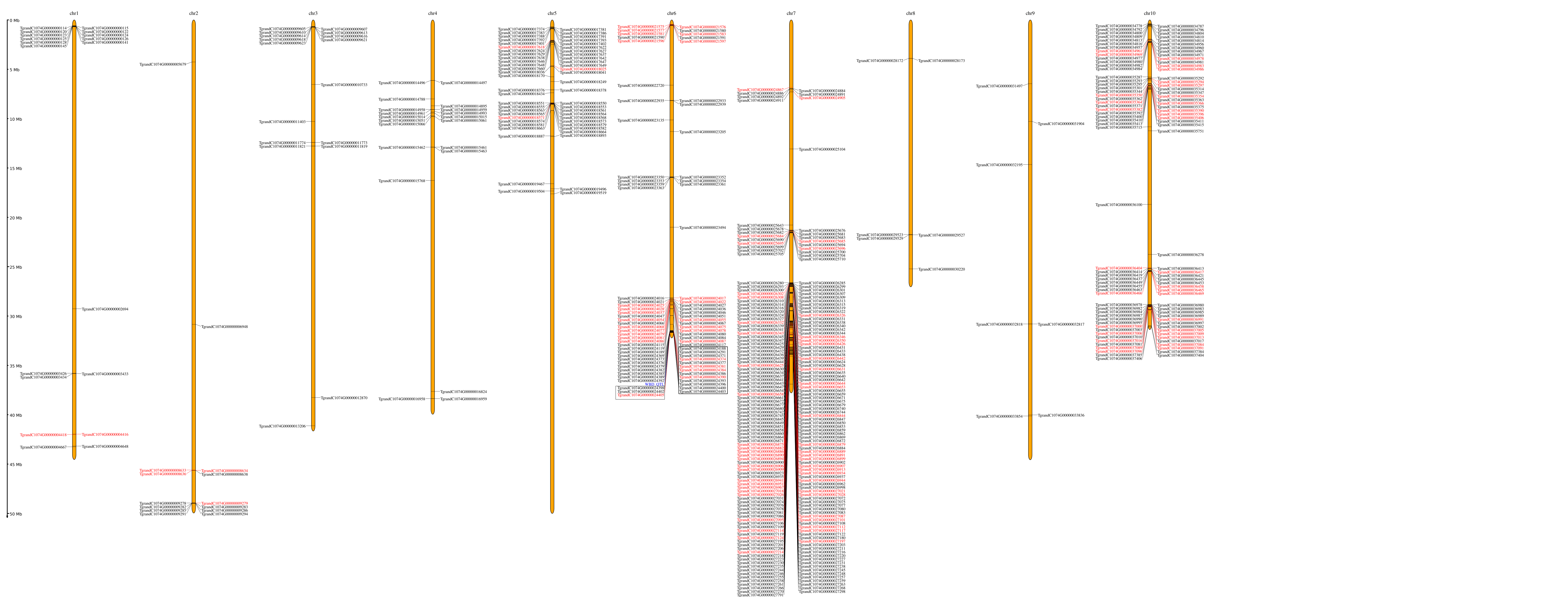

Supplement: giae027_supplement [file giae027_supplement.zip › FigureS7.pdf]
