# Supplementary material for: Genomic decoding of Theobroma grandiflorum (cupuassu) at chromosomal scale: evolutionary insights for horticultural innovation
Source: Gigascience. 2024 Jun 5;13:giae027. doi: 10.1093/gigascience/giae027 (PMC11152179; doi:10.1093/gigascience/giae027)
Supplement: giae027_GIGA_D_23_00404_Revision_1 [file giae027_giga_d_23_00404_revision_1.pdf]

GigaScience

Genomic decoding of Theobroma grandiflorum (cupuassu) at chromosomal scale: Evolutionary insights for horticultural innovation

--Manuscript Draft--

**Manuscript Number:**	GIGA-D-23-00404R1
**Full Title:**	Genomic decoding of Theobroma grandiflorum (cupuassu) at chromosomal scale: Evolutionary insights for horticultural innovation
**Article Type:**	Data Note
**Funding Information:**	

Funder	Recipient
Fundação de Amparo à Pesquisa do Estado de São Paulo (2019/25176-0)	Dr Alessandro M. Varani
Fundação Amazônia Paraense de Amparo à Pesquisa (075/2020)	Dr Rafael Moysés Alves<br>Dr Vinicius A. C. de Abreu
Fundação Araucária (66.2021)	Dr Alexandre R. Paschoal
Conselho Nacional de Desenvolvimento Científico e Tecnológico (304367/2022-2)	Dr Alessandro M. Varani
Conselho Nacional de Desenvolvimento Científico e Tecnológico (313174/2022)	Dr Douglas S. Domingues

Abstract:

Background

Theobroma grandiflorum (Malvaceae), known as cupuassu, is a tree indigenous to the Amazon Basin, valued for its large fruits and seed-pulp, contributing notably to the Amazonian bioeconomy. The seed-pulp is utilized in desserts and beverages, and its seed butter is used in cosmetics. Here, we present the sequenced telomere-to-telomere cupuassu genome, disclosing features of the genomic structure, evolution, and phylogenetic relationships within the Malvaceae.

Findings

The cupuassu genome spans 423 Mb, encodes 31,381 genes distributed in the ten chromosomes, and it exhibits approximately 65% gene synteny with the T. cacao genome, reflecting a conserved evolutionary history, albeit punctuated with unique genomic variations. The main changes are pronounced by bursts of long-terminal repeats retrotransposons expansion at postspecies divergence, retrocopied and singleton genes, and gene families displaying distinctive patterns of expansion and contraction. Furthermore, positively selected genes are evident, particularly among retained and dispersed, tandem and proximal duplicated genes associated to general fruit and seed traits and defense mechanisms, supporting the hypothesis of potential episodes of subfunctionalization and neofunctionalization following duplication, and impact from distinct domestication process. These genomic variations may underpin the differences observed in fruit and seed morphology, ripening, and disease resistance between cupuassu and the other Malvaceae species.

Conclusions

Sequencing the cupuassu genome offers a foundational resource for both breeding and conservation efforts, yielding insights into the evolution and diversity within the genus Theobroma.

**Corresponding Author:**	Alessandro M. Varani, PhD<br>UNESP Campus de Jaboticabal: Universidade Estadual Paulista Julio de Mesquita Filho - Campus de Jaboticabal<br>Jaboticabal, Sao Paulo BRAZIL
**Corresponding Author Secondary Information:**	

Powered by Editorial Manager® and ProduXion Manager® from Aries Systems Corporation

Corresponding Author's Institution:	UNESP Campus de Jaboticabal: Universidade Estadual Paulista Julio de Mesquita Filho - Campus de Jaboticabal
Corresponding Author's Secondary Institution:	
First Author:	Rafael Moysés Alves
First Author Secondary Information:	
Order of Authors:	Rafael Moysés Alves
	Vinicius A. C. de Abreu
	Rafaely Pantoja Oliveira
	João Victor dos Anjos Almeida
	Mauro de Medeiros de Oliveira
	Saura R. Silva
	Alexandre R. Paschoal
	Sintia S. de Almeida
	Pedro A.F. de Souza
	Jesus A. Ferro
	Vitor F.O. Miranda
	Douglas S. Domingues
	Antonio Figueira
	Alessandro M. Varani
Order of Authors Secondary Information:	
Response to Reviewers:	Rebuttal letter<br><br>Editor comment<br><br>Please also note, after assessing the paper with the reviewers, we feel the main advance of the paper is the presentation of a genome assembly and some more basic analyses, rather than answering a specific biological research question. We will therefore consider the revised manuscript for publication in our "Data Note" section. Please submit the revised paper as "Data Note" and change the format accordingly (see the instructions for authors on our homepage). You do not need to shorten the article, but the order and titles of sections are slightly different in a Data Note.<br><br>In response to your guidance and after careful consideration of the reviewers' assessments, we have revised our manuscript as suggested. We have formatted our submission with the "Data Note" requirements, adhering to the specific order and titles of sections as outlined in the instructions for authors. We appreciate this opportunity to present our manuscript on genome assembly and analyses within the framework of a "Data Note."<br>Please note that all modifications are highlighted with a light gray background or indicated by track changes.<br><br>Reviewer reports:<br><br>Reviewer #1<br><br>Reviewer #1: 1. The Line or page number should be added in the revised manuscript, it is hard to point the comment to definite line.<br><br>We apologize for the inconvenience. We have now included line and page numbers in

	this revised version of the manuscript.<br><br>2. The methods and parameters of TE analysis should be detailed in the main text or supplementary file, especially for the LAI calculation, the LAI output by our pipeline is 11.47 and the pipeline was built according to default parameters of LTR_retiever (https://github.com/oushujun/LTR_retriever).<br><br>Our transposable element (TE) pipeline is based on the EDTA version with AnnoSINE and MGEScan-non-LTR software for SINE and LINE detections, respectively. It also uses TEsorter for element validation and classification. This pipeline is described in Supplementary Information 1. We have also provided instructions for running this pipeline and replicating our results in our GitHub repository (https://github.com/amvarani/Plant_Annotation_TEs).<br>This pipeline employs adjusted parameters designed to identify as many LTR elements as possible, including non-autonomous elements such as LARDs, TRIMs, and others considered important evolutionary features in plant genomes.<br>One initial consideration is that the LTR motifs may become degenerated through mutations depending on the age of the element. Therefore, we used two distinct approaches with ltrharvest:<br>• In the first approach, we used the common TG...CA motif, which is already implemented in EDTA. The command line for Round #1, considering the TGCA motif, was: -minlenltr 100 -maxlenltr 7000 -maxdistltr 20000 -mintsd 4 -maxtsd 6 -motif TGCA -motifmis 1 -similar 85 -vic 10 -seed 20 -seqids yes<br>• For Round #2, not considering the TGCA motif (non-canonical motifs), the command line was: -minlenltr 100 -maxlenltr 7000 -maxdistltr 20000 -mintsd 4 -maxtsd 6 -similar 85 -vic 10 -seed 20 -seqids yes<br>For ltr_finder, we accommodated the same parameters: -w 2 -C -D 20000 -d 1000 -L 7000 -l 100 -p 20 -M 0.85<br>In LTR_retriever, we used the flag -nonTGCA for non-canonical motifs identified by ltrharvest.<br>Please note that the maximum distance between LTRs was set to 20,000 bp, which is 5 kb longer than the values used in EDTA.<br>Additionally, our pipeline implements TEsorter to classify all identified LTR elements into Copia and Gypsy superfamilies and lineages, using TEsorter's default parameters with the flags -db rexdb-plant and --hmm-database rexdb-plant, and applying the 80-80-80 (identity-coverage-length) rule.<br>Using this approach, we are able to determine the completeness and intactness of LTR elements, to classify them into lineages, and to identify potential non-autonomous elements such as TR_GAG, BARE-2, LARD, and TRIMs according to PlantLTRdb (https://doi.org/10.3389/fpls.2023.1134627, please see figure 1).<br>Regarding the LAI calculation, the reviewer is correct about their verification of the LAI score using default parameters. However, the differences noted here may be related to modifications implemented in our pipeline. According to the LAI manuscript (Ou et al., 2018), long internal regions of LTR elements and high-quality assemblies may positively impact the LAI score. Therefore, we believe that the parameter of the maximum distance between LTRs, set here to 20,000 (with 15,000 being the default), combined with the higher genome coverage and quality of the assembly (please see the responses to Reviewer #2), could be the reason for our slightly higher LAI score reported in the manuscript.<br>For example, we have calculated the internal regions of LTR elements using our pipeline and identified at least 20 intact LTR elements with long internal regions spanning more than 15,000 bp. Collectively, these elements account for up to 12,850 occurrences, spanning 14,686,032 bp, and representing 3.46% of the T. grandiflorum genome. Thus, a considerable fraction of the genome is now considered for LAI calculation, which may impact its results.<br>In fact, an LAI score between 10 and 20 is sufficient to consider a genome of reference quality, and T. grandiflorum exhibits an LAI pattern between 10 and 20, thus it can be considered well-assembled and of high quality.<br>We have explained in Supplementary Information 1 the parameters used in our pipeline and their potential impact on the LAI score.<br><br>3. What was the mutation rate (r) used for TE insert time calculation? If the insertion

	time were from the original files of EDTA, please notice that the default r is 1.3e-8 of grass family once --u was not set with promoting EDTA, that should be converted with the correct r value.<br><br>To the best of our knowledge, there is no average substitution rate available for the Theobroma genus, and the generic molecular clock is not suitable for this kind of calculation. Therefore, we decided to use the default value for the mutation rate (1.3e-8), which is commonly used for grasses and assumes at least a 2-fold higher mutation rate in TEs than in coding regions. We understand that our genus is quite distant from grasses.<br>Upon reviewing the literature, we found a recent study describing a plant LTR database, PlantLTRdb (see Mokthar et al., 2023; https://doi.org/10.3389/fpls.2023.1134627). Since an average substitution rate is not available for many plants, Mokthar et al. (2023) implemented a substitution rate of 1.5e-8 for plants other than grasses. This substitution rate of 1.5e-8 was based on previous studies, including LTR dating in Arabidopsis, flax (Malpighiales), and Eucalyptus (for more details, please see the “2.2 LTR-RT identification and classification” section of PlantLTRdb).<br>Although the difference between these rates is minimal (2e-9), we recalculated the age of the T. grandiflorum LTR elements using the 1.5e-8 rate for verification and validation purposes. We observed a reduction of approximately 15%, indicating that the age estimated using this rate are slightly younger. This minor adjustment does not significantly affect the overall conclusions of our results.<br>Given the lack of a specific substitution rate for Theobroma, we have chosen to retain the default mutation rate used in EDTA (1.3e-8), considering this an area for future research.<br>We have detailed this approach in the supplementary information 1 and included a discussion on this topic in the relevant section of our results. We have added the statement: “the age of LTR insertions was estimated using the default rate of 1.3×10–8 8 substitutions per site per year, making this calculation an approximate estimation.”<br><br>4. Generally, the Gypsy content was usually more than Copia content in plant genome, please check it. If it were correct, please infer the reason.<br><br>Previous analyses on Theobroma cacao have observed similar trends, as highlighted in the referenced study (see Table S15 in doi: 10.1186/gb-2013-14-6-r53). However, it is important to acknowledge that these results were generated over a decade ago, during which time both the transposable element (TE) pipelines and classification systems have significantly evolved. Nowadays, plant TEs can be classified with greater precision, encompassing evolutionary lineages and non-autonomous LTR elements such as SoloLTRs, LARD, TRIM, TR-GAG, and BARE-2, among others.<br><br>The goal of our TE pipeline is to comprehensively label all identified elements, thereby enhancing our understanding of the real diversity and impact of these elements within a specific plant genome. Utilizing the constraints of our pipeline based on the TEsorter 80-80-80 rule, we discovered that many LTR elements previously classified as Copia or Gypsy by tools like LTR_retriever and RepeatModeler are in fact non-autonomous elements, like LARD, TRIM, BARE2, and TR_GAG.<br><br>Our findings indicate that non-autonomous LTR elements constitute 12.37% of the T. grandiflorum genome, while Copia and Gypsy elements represent 18.31% and 13.18%, respectively. Notably, nearly all TR_GAG elements exhibit Gypsy-like GAG characteristics, accounting for almost 4% of the genome. Conversely, many TRIM and LARD elements were initially classified as Gypsy or Copia by LTR_retriever. However, upon closer inspection and using TEsorter, we did not identify complete domains but rather relics resembling mostly Gypsy, and in minor number Copia elements. Consequently, we have opted to reclassify these elements as TRIM or LARDs, which we believe is more appropriate for comparative and evolutionary studies.<br><br>In summary, our findings suggest that although the Gypsy content in T. grandiflorum appears to be younger than Copia, these elements seem to be decaying at a faster rate, resulting in the generation of non-autonomous representatives. Moreover, and equally importantly, we have tested our pipeline with the same parameters and settings

on several other plant genomes. We consistently observed a trend where the Gypsy content is usually higher than that of Copia. This finding may also supports the above mentioned hypothesis.
However, we acknowledge that further analyses, and a new and dedicated study is required to more appropriately substantiate this hypothesis.
Therefore, we have decided not to discuss this result in the manuscript, as it necessitates further and more in-depth analyses that are beyond the scope of this study.

5. All results of GO enrichment were better enriched with KEGG.

We agree that KEGG enrichment analyses are indeed a powerful tool for investigating genomic evolutionary trends. However, while we acknowledge the potential value of KEGG enrichment to our analysis, we have opted not to include it at this stage for several practical reasons. Firstly, incorporating KEGG enrichment analyses would require the generation of new tables, figures, and additional analyses, which would significantly expand the size of our manuscript, already considerable in length.
Additionally, it is crucial to consider that our manuscript is designed to provide a concise and focused narrative on Gene Ontology, exclusive gene families and positive gene selection. Incorporating KEGG enrichment analyses, while potentially enriching, could lead to a more extensive discussion, thereby significantly increasing the length of the manuscript. Our aim is to maintain a streamlined presentation of our findings, ensuring clarity and accessibility for the readers.
Therefore, while we appreciate the valuable suggestion from the reviewer, we have chosen to maintain the current scope of the manuscript, focusing on the analyses that have already been performed and discussed.
We hope that Reviewer #1 will understand our arguments and perspective on this matter.

6. The results about enrichment were written hastily, lots of GO function or GO numbers were just listed, the details should be abundant. Cite the Figures or tables or references in these sections.

Reviewer #1 is correct regarding the presentation of the enrichment results. We acknowledge that the initial version may have appeared hastily composed, with numerous Gene Ontology (GO) functions and numbers merely listed without sufficient detail. Taking this constructive feedback into account, we have thoroughly revised this section to provide a more comprehensive and detailed explanation of the enrichment results. We have also made sure to cite the relevant figures, tables, and references to offer a clearer and more informative context. We appreciate this valuable input and believe that these adjustments have significantly improved the clarity and depth of our manuscript.

7. In Figure 1C, the Ks distribution needs correction, the authors can refer the polyploidization of durian genome published in Plant physiology in 2019.

We believe the observed differences in the Ks peaks between our study and that of Wang et al. (Durian) can be attributed to distinct methodological approaches.
Our methodology follows the Qiao et al. approach (doi: 10.1186/s13059-019-1650-2), which essentially employs MAFFT with the L-INS-i option for sequence alignment and utilizes PAL2NAL to convert protein alignments into codon alignments. Subsequently, the Ka/Ks ratio is calculated using the γ-MYN method, which incorporates the Tamura–Nei model. Conversely, the Durian study employs the Nei-Gojobori method with additional corrections for their calculations.
Furthermore, our approach to inferring gene colinearity differs as well. We adhere to the Qiao approach, integrating it with the DupGen_finder pipeline, whereas the Durian study utilizes the ColinearScan tool for their analyses.
More importantly, our Ka/Ks calculation for T. cacao aligns precisely with that inferred by Qiao et al., as detailed in Additional File 4 of their paper, and corroborating the core eudicot γ whole-genome triplication event. To prevent any misinterpretation of the results we have presented, we have explicitly described the approach used in our work in the Material and Methods section. We have also added the Qiao et al. reference in the appropriate section of the results.

Powered by Editorial Manager® and ProduXion Manager® from Aries Systems Corporation

8. In Figure 2C, why some orders of TE loss the SD?

Indeed, for some elements, calculating the standard deviation was not feasible due to a low number of copies with complete LTRs, thereby lacking sufficient data for an accurate SD age estimation.

9. In Figure 3A, T. grandiflorum and T. cacao present highly syntenic at gene level, the software of Liftoff might detect extra genes to T. grandiflorum genome based on the T. cacao genome. This is just a suggestion.

This suggestion was valuable, and we indeed utilized Liftoff and BLAST in an attempt to identify any missing genes. However, we did not discover any additional missing gene in the T. grandiflorum genome.

10. In Figure 5A, there were 282 special genes in T. grandiflorum, please enrichment them with GO and KEGG.

Indeed, there are 282 gene families. We conducted several Gene Ontology (GO) enrichment analyses across all combinations presented in Figure 5A and did not find any statistically significant enrichment. This information has been clearly stated in the revised version of the manuscript.
Moreover, the 282 gene families list can be easily retrieved in the Table S10, and further explored in terms of important and relevant genes related to agronomical traits in Figure 5B (please see comment 11 below).

11. Figure 5B and D were from the GO enrichment, the GO numbers should be added around annotation or list them in the supplementary files.

Actually, Figure 5B and 5D are not the result of Gene Ontology (GO) enrichment analyses. Figure 5B illustrates exclusive gene families that are associated with genes important for agronomical traits, while Figure 5D showcases gene families that have expanded or contracted, also in relation to agronomical traits. These figures are accompanied by their respective supplementary Tables, specifically Tables S10, S11, and S12. To further clarify the significance of these results, we have incorporated an additional paragraph in the corresponding section of the manuscript.

12. In Figure 5C, the confidence interval of divergence time should be added.

In Figure 5C, we utilized the TimeTree5 database (doi: 10.1093/molbev/msac174) to estimate the divergence time among species. According to the TimeTree5 Frequently Asked Questions:

“Q: How did you derive confidence intervals for molecular time estimates?

A: Our primary aim is to inform users about differences that exist among studies in estimated time through the presentation of "confidence intervals." We based our confidence interval on the Empirical Rule (95%) in statistics, which states that, assuming a normal distribution, approximately 95% of the times reported across studies will fall within two standard deviations of the mean time. That is, we used the 95% Empirical Rule for a population of studies, which has an interpretation similar to confidence intervals and hypothesis testing. Intuitively, we expect that the among-study variance, obtained based on study node times as single data points, captures a variety of lower-level errors including differences in calibrations and sampling of genes and taxa. We present this confidence interval for nodes where times are available from five or more studies - otherwise a min-max range of time estimates is given (or no range if only a single estimate is available). We provide The Empirical Rule confidence interval as a quick guide to the amount of variation among studies, and we strongly recommended that researchers review individual studies and their methodologies before using any time estimates in downstream research.”

Therefore, the confidence interval was added to Figure 5C whenever this information was available. Additionally, we have incorporated details regarding this in both the

Powered by Editorial Manager® and ProduXion Manager® from Aries Systems Corporation

Material and Methods section and the Figure legend.

13.In the data availability, the weblink is not for everyone, GigaDB will record your data, so the unopened weblink might not necessary. [EDITOR's note: Indeed, our data curators can host your data in GigaDB - we can sort this out after submission of the revised version]

Ok, we will await further instructions before proceeding with any changes.

14. In the MS, disease resistance were mentioned repeatedly, the GO enrichment has been provided some evidence, it will be better to perform the KEGG analysis with the special genes and expanded or contracted genes to verify, especially stat the changes in the ko04626.

We have recently sequenced the chromosome-scale genome of another T. grandiflorum genotype, which, in contrast to the genome discussed in this manuscript, exhibits resistance to witches' broom disease (Clone C174). Consequently, we are currently undertaking comprehensive analyses, including those related to plant-pathogen interaction pathways, resistant genes, and transcriptome, for inclusion in another manuscript that is under preparation.

15. The language must be improved and modified by native academic English speaker.

In this revised version, the manuscript has been reviewed by a native English speaker.

We would like to thank Reviewer #1 for their time and dedication in reviewing our manuscript.

Reviewer #2

Reviewer #2: Rafael et al. contributed their study, "Genomic decoding of Theobroma grandiflorum (cupuassu) at chromosomal scale: Evolutionary insights for horticultural innovation". In this study, high-quality genome assembly for an important plant was generated and the authors further investigated genome characterization, genome evolution, gene families etc. The data quality is high, though some points need to be clarified. And the reported data and investigations could provide valuable inference for following studies.

This paper is generally well-prepared.

We would like to thank Reviewer #2 for their time and dedication in reviewing our manuscript.

Major comments:
1. Quality control of genome assembly. The quality of genome assembly could be better evaluated with more stringent parameters. On assembly quality control, I will recommend to always follow criteria established in Earth Biogenome Project (Report on Assembly Standards, https://www.earthbiogenome.org/assembly-standards). Please evaluate the present assemblies with the criteria from EBP project, I think, on at least some if not all the items. At least, I think Merqury results would be very informative.

Merqury: reference-free quality, completeness, and phasing assessment for genome assemblies https://genomebiology.biomedcentral.com/articles/10.1186/s13059-020-02134-9

We appreciate the reviewer comment. We have incorporated the Merqury and Inspector analysis reports (https://doi.org/10.1186/s13059-021-02527-4) in the new

Powered by Editorial Manager® and ProduXion Manager® from Aries Systems Corporation

	version of Table 1, and reorganized the first section of the results, describing the genome assembly and quality control in more detail as suggested. Additionally, we have added a new Table 1 and provided the complete output of the BUSCO scores in Supplementary Table S2.<br><br>2. Gaps in each pseudo-chromosome. Not clear if gaps are still remained, or the genome is of gap-free?<br><br>There are only three remaining gaps, each spanning approximately 100 bp. These gaps are located near the telomeric regions of chromosomes 8 and 6, and around the centromeric regions of chromosome 5. We have now stated this information in the results section and provided a new supplementary table (Table S3) to detail these findings.<br><br>3. Centromere region. How centromeres were identified? Centromeres were shown, but no description on how you did identify them. Given the high quality of genome assembly, it would be very interesting to incorporate the investigation into distribution of centromeres. A pipeline (https://github.com/ShuaiNIEgithub/Centromics (identifying centromere with multi-oimcs data, such as repeat profiling, and Hi-C chromatin contact) is helpful, and it was generally described at https://academic.oup.com/hr/article/10/1/uhac241/6775201?login=true) has already been prepared and widely applied in data analyses in some just published T2T assemblies.<br><br>Thanks for the valuable suggestion. Initially, we identified centromeres using the Tandem Repeat Finder tool, as described in our GitHub pipeline (https://github.com/amvarani/Plant_Annotation_TEs). However, we acknowledge that our previous method was simplistic and based on an outdated approach (refer to DOI: 10.1186/gb-2013-14-1-r10). Subsequently, we adopted the recommended pipeline and identified a recent publication detailing a new pipeline, named quarTeT (Lin et al., 2023), specifically designed for identifying telomeric and centromeric repeats. Employing both pipelines enabled us to predict the telomeric and centromeric regions of the genome effectively. In summary, both pipelines yielded comparable results, which we combined to enhance the robustness of our predictions for telomeric and centromeric regions.<br>The centromeric repeats we identified were present in all chromosomes and aligned with findings from previous cytogenetic studies (e.g. https://doi.org/10.1590/S1415-47572009005000103). We incorporated some minor adjustments to Figure 3 and included the necessary details in the legend of Figure 3, as well as in the Results and Material and Methods sections. Additionally, we have added two new supplementary tables (Table S4 and S5) that provide a summary of the identified telomeres and centromeres, now available for reference.
**Additional Information:**	
**Question**	**Response**
Are you submitting this manuscript to a special series or article collection?	No
**Experimental design and statistics**<br><br>Full details of the experimental design and statistical methods used should be given in the Methods section, as detailed in our Minimum Standards Reporting Checklist. Information essential to interpreting the data presented should be made available in the figure legends.	Yes

Powered by Editorial Manager® and ProduXion Manager® from Aries Systems Corporation

Have you included all the information requested in your manuscript?	
**Resources**<br><br>A description of all resources used, including antibodies, cell lines, animals and software tools, with enough information to allow them to be uniquely identified, should be included in the Methods section. Authors are strongly encouraged to cite Research Resource Identifiers (RRIDs) for antibodies, model organisms and tools, where possible.<br><br>Have you included the information requested as detailed in our Minimum Standards Reporting Checklist?	Yes
**Availability of data and materials**<br><br>All datasets and code on which the conclusions of the paper rely must be either included in your submission or deposited in publicly available repositories (where available and ethically appropriate), referencing such data using a unique identifier in the references and in the “Availability of Data and Materials” section of your manuscript.<br><br>Have you have met the above requirement as detailed in our Minimum Standards Reporting Checklist?	Yes

Manuscript

Click here to access/download;Manuscript;Cupuassu_Genome-

Genomic decoding of *Theobroma grandiflorum* (cupuassu) at chromosomal scale: Evolutionary insights for horticultural innovation

Rafael Moysés Alves[1*], Vinicius A. C. de Abreu[2*], Rafaely Pantoja Oliveira[3], João Victor dos Anjos Almeida[3], Mauro de Medeiros de Oliveira[3], Saura R. Silva[4], Alexandre R. Paschoal[5,6], Sintia S. de Almeida[2], Pedro A. F. de Souza[2], Jesus A. Ferro[3], Vitor F. O. Miranda[4], Antonio Figueira[7], Douglas S. Domingues[8], Alessandro M. Varani[3]

[1] Embrapa Amazônia Oriental, 66095-903 Belém, PA, Brazil - rafael-moyses.alves@embrapa.br

[2] Laboratório de Bioinformática e Computação de Alto Desempenho (LaBioCad), Faculdade de Computação (FACOMP), Universidade Federal do Pará, 66075-110 Belém, PA, Brazil – vini.abreu@gmail.com, sintiaalmeida@gmail.com, pedfar321@gmail.com

[3] Departamento de Biotecnologia Agropecuária e Ambiental, Universidade Estadual Paulista (UNESP), Faculdade de Ciências Agrárias e Veterinárias, 14884-900 Jaboticabal, SP, Brazil - rafaely.pantoja@unesp.br, joao.anjos@unesp.br, mauromedeiros@alumni.usp.br, jesus.ferro@unesp.br, alessandro.varani@unesp.br

[4] Departamento de Biologia, Universidade Estadual Paulista (UNESP), Faculdade de Ciências Agrárias e Veterinárias, 14884-900 Jaboticabal, SP, Brazil - saura.silva@unesp.br, vitor.miranda@unesp.br

[5] Departamento de Ciência da Computação (DACOM), Grupo de e Bioinformática e Reconhecimento de Padrões (bioinfo-cp), Universidade Tecnológica Federal do Paraná (UTFPR), 80230-901 Cornélio Procópio, PR, Brazil - paschoal@utfpr.edu.br

[6] Artificial Intelligence and Informatics, The Rosalind Franklin Institute, Didcot, UK

[7] Centro de Energia Nuclear na Agricultura (CENA), Universidade de São Paulo, Piracicaba, SP, Brazil - figueira@cena.usp.br

[8] Departamento de Genética, Universidade de São Paulo (USP), Escola Superior de Agricultura Luiz de Queiroz (ESALQ), Piracicaba, SP, Brazil - dougsd@usp.br

* These authors contributed equally to this work

Corresponding author: alessandro.varani@unesp.br

Running title: *Theobroma grandiflorum* genome

Abstract

Background. *Theobroma grandiflorum* (Malvaceae), known as cupuassu, is a tree indigenous to the Amazon Basin, valued for its large fruits and seed-pulp, contributing notably to the Amazonian bioeconomy. The seed-pulp is utilized in desserts and beverages, and its seed butter is used in cosmetics. Here, we present the sequenced telomere-to-telomere cupuassu genome, disclosing features of the genomic structure, evolution, and phylogenetic relationships within the Malvaceae. **Findings.** The cupuassu genome spans 423 Mb, encodes 31,381 genes distributed in the ten chromosomes, and it exhibits approximately 65% gene synteny with the *T. cacao* genome, reflecting a conserved evolutionary history, albeit punctuated with unique genomic variations. The main changes are pronounced by bursts of long-terminal repeat retrotransposons expansion at post-species divergence, retrocopied and singleton genes, and gene families displaying distinctive patterns of expansion and contraction. Furthermore, positively selected genes are evident, particularly among retained and dispersed, tandem and proximal duplicated genes associated to general fruit and seed traits and defense mechanisms, supporting the hypothesis of potential episodes of subfunctionalization and neofunctionalization following duplication, and impact from distinct domestication process. These genomic variations may underpin the differences observed in fruit and seed morphology, ripening, and disease resistance between cupuassu and the other Malvaceae species. **Conclusions.** Sequencing the cupuassu genome offers a foundational resource for both breeding and conservation efforts, yielding insights into the evolution and diversity within the genus *Theobroma*.

Keywords: Amazon basin, bioeconomy, fruit pulp and seed development, genome evolution, gene loss and retention, positive selection, plant secondary metabolites.

Key points:

- We have generated a chromosome-scale genome sequence of *Theobroma grandiflorum*, revealing a 65% synteny with *T. cacao*.
- LTR retrotransposon expansion is a pivotal factor in post-divergence genomic evolution between *Theobroma* species.
- Comparative genomics have revealed genes associated with key agronomic traits, providing evolutionary insights.
- Positive selection pressure in retained duplicated genes implicated in adaptive functions and fruit-seed trait diversity.
- Cupuassu genome is a genetic resource for breeding and to boost Brazillian Amazonian bioeconomy.

Data Description

Cupuassu, a fruit-bearing tree closely related to cacao and native to the Amazon, is highly valued for its flavorful seed-pulp and fatty seeds, extensively used in the food and cosmetics industries. We conducted a comprehensive sequencing of the cupuassu genome employing PacBio HiFi technology alongside genome-wide chromatin interaction analysis via Hi-C, with Illumina sequencing. We generated a total of 1.4 million HiFi reads and 445 million Hi-C paired-reads, which were assembled into a chromosome-scale assembly. Furthermore, we generated transcriptomic data from young and fresh leaf tissues using PacBio HiFi Iso-Seq and Illumina RNA-Seq, yielding 4.5 million and 46 million paired-reads, respectively. Approximately 25% of the cupuassu genome consists of gene-coding regions, encompassing a total of 31,381 genes. Comparative genomics analyses revealed that the cupuassu genome shares a high similarity with cacao, but it also exhibits distinctive features. Notably, repetitive DNA elements, which account for at least 54% of the genome, have significantly influenced its genomic structure. Furthermore, specific genes responsible for its fruit and seed characteristics, as well as disease resistance, were identified. Overall, this work generated data that not only deepens our knowledge of cupuassu genetics but also illuminates broader aspects of plant evolution and diversity in the Amazon. It lays the groundwork for advanced breeding programs and promises to contribute significantly to the Amazonian bioeconomy.

Context

The genus *Theobroma* L. (Malvaceae) originated in the Neotropical regions, with the Amazon basin as its main ecosystem. Among the 22 *Theobroma* species [1,2], two species, *T. cacao* L. (cacao) and *T. grandiflorum* (Willd. ex Spreng.) K.Schum. (cupuassu) are of significant economic importance. Both are diploid (2*n* = 2× = 20) presenting an average genome size around 450 Mb [3]. These

species display distinct fruit and seed morphologies, which are likely the most valued parts by humans and other dispersers [4]. Cacao seeds are the main component for the chocolate and confectionery industries. In contrast, cupuassu seed pulp is used in desserts and beverages. Additionally, cupuassu seeds can be processed to create a butter highly prized in the cosmetic industry and 'cupulate,' a product akin to chocolate [5].

Cupuassu, domesticated from *T. subincanum* Mart. by Amazon indigenous populations approximately 5,000 to 8,000 years ago, has spread geographically mainly in the last two centuries [6]. In Brazil, cupuassu is especially important for small-scale farmers in agroforest systems in Pará, Amazonas, and Bahia, the leading states in its production [7]. In 2022, Brazilian cupuassu production reached about 28,800 tons of fresh seeds from 8,900 hectares, averaging 3.2 tonnes per hectare (State Secretariat for Agricultural Development. Agricultural Indicators. Belém, PA, Brazil, 2022).

Both cacao and cupuassu face substantial threats from various fungal and viral pathogens. Specifically, the witches' broom disease (WBD) and frosty pod (FP) pose major challenges in the Americas. Both diseases are caused by two basidiomycete species, *Moniliophthora perniciosa* (Stahel) Aime & Phillips-Mora and *M. roreri* (Cif.) H.C. Evans, Stalpers, Samson & Benny, respectively. These diseases significantly reduce pod yield and the overall health of infected plants, resulting in substantial economic losses [8]. While breeding programs have identified resistant cacao and cupuassu genotypes [7,8], managing WBD and FP remains challenging [9,10], impacting local producers and family farmers systems.

Numerous sequencing initiatives have been undertaken for cacao to provide insights into the genome biology, plant-pathogen interactions and to assist breeding over the past 15 years [11–16]. To date, 37 chromosome-scale *T. cacao* genomes are publicly accessible, encompassing a range of genotypes from widely cultivated to wild-collected accessions. Additionally, the genome sequence of *Herrania umbratica* R.E.Schult, a sister genus to *Theobroma* (both representatives of the

Theobromeae tribe) known as 'monkey cacao,' which exhibits unique morphology [17], is also available.

In parallel, recent investigations have delved into the genomic architecture of *T. grandiflorum*, ranging from developing the first genetic map [18], sequencing the chloroplast and mitochondrial genomes [19,20], and in comparative transcriptomics [10,21]. These latter studies shed light on the interaction between cupuassu and *M. perniciosa*, setting the groundwork for breeding programs and transgenic approaches. However, limited genomic data for *T. grandiflorum* persists, leaving gaps in understanding its genome evolution, biology and potential comparison with *T. cacao*, a key crop in the genus.

In this study, we present a detailed analysis of the *T. grandiflorum* genome, assembling a high-quality telomere-to-telomere (T2T) chromosome-scale genome. Our comparative genomic approach reveals important genomic features, distinguishing it from related species like *T. cacao* and *H. umbratica*. These insights provide critical targets for breeding and of significant importance for evolutionary biology, biotechnology, conservation and horticulture research.

Methods

Plant sampling, DNA and RNA extraction, and sequencing

Leaf samples of the cupuassu clone 1074, susceptible to WBD [18], were collected at the 'Embrapa Amazônia Oriental' collection in Belém, PA, Brazil (1.4359° S, 48.4495° W), and cataloged at the Herbarium JABU (http://jabu.jbrj.gov.br/v2), Universidade Estadual Paulista, Jaboticabal campus (Voucher JABU1370). The samples underwent a 24-h dark incubation, flash freezing in liquid nitrogen, and were transported to the Arizona Genomics Institute (Tucson, USA) for analysis. High molecular weight (HMW) DNA was extracted using a modified CTAB protocol [22], assessed for integrity and concentration via Qubit dsDNA High-Sensitivity Assay (Thermo Fisher Scientific,

Waltham, MA, USA) and NanoDrop ND-1000 (NanoDrop Technologies, Wilmington, DE, USA). DNA quality and size were confirmed with Femto Pulse and pulse-field gel electrophoresis (Femto Pulse System, Agilent Technologies, Inc, Santa Clara, CA, USA). The DNA was sheared to 10–30 Kb using a Covaris g-TUBE (Covaris, Inc, Woburn, MA, USA), purified, and sequenced on a PacBio Sequel IIe platform (PacBio, Menlo Park, CA, USA). GenomeScope 2.0 [23] and KMC v3.2.1 [24] were employed for genome profiling.

Total RNA was extracted using the PureLink Plant RNA Reagent (Thermo Fisher Scientific Inc) and Takara NucleoSpin® RNA Clean-up (Takara Bio Inc, Kusatsu, Shiga, Japan). RNA integrity was confirmed by a 2100 Bioanalyzer (Agilent Technologies, Santa Clara, CA, USA), and only samples with an RNA Integrity Number above 7 proceeded to sequencing. IsoSeq library preparation and sequencing were performed on a PacBio Sequel IIe, while Illumina sequencing (2x100bp) was conducted on a HiSeq 2000 platform (Illumina, Inc, San Diego, CA, USA) at NGS Soluções Genômicas, Brazil.

For HiC library preparation and sequencing, samples were processed at Novogene Bioinformatics Technology (Beijing, China) using the Proximo™ Hi-C Kit (Seattle, WA, USA). The quality control was conducted using Phase Genomics' hic_qc scripts (https://github.com/phasegenomics/hic_qc) (commit: 6881c33).

Genome assembly and quality evaluation

PacBio HiFi reads were assembled employing Hifiasm v0.19.3-r572 [25] with default parameters. Contaminants were removed using kraken2 [26] and "extract_kraken_reads.py" v1.2 (https://github.com/jenniferlu717/KrakenTools), with the PlusPFP index database (https://benlangmead.github.io/aws-indexes/k2) (version 5/17/2021). The primary assembly was indexed with BWA v0.7.17-r1188 [27], and *Dpn*II restriction sites were created using the Juicer pipeline v1.6 [28]. Genome scaffolding and chromosomal reconstruction were achieved using 3D-

DNA v180419 [29], and manually corrected with Juicebox Assembly Tools v3.1.4 [28]. The final chromosome-level assembly was refined using the “run-ASM-pipeline-post-review.sh” script from 3D-DNA and “close_scaffold_gaps.sh” from the MaSuRCA assembler package v4.1.0 [30]. The adopted chromosome numbering was based on that used for *T. cacao*.

For the *H. umbratica* accession Fairchild (BioProject: PRJNA383741), we re-assembled the genome using the MaSuRCA hybrid approach with PacBio CLR and Illumina reads. Genome scaffolding for this genotype employed the Arima Genomics' mapping pipeline (https://github.com/ArimaGenomics/mapping_pipeline) (commit: 2e74ea4) and YaHS v1.1 [31].

The *T. grandiflorum* assembled genome quality and completeness were validated using Merqury v1.3 [32], Inspector v1.2 [33], LTR Assembly Index (LAI) [34], and BUSCO (Benchmarking Universal Single-Copy Orthologs) v5.4.5 against the embryophyta_odb10 database [35,36].

Transcriptome and IsoSeq Assembly

IsoSeq transcripts fasta file was generated using SMRT Link 12.0 (PacBio) with default parameters. *De novo* assembly of RNAseq short-reads and HiFi reads were performed using Trinity pipeline v2.14.0 [37]. For genome-guided transcriptome assembly, the short-reads and HiFi reads were separately aligned to the chromosome-scale genome using histat2 v2.2.1 [38] and minimap2 v2.24-r1122 [39], respectively. The aligned BAM files from both read types were then merged using StringTie2 v2.2.1 [40] to produce a GTF file, which was utilized in the genome annotation process.

We employed the PASA v2.5.3 pipeline [41], integrating IsoSeq fasta, *de novo*, and genome-guided assemblies with StringTie2, along with TransDecoder v5.7.0 (Haas, BJ. https://github.com/TransDecoder/TransDecoder) to create a comprehensive transcriptome database and to annotate transcript structures (Supplementary Information 1). This methodology was applied

to both *T. cacao* v2 (Belizian Criollo B97-61/B2 cultivar) [12] and *H. umbratica* (Fairchild) transcriptomes, using public short-reads from the GenBank Sequence Read Archive (Table S1).

The completeness of the assembled transcriptome was assessed using BUSCO v5.4.5 against the embryophyta_odb10 database in transcriptome mode.

Genome Annotation and Comparative Analyses

The genome annotation was carried out in two phases, following best practices in plant genome annotation [42]. Detailed methodologies are delineated in Supplementary Information 1.

In the first phase, Transposable Elements (TEs) and other repetitive sequences were identified and annotated utilizing an in-house pipeline (https://github.com/amvarani/Plant_Annotation_TEs) based on the Extensive *de novo* TE Annotator (EDTA) v2.0.1 [43]. Subsequently, the soft-masked genome sequence was further annotated through the integration of gene predictors and combination tools and functional annotation software, including BRAKER v3.0.4 [44], EVidence Modeler v2.1.0 [45], PASA [41] and BLAST2GO Basic v6.0 [46]. Identification of telomeric and centromeric repeats was accomplished using the quarTeT tool (commit: e1a2f72) [47] and the Centromics pipeline (https://github.com/ShuaiNIEgithub/Centromics) (commit: fe15656) [48], respectively.

The genome map was created using shinyCircos-V2.0 [49]. Whole-genome duplication (WGD) and positive selection analyses followed established methods [50]. In summary, WGD-derived gene pairs were identified using the *DupGen_finder* pipeline (https://github.com/qiao-xin/DupGen_finder) (commit: 8001838). For each duplicate pair of duplicated gene, the protein sequences were aligned using MAFFT v7.490 [51] with the L-INS-i option. These protein alignments were then converted into a codon alignment using PAL2NAL v14 [52]. The nonsynonymous (Ka) and synonymous (Ks) substitution rates were calculated using the γ-MYN method [53], as implemented in KaKs_Calculator 2.0 [54] by applying the Tamura–Nei

model [55]. Ks values exceeding 5.0 were omitted from subsequent analyses to avoid complications arising from saturated substitutions at synonymous sites. Macrosynteny and microsynteny were analyzed using MCScanX (commit: b1ca533) [56], SynVisio [57], and the Python version of MCscan (https://github.com/tanghaibao/jcvi/wiki/MCscan-(Python-version)) (commit: 09dcb9a) [58], with synteny percentages computed using custom Python scripts based on MCscan outputs.

Chromosome plots were generated with the jcvi miscellaneous plotting tool (https://github.com/tanghaibao/jcvi/wiki/Miscellaneous-plotting) (commit: 09dcb9a) and the MG2C tool v2.1 [59]. TE distribution relative to genes was determined using TE_Density (commit: 09b3e90) [60]. The TE distribution plot was generated with RAWgraphs v2.0 [61]. Orthologous gene clusters (gene families) were identified using OrthoVenn3 [62] and the OrthoFinder2 algorithm v2.5.5 [63] with diamond v2.0.14 in super-sensitive mode [64]. Gene family evolution was analyzed using CAFE 5 v1.1 [65]. For comparative purposes and to root the phylogenetic tree, the cotton D genome (*Gossypium raimondii*) v. 2.1 [66] and *Arabidopsis thaliana* (version Araport11) [67] were employed. The divergence time between the analyzed species were estimated using the TimeTree5 resource [68].

Gene Ontology (GO) enrichment analyses were performed with GOATOOLS (commit: eff7681) [69], considering only results with a *p*-value below 0.05 after false discovery rate correction with Benjamini/Hochberg significance test. Targeted comparative analyses focused on genes and functions previously related to seed traits and fruit characteristics, such as aroma, quality, maturation, and flavor, incorporating components like purine alkaloids, flavonoids, terpenoids, and fatty acids [11,70]. This was supplemented by literature and ~~Gene Ontology~~GO searches through the QuickGO platform [71].

Data Validation and quality control

High-Resolution Chromosome-Level Genome Assembly of *T. grandiflorum*

The chromosome-level genome assembly of *T. grandiflorum*, was achieved by integrating HiFi sequencing reads with Hi-C data. The total size of the assembled genome was 423 Mb, consisting of 10 chromosome-level scaffolds with lengths ranging from 28 to 53 Mb and heterozygosity rate of 0.61% (Figure 1A and B, Table 1, and Table S2). This assembly represents approximately 94% of the haploid genome size estimated by flow cytometry [3]. The average GC content of the cupuassu genome is 34.01%, comparable to *H. umbratica* (33.76%) and to *T. cacao* (32.14%). Moreover, the *T. grandiflorum* assembly is almost gap-free, presenting only three gap-regions located close to the telomeric repeats of the chromosomes 6 and 8, and in the centromeric region of the chromosome 5, associated with an LTR/LARD element (Table S3). Telomeric repeats were identified at both ends of seven chromosomes, whereas a single telomeric repeat was observed at one end of the remaining three chromosomes (Table S4). Centromeric repeats, identified on all chromosomes (Table S5), largely align with heterochromatic bands previously established through cytogenetic studies [72]. The chromosome-level assembly displays an elevated BUSCO score (98.4%) and LAI (15.6), both compatible to a reference quality genome. Furthermore, the assembly evaluation using Merqury and Inspector shows a very high genome completeness, mapping rate, and depth, and very low error rates, revealing a high accuracy of the assembled *T. grandiflorum* genome.

Structural Annotation and Spatial Gene Arrangement

A total of 31,381 protein-coding genes, corresponding to up to 25% of the entire genome length, were identified (Table 2 and Table S6). The structural gene annotation achieved a BUSCO completeness of 99.8%, indicating a high-quality annotation. Through RNAseq and IsoSeq read mapping, 46,625 complete coding sequence (CDS) were determined, confirming the functional isoforms in the gene models. The average gene length was 3,374 bp and CDS length 1,331 bp with 6 exons, values similar to *T. cacao* [11]. Furthermore, their distribution is evenly spread across the ten chromosomes.

The gene spatial arrangement and distribution in *T. grandiflorum* and *T. cacao* genomes show a similar pattern accordingly to their closely related evolutionary ties. This pattern includes genes from various duplications (whole-genome, tandem, proximal, transposed, dispersed) (Table S6). Analysis of Ks values and the distribution of WGD-derived gene pairs within syntenic blocks employing Gaussian mixture models, unveiled a distinct Ks peak at 2.5. This peak corresponds with the core eudicot γ whole-genome triplication (WGT) event (Figure 1 C). This observed peak is corroborated by prior studies that have identified the γ WGT event across a diverse range of plant species [50]. The core eudicot γ WGT is estimated to have occurred approximately 117 million years ago (mya) during the Lower Cretaceous [73]. This event predates the more recent species differentiation, which according to Timetree of Life Database [68] and previous molecular dating studies [74], occurred at ~14 mya for *Theobroma* species and ~18 mya between the genera of *Theobroma* and *Herrania*, both during the Miocene epoch.

The cupuassu genome contains 402 genes that have originated through RNA-mediated duplication, referred to as retrocopies, comprising 197 chimeric genes, 37 pseudogenes, and 168 retrogenes. A comparative analysis of these retrocopies with *T. cacao* and *H. umbratica* highlighted unique retrocopies in each ~~species~~Theobromeae: 67 in *T. grandiflorum*, 50 in *T. cacao*, and 34 in *H. umbratica* (Table S7). Interestingly, some of the unique retrocopies are linked to potential fruit and seed quality traits and plant development. For instance, a number of exclusive retrocopies in these species are related to serine/threonine-protein kinase, which is important for signal transduction and plays relevant roles in pathogen defense and fruit abscission [75]. Furthermore, retrocopies associated with chalcone metabolism in *T. grandiflorum* (TgrandC1074G00000001563) and embryo sac development in *T. cacao* (Tcacao-CriolloG00000031869) were also identified. Additionally, unique retrocopied transcription factors were noted, such as an auxin response factor in *T. grandiflorum* (TgrandC1074G00000000856) and WER-like transcription factors in *T. cacao* (Tcacao-CriolloG00000008811). *Herrania umbratica* unique retrocopies include genes linked to a

caffeic acid 3-O-methyltransferase-like activity (HumbraticaG00000009034) and polygalacturonase (HumbraticaG00000026833), potentially affecting fruit traits.

In *T. cacao*, non-coding RNAs (ncRNAs) have been proposed as primary regulators of gene expression [11]. In cupuassu, our annotation identified 1,178 long non-coding RNAs (lncRNAs), 1,058 small nucleolar RNAs (snoRNAs), 446 transfer RNAs (tRNAs), 126 microRNAs (miRNAs), 48 small nuclear RNAs (snRNAs), and 17 small RNAs (sRNAs). Moreover, the primary sites for 5S and 45S ribosomal DNA (rDNA) were mapped to chromosomes 2 and 7, respectively, corroborating previous rDNA localization using fluorescent *in situ* hybridization [72]. Overall, ncRNAs are relatively evenly distributed across the chromosomes. Notably, chromosome 7 has the lowest counts of tRNAs, miRNAs, and sRNAs, but holds the major rDNA (45S) locus (Figure S1 and Table S8).

TE Distribution and Impact in the Cupuassu Genome Architecture and Function

TE and repetitive elements constitute roughly 54% of the *T. grandiflorum* genome. The most abundant TE were LTR *Copia*, LTR *Gypsy*, and the non-autonomous LARD elements (Figure 2 A and Table S9). Notably, LTR *Copia* SIRE and LTR *Gypsy* Tekay were the most prevalent lineages accounting for up to 49 and 36 Mb of the genome (Figure 2B). Evolutionarily, LTR *Copia* elements had two significant peaks of expansion at 0.3 and 1.8 million years ago (mya), whereas the LTR *Gypsy* elements showed a single peak at around 0.3 mya (Figure S2). Comparative analyses reveal that the estimated ages of LTR expansions peaks in *T. grandiflorum*, *T. cacao*, and *H. umbratica* predate the Theobromeae species differentiation by more than 10 million years, as evidenced by molecular dating [74]. This finding underscores the potential significance of LTR elements in driving genomic evolution post-divergence within the Theobromeae tribe. Moreover, the insertion ages of LTR *Gypsy* and *Copia* elements in the analyzed Theobromeae genomes generally exhibit patterns similar to those observed in several plant families, including Fabaceae, Solanaceae, Poaceae, Funariceae, Salicaceae, Musaceae, Selaginellaceae, and Brassicaceae [76].

While the LTR *Copia* SIRE and LTR *Gypsy* Tekay elements are notably abundant in *T. grandiflorum*, they display unique expansion pattern and ages (Figure 2 C). Almost all members of *Copia SIRE* exhibit expansion, whereas only a subset of *Gypsy Tekay* elements show similar expansive trend. In contrast, certain LTR lineages, particularly *Copia TAR* and *Gypsy Athila*, have undergone significant proliferative events, marking their distinctive expansion. Interestingly, despite the high membership of *Copia Ivana*, *Ale*, and *Gypsy Ogre*, these lineages exhibit limited proliferation. In contrast, the Class II elements were less prominent as observed in other plant genomes, including *T. cacao* [11,77]. For instance, the MuDR/Mutator lineage is the most abundant, covering 881 Kb (0.27%) of the cupuassu genome.

The distribution of TE across cupuassu chromosomes is uniform among all TE classes and lineages (Table S8). The density of TEs around gene regions reflects their overall abundance in the genome, with LTR *Copia*, LTR *Gypsy*, and LARDs being concentrated near genes, typically located around 1.5 Kb at both up- and downstream (Figure S3). This distribution pattern supports the idea that TEs are advantageously located, rather than randomly, possibly impacting gene expression regulation and influencing regulatory networks [78].

Theobroma grandiflorum exhibits elevated syntenic relationships with cacao, and _H. umbratica_

At the macrosyntenic level, both *Theobroma* species exhibit significant genomic conservation, suggesting minimal rearrangements (Figure 3 A), an observation that corroborates with the published high-density cupuassu genetic map [18]. Notable variation occurs primarily within the pericentromeric and predicted centromeric regions, characterized by an elevated TE density, and other TE-dense regions (Figure 3 B). This pattern is consistent with what is commonly found in plant genomes and it has been previously observed in the cacao genome [11].

A closer inspection at the microsyntenic level among *T. grandiflorum*, *T. cacao*, and *H. umbratica* reveals a marked gene synteny and collinearity, especially at the subtelomeric regions

(Figure 3B and Figure S4). The three Theobromeae ~~species~~ genomes conserve at least 65% of gene synteny (Table S8). Interestingly, transposed gene pairs between these species are comparatively infrequent (around 7% on average).

Microsyntenic Insights into the Self-Incompatibility Loci of *Theobroma* and *Herrania*

Previous research identified two self-incompatibility loci in cacao, *CH1* and *CH4*, with *CH4* primarily linked to fruit drop [79]. Microsyntenic comparison of these loci in *T. grandiflorum* and *H. umbratica* revealed distinct patterns (Figure 4 A and B). CH1 is highly conserved across the three Theobromeae ~~species~~genomes, except for a missing *COMPASS-like H3K4 histone methylase* gene in *H. umbratica*, crucial in cellular network [80]. CH4, however, varies significantly; *T. grandiflorum* and *H. umbratica* sequences are conserved, but the one in *T. cacao* contains two additional truncated *GEX1* gene copies (Figure S5), presumably affecting gametophyte and embryo development, and possibly affecting fruit setting and late incompatibility in *T. cacao* [79,81]. The *CH4* locus in cacao also features many TE remnants and a complete LTR-RT from the Copia/Tork lineage close to a truncated copy of *GEX1*.

Cupuassu and cacao notably differ for fruit abscission. Cupuassu fruits naturally abscises when ripe, whereas cacao fruits need to be harvested from the tree [82,83]. We speculate that the multiple copies of the cacao *GEX1* gene, including the two truncated ones, together with the proximity of TE at the *CH4* loci, could either affect *GEX1* expression or produce non-functional *GEX1* proteins. This potential effect may be linked to the lack of fruit abscission phenotype in cacao, though this hypothesis needs further experimental investigation to be confirmed.

Comparative Analyses Reveal Exclusive Cupuassu Genes and Distinct Patterns of Gene Family Expansion and Contraction associated with fruit quality traits and defense mechanisms

A total of 282 exclusive gene families and 1,160 singletons were identified in *T. grandiflorum* (Figure 5 A), whereas 730 gene families are shared between *T. grandiflorum* and *T. cacao*, and 297 gene families are shared between *T. grandiflorum* and *H. umbratica*. Collectively, the three Theobromeae ~~species~~genomes share 1,816 gene families. The shared gene families among the three Theobromeae species exhibit only two significant GO enrichment: one related to pollen recognition (GO:0048544) and the other associated with protein localization to the cell surface (GO:0034394). Further GO enrichment analyses did not identify any statistically significant enrichment among the other shared and exclusive gene families. Among the exclusive and shared gene families and singletons, many are linked to fruit quality, maturation, development of organoleptic characteristics, general plant development, and resistance to pathogens (Figure 5 B and Tables S10 and S11).

Moreover, the analysis of gene expansion and contraction revealed distinct patterns across Malvaceae (Figure 5 C). Despite the GO enrichment analyses did not indicate any other statistically significant enrichment, we were able to determine specific gene functions related to important agronomical traits, indicating groups of gene families that were expanded and contracted in each species (Figure 5 D and Table S12).

We found that unique profiles of singletons and gene families (both expanded and contracted) are primarily categorized as cytochrome P450, ABC transporters, and other functions related to plant development and pathogen defense. This indicates specific adaptations and responses to domestication, environmental changes, and response to various stresses. For instance, numerous gene families and singletons genes belonging the PMD domain-containing protein identified uniquely in *T. grandiflorum* and *T. cacao* likely plays a role in developmental control [84], while the singletons genes encoding to chitin receptor/chitinase (i.e. TgrandC1074G00000003550 and TgrandC1074G00000000752) may be crucial for fungal resistance.

Exclusive gene profiles associated with fruit and seed quality, notably in lipid storage and secondary metabolite functions were identified (Figure 5 B and D). The storage lipids in seeds are key components of the quality of cocoa butter and chocolate in cacao, and in cupulate and cosmetic products in cupuassu [11,85]. Additionally, unique gene profiles involved in flavonoid, chalcone, terpenoid, and sesquiterpene metabolism might contribute to the distinct aromas of cacao and cupuassu seeds. Furthermore, different profiles in purine alkaloid metabolism could explain the flavor differences between both *Theobroma* species.

Moreover, distinct pattern of enzymes, such as methyltransferase, glycosyltransferase, and phytocyanin were identified, all crucial to secondary metabolism and related to fruit traits. Methyltransferases are key in secondary metabolite metabolism (phenylpropanoids, flavonoids, alkaloids) affecting flavor, pulp, and seed testa color [86–88]. Glycosyltransferases, catalyzing glycosylation reactions for various substrates, including plant hormones and secondary metabolites, affect fruit ripening and seed development [89,90]. Additionally, the unique gene pattern of phytocyanin, involved in growth and stress resilience [91], may be linked to the adaptability in challenging environmental conditions.

In summary, these findings corroborate the hypothesis that, despite a high number of shared gene families among Malvaceae genomes, each species exhibits unique gene families and singleton genes, and specific instances of gene family expansion and contraction, which affect developmental, defense, and adaptive functions, as well as biosynthetic pathways. Such gene families and singletons are potentially associated with the unique fruit morphologies observed, which in turn, may affect the specific traits of each *Theobroma* species, like flavor, aroma, and bioactive compound content.

Spatial Arrangement and Distribution of Duplicated Genes Reveals Evolutionary Insights into Fruit and Seed Quality and Defense Mechanism Origins

The spatial gene arrangement and distribution in the genome of the *T. grandiflorum*, *T. cacao* and *H. umbratica* was evaluated by comprehensive GO enrichment analyses (Figure 6 and Table S13). The analysis centered on GO terms, both directly and indirectly associated with fruit and seed quality as well as defense mechanisms, and it uncovered distinct functional variations across different types of gene duplications. The duplications include whole-genome duplications (WGD events), as well as tandem, proximal, dispersed, and singleton duplicates, highlighting the complex evolutionary dynamics influencing these key traits.

Cellular Component Ontology Trends and Variations

Most of the 'Cellular Component' GOs tend to be predominantly enriched in singleton genes in the three Theobromeae ~~species~~genomes. However, *T. grandiflorum* uniquely exhibited enrichment in WGD-derived genes associated with 'mitochondrion' (GO:0005739) and tandem genes linked to 'membrane' (GO:0016020). In contrast, *T. cacao* showed an enrichment of 'membrane-associated' (GO:0016020) singleton genes. Additionally, *T. grandiflorum* also showed enrichment to 'chloroplast' (GO:0009507) in dispersed duplicates, and 'cell periphery' (GO:0071944) in proximal and tandem duplicated genes, with an exclusive enrichment of 'ATPase complex' (GO:1904949) in singleton genes (Cellular Component panel of Figure 6).

Molecular Function Ontology Trends and Variations

Generally, 'Molecular Function' GOs show enrichment in tandem duplicates. In particular, 'methyltransferase activity' (GO:0008168), which is implicated in various physiological processes including fruit development [86], is found to be enriched among singleton genes. Furthermore, 'DNA-binding transcription factor activity' (GO:0003700) is enriched in WGD-derived genes. Notably, 'sulfotransferase activity' (GO:0008146), potentially influencing flavonoid metabolism [92], was enriched in both proximal and tandem duplicates of *T. caca*o. In contrast, this activity was enriched exclusively in tandem duplicates in the cupuassu and *H. umbratica* genomes. Furthermore,

‘Chitinase activity’ (GO:0004568), likely associated to defense against fungal pathogens [21], was enriched only in *T. grandiflorum* tandem duplicated genes (Molecular Function panel of Figure 6).

GO Terms Related to Fruit and Seed Traits

Numerous GO terms potentially related to fruit and seed traits were identified as enriched in duplicated genes. This is particularly prominent for ‘terpene synthase activity’ (GO:0010333), which shows enrichment in both tandem and proximal duplicates. Additionally, GO terms associated with ‘secondary metabolite biosynthesis’ (GO:0044550), ‘lipid metabolic process’ (GO:0006629), ‘phenylpropanoid biosynthesis’ (GO:0009699), ‘catechol oxidase activity’ (GO:0004097), and ‘carboxypeptidase activity’ (GO:0004180) were predominantly enriched in tandem genes. Notably, GO terms related to the ‘organonitrogen compound metabolic process’ (GO:1901564) and’ long-chain fatty acid metabolic process’ (GO:0001676) showed diverse enrichment patterns across species. This finding is particularly noteworthy due to the distinct differences in fatty acid composition between cacao and cupuassu seeds. Specifically, cacao seeds exhibit a higher concentration of saturated fatty acids, predominantly palmitic and stearic acids, followed by desaturated fatty acids, including oleic and linoleic acids [93]. In contrast, cupuassu and *Herrania* are characterized by a richness in desaturated fatty acids and long chain fatty acids [94]. Moreover, ‘flavonoid biosynthetic process’ (GO:0009813) was observed to be enriched in proximal duplicated genes exclusively within *T. grandiflorum*. This indicates a divergent evolutionary trajectory in comparison to that of cacao, wherein flavonoids are ubiquitously present in cacao seeds. Such an observation lends additional support to the hypothesis of unique evolutionary pathways and distinct domestication processes characterizing these Theobromeae species.

GO Terms Related to Fruit Aroma and Ripening Process

The ‘cellular aromatic compound metabolic process’ (GO:0006725), which may affect fruit aroma and plant defense [95], was enriched in singletons and WGD-derived genes in all three Theobromeae species. ‘Pectinesterase activity’ (GO:0030599), potentially related to fruit ripening and cell wall fortification [96], was enriched in WGD-derived genes of *T. grandiflorum* and *T. cacao*, but not in *H. umbratica* (Figure 6). The enrichment of ‘pectinesterase activity’ (GO:0030599) in WGD-derived genes may suggests a possible evolutionary advantage in the post-duplication genomic landscape of *T. cacao* and *T. grandiflorum*, reflecting in variations in their fruit maturation timelines, cell wall composition, and responses to environmental stresses.

GO Terms Related to Fruit Morphology and Hormonal Response

Genes associated with ‘meristem maintenance and development’ (GO:0048507 and GO:0010073) and ‘anatomical structure development’ (GO:0048856) were predominantly enriched in dispersed duplicates in the three Theobromeae species. In contrast, *T. grandiflorum* genes related to ‘seed development’ (GO:0080050) and ‘flower development’ (GO:0009908) showed enrichment in WGD-derived genes. These WGD-derived genes were also enriched in terms related to ‘hormonal responses’ (GO:0009725) and ‘DNA binding transcription factor activity’ (GO:0003700), while singleton genes showed enrichment in ‘ncRNA processing’ (GO:0034470), ‘RNA splicing’ (GO:0008380), and ‘DNA damage responses’ (GO:0006974). These findings indicate that tandem and WGD-derived genes may have contributed to the evolution of complex reproductive structures and the fine-tuning of hormonal regulation. For instance, the ‘response to gibberellin’ (GO:0009739) is exclusively enriched in the WGD-derived genes of *T. grandiflorum*. Furthermore, in *T. grandiflorum*, the ‘response to auxin’ (GO:0009733) is enriched in both tandem and WGD duplicates, whereas it appears to be exclusively enriched in WGD-derived genes of *T. cacao* and *H. umbratica*. Meanwhile, singleton genes might play a pivotal role in gene regulation and response to environmental stimuli, underscoring the multifaceted genetic mechanisms underlying plant development and adaptability (Figure 6).

GO Terms Related to Defense Response and Stress Reaction

Genes involved in ‘defense response’ (GO:0006952) and ‘response to stress’ (GO:0006950) were enriched in proximal and tandem repeated genes in the three ~~species~~Theobromeae. The ‘response to biotic stimulus’ (GO:0009607) was also enriched in these gene types, whereas the ‘response to abiotic stimulus’ (GO:0009628) was more prevalent in WGD-derived genes. This observed gene enrichment patterns suggest a functional specialization among gene duplication types in plant response mechanisms. For instance, proximal and tandem repeated genes are primarily associated with defense responses and stress management, indicating their crucial role in immediate and localized reaction to biotic stressors. Conversely, genes derived from WGD show a higher association with responses to abiotic stimuli, suggesting that WGD events may have equipped plants with enhanced capabilities to adapt to a broader range of environmental challenges. This dichotomy underscores the complexity of plant defense mechanisms and highlights the evolutionary significance of gene duplication in developing versatile and robust response strategies to both biotic and abiotic stresses.

Positively Selected Retained Dispersed, Proximal and Tandem Duplications: Potential Drivers of Fruit and Pathogen Resistance Evolution?

From a general evolutionary perspective, genes derived from WGD events are typically ancient and often well-integrated into the existing genetic framework, which allows ample time to functionally diverge [97]. In contrast, genes from tandem, proximal, and dispersed duplications are generally younger, often emerging in response to environmental challenges and stressors [98,99], and possibly influenced by the domestication process. In parallel, singleton genes, often originating from genome fractionation events after WGD, play crucial roles in core cellular functions and essential physiological processes [100–102].

During evolutionary timeframe and through domestication, new genes were likely created by duplication and lost over time. Interestingly, some duplicated genes are retained and can acquire new roles (neofunctionalization) or specialize in aspects of their original function (subfunctionalization), contributing to morphological innovations and the development of new functionalities, including the enhancement of disease resistance, and increased stress adaptability [101,103].

To contextualize these evolutionary processes, we evaluated the Ka/Ks rate across different gene duplication types (Figure 7A). A significant majority of duplicated genes in *H. umbratica* (97.39%), *T. cacao* (95.37%), and *T. grandiflorum* (93.85%) are under purifying selection, a trend consistent with observations in other plant species [50]. WGD-derived genes in all species exhibit strong purifying selection with a mean Ka/Ks of 0.132. Dispersed duplicates largely follow this trend (mean Ka/Ks of 0.165), with occasional peaks suggesting a balance between purifying and positive selection.

Although the majority of proximal (mean Ka/Ks = 0.444), tandem (mean Ka/Ks = 0.331), and transposed (mean Ka/Ks = 0.329) gene pairs demonstrate a trend to be under purifying selection, there is an evident trend towards greater tolerance to variation. These findings support the hypothesis of post-speciation adaptation in these gene groups, likely related to diversification or domestication effect.

Indeed, upon detailed examination, a significant portion of duplicated genes in *T. grandiflorum* (6.15%), *T. cacao* (4.62%), and *H. umbratica* (2.6%)—associated with GO terms related to plant defense, fruit and seed traits—were found to be under positive selection (Figure 7 B, Figure S6, Table S14).

For instance, in the evolutionary battle between plants and their adversaries, defense-related genes often undergo positive selection [104,105]. This is exemplified by several clusters of tandemly duplicated genes linked to defense responses and plant disease resistance, which

demonstrate strong positive selection in *T. grandiflorum* (139 genes), *T. cacao* (40 genes), and *H. umbratica* (17 genes). Notably, in *T. grandiflorum*, a significant concentration of these genes is found in chromosomes 6, 7, and 10 (Figure S7).

A cluster of genes on chromosome 6 of *T. grandiflorum* corresponds with an identified cupuassu WBD-resistance quantitative trait locus (QTL) [18]. Within this QTL, the *TgPR3* gene encoding a chitinase was associated with WBD resistance [21]. The cupuassu genome we sequenced displays the chitinase gene (TgrandC1074G00000024418), which is encircled by a multitude of disease resistance genes located within this QTL. Some of these disease resistance genes are tandemly duplicated and exhibit signs of positive selection, suggesting a robust assembly of disease resistance genes in this specific QTL (Table S15). However, it is essential to recognize that the cupuassu genome under analysis is from a *M. perniciosa*-susceptible genotype. As a result, the evolutionary gene pattern identified may not necessarily confer resistance to WBD, but could potentially be associated with resistance to other pathogens.

In the 'terpene synthase activity' (GO:0010333), tandem arrays encoding a number of delta-cadinene synthase are under positive selection across the three Theobromeae species. This enzyme plays a role in sesquiterpene biosynthesis, crucial for plant defense and the production of compounds like gossypol in cotton seeds [106,107]. It was also considered a key candidate for studying cacao-insect resistance interplay [11]. Interestingly, *T. grandiflorum* uniquely harbors tandem repeated genes encoding a probable terpene synthase (TgrandC1074G00000007568 and TgrandC1074G00000007569), hinting at regulatory role in terpenoid biosynthesis with potential ramifications for fruit aroma and flavor. Conversely, *T. cacao* possesses positively selected tandem repeated genes encoding a potential nerolidol synthase (Tcacao-CriolloG00000024422 and Tcacao-CriolloG00000024423). In cacao, this enzyme contributes to linalool biosynthesis, producing volatile monoterpenes. Linalool can be abundant in cacao seeds and are responsible for their floral aroma in certain genotypes [108]. In grapes, this enzyme enhances the aroma of certain varieties

[109]. Additionally, in rice, it is associated with the production of an antibacterial compound effective against bacterial pathogens [110].

Within the 'flavonoid biosynthetic pathway' (GO:0009813) of *T. grandiflorum*, a gene encoding a positively selected tandem duplicated naringenin 2-oxoglutarate 3-dioxygenase (TgrandC1074G00000004751 and TgrandC1074G00000004753) may emerges as pivotal in specific flavonoid, anthocyanidins, catechins and proanthocyanidins biosynthesis. Given naringenin documented broad-spectrum biological impacts on human health [111], it is conceivable that this gene plays a role in the distinct antioxidant properties of cupuassu [112], further influencing the fruit unique taste and aroma.

Another set of tandemly duplicated genes under positive selection, potentially linked to fruit and seed characteristics, involves those engaged in the 'lipid metabolic process' (GO:0006629). Both cupuassu and cacao present distinct pattern of tandemly duplicated genes, possibly related to their unique seed properties. Specifically, cupuassu has a positively selected and tandemly duplicated gene related to lipid storage in fruits, known as patatin (TgrandC1074G00000017909 and TgrandC1074G00000017911). Originally identified in potato (*Solanum tuberosum L.*) tubers, patatin is renowned for its antioxidant potential [113] and its exceptional nutritional value, making it an appealing food additive due to its solubility and emulsifying properties [114,115].

In contrast, *T. cacao* features a tandem duplicated phospholipase A1 positively selected (Tcacao-CriolloG00000024071 and Tcacao-CriolloG00000024072), which could modulate the fruit phospholipid profile. For instance, this phospholipase may be involved in linoleic acid metabolism [114–116], central to the production of desaturated fatty acids present in cacao-derived chocolates [93]. Meanwhile, *T. grandiflorum*, displays a tandem repeated gene encoding a fatty acyl-CoA reductase enzyme (TgrandC1074G00000003252 and TgrandC1074G00000003253), potentially affecting the lipid content and composition of seeds, impacting wax biosynthesis [117] and, by extension, the fruit cuticle, water retention, and shelf life.

Furthermore, *T. cacao* possesses two dispersed duplicated and positively selected pectinesterases (Tcacao-CriolloG00000016685 and Tcacao-CriolloG00000021823) that might play a significant role in the ripening of cacao fruit. Interestingly, neither *T. grandiflorum* nor *H. umbratica* exhibit positively selected pectinesterases. This observation may be associated with the behavior of cacao tree fruits, which do not fall when ripe but remain attached to the tree until manually harvested [82].

Conclusions

Recent advancements in long-read sequencing, chromatin interaction technologies, and comparative genomics have significantly enriched our understanding of genome evolution, particularly in the *Theobroma* genus, and have contributed to insights into phenotypic variation [13,118]. These tools facilitate in-depth analysis of plant development and the determinants of disease resistance, offering substantial biotechnological implications. They are becoming increasingly essential in crop breeding to address challenges such as climate change and food security.

Our study presents a chromosome-scale genome assembly of *T. grandiflorum*, enhancing genetic resources for breeding and sustainable horticulture. We have uncovered evolutionary insights into the origins of genes linked to key agronomic traits. Furthermore, we identified unique gene families and singletons in Malvaceae species, which may be instrumental in organ development, defense, adaptation, and distinctive fruit traits. The variation in gene presence or absence (and gene family expansion and contraction) among these species might be associated to unique mechanisms of gene retention and loss, which in turn are closely related to the generation of phenotypic diversity and innovation [119]. Concurrently, we revealed that many retained duplicated genes related to plant defense, fruit, and seed production are under positive selection. This finding also aligns with known processes of phenotypic novelty emergence, leading to speciation and

diversification [101,120,121]. By providing a comprehensive candidate genes list, we aim not only to support breeding initiatives but also to deepen our understanding of the cupuassu genome biology. We believe that the results presented here lay the groundwork for advanced functional genomic interventions and tailored cultivation methods. This could potentially enhance species conservation and farmer productivity, thereby further impacting the Amazonian bioeconomy. In conclusion, our findings offer valuable insights into the unique evolutionary pathways and domestication of *T. grandiflorum* and *T. cacao*, particularly in terms of pathogen resistance, fruit and seed development and adaptive strategies post-diversification.

Additional Files

Supplementary Information 1. HMW DNA extraction, Sequencing QC, Bioinformatics procedures used to annotate *Theobroma grandiflorum, T. cacao* and *Herrania umbratica* genomes, and additional notes.

Figures

Figure S1. ncRNA distribution in *Theobroma grandiflorum* chromosomes.

Figure S2. LTR insertion time of *Gypsy* and *Copia* elements. **A.** *Theobroma grandiflorum,* **B.** *T. cacao,* and **C.** *Herrania umbratica.* The vertical black line represents the median, and the dotted line represents the mean. The age of LTR insertions was estimated using the default substitution rate of 1.3×10^{-8} substitutions per site per year, making this calculation an approximate estimation.

Figure S3. TE_density analyses of all *Theobroma grandiflorum* chromosomes.

Figure S4. A. Microsynteny and colinearity example of subtelomeric regions of *Theorboma grandiflorum*, *T. cacao* and *Herrania umbratica*, **B.** Microsynteny and colinearity example of pericentromeric regions of *T. grandiflorum*, *T. cacao* and *H. umbratica.* Blue represents genes in the forward direction, green indicates genes in the reverse direction, and orange denotes transposable elements (TEs).

Figure S5. Alignment of the *GEX1* gene from *CH4* loci generated on Jalview (Procter et al., 2021).

Figure S6. Box-plot and swarmplot showing the the Ka/Ks ratio distributions of the selected GO terms associated with fruit traits and defense mechanisms. A. *Theobroma cacao*, B. *Herrania umbratica*.

Figure S7. Genomic mapping of plant disease resistance genes in *Theobroma grandiflorum* chromosomes. Genes under positive selection are shown in red. The cupuassu WBD-resistant QTL is shown in blue.

Tables

Table S1. GenBank SRA accession numbers used for transcriptome assembly. **A.** All *Theobroma cacao* RNAseq data used. **B.** *Herrania umbratica* RNAseq data used.

Table S2. Genome assembly statistics and completeness scores of the three Theobromeae genomes (BUSCO scores were retrieved using embryophyta_odb10).

Table S3 Summary of gaps on the *T. grandiflorum* chromosomes (the genomic coordinates includes ~500bp boundaries).

Table S4. Summary of telomeres on the *T. grandiflorum* chromosomes.

Table S5. Summary of centromeres on the *T. grandiflorum* chromosomes.

Table S6. Genome annotation features and statistics of the three Theobromeae genomes.

Table S7. Retrocopies identified in *Theobroma grandiflorum*, *T. cacao*, and *Herrania umbratica,* with associated raw data.

Table S8. Genome structural features and statistics for each *Theobroma grandiflorum* chromosome.

Table S9. Transposable elements summary table and statistics identified of the three Theobromeae genomes

Table S10. Exclusive gene families identified for each Theobromeae genome analyzed.

Table S11. Singletons identified in each Theobromeae genome analyzed.

Table S12. Expanded and contracted gene families identified in each Theobromeae genome analyzed.

Table S13. GO enrichment analyses raw data.

Table S14. Genes and GO terms identified as positively selected by Ka/Ks analysis.

Table S15. Gene content and features of cupuassu WBD-resistant QTL.

Data availability

The *T. grandiflroum* sample (GenBank BioSample SAMN37717187) was included at National Genetic Heritage and Associated Traditional Knowledge Management System (SisGen) under the accession #A2A72C6. The complete genome was deposited at GenBank, BioProject PRJNA691024; the raw reads are available at GenBank Sequence Read Archive (SRA) under the accession numbers: SRR28330360, SRR28297999, SRR28289108, SRR26316970 and SRR26316971. The genome sequence, gene models and functional annotation files (GFF3s and FASTAs) are also available at our genome browser web-service: https://plantgenomics.ncc.unesp.br/gen.php?id=Theo.

Declarations

Abbreviations

BUSCO: Benchmarking Universal Single-Copy Orthologs

CDS: coding sequence

CTAB: Cetyltrimethylammonium Bromide

FP: Frosty pod

GO: Gene Ontology

HiC: Chromosome conformation capture techniques

HMW: High molecular weight

Ka: non-synonymous nucleotide substitutions

Ks: synonymous substitutions

LAI: LTR Assembly Index

LARD: Large Retrotransposon Derivatives

lncRNAs: long non-coding RNAs

LTR-RT: Long Terminal Repeat Retrotransposons

miRNAs: microRNAs

mya: million years ago

QTL: Quantitative trait locus

rDNA: ribosomal DNA

snoRNAs: small nucleolar RNAs

snRNAs: small nuclear RNAs

sRNAs: small RNAs

T2T: telomere-to-telomere

TE: Transposable Elements

TRIM: Terminal-repeat Retrotransposons in Miniature

tRNAs: transfer RNAs

WBD: Witches' broom disease

WGD: Whole-genome duplication

WGT: Whole-genome triplication

Conflict of Interest

The authors declare that they have no known competing financial interests or personal relationships that could have appeared to influence the work reported in this paper.

Funding

This study was financed by the 'Fundação de Amparo à Pesquisa do Estado do São Paulo' – FAPESP, grant #2019/25176-0 to AMV, and 'Fundação Amazônia de Amparo a Estudos e Pesquisas' – FAPESPA, grant #075/2020 to VACA and RMA. 'Fundação Araucária' supported ARP in 'NAPI Bioinformática' project grant #66.2021. AMV and DSD are currently supported by the National Council for Scientific and Technological Development (CNPq) productivity grants (304367/2022-2 and 313174/2022). These funding agencies had no role in study design, the collection, analysis, and interpretation of data, or manuscript writing.

Author Contributions

Rafael Moysés Alves: Conceptualization; Data curation; project administration; writing—original draft; writing—review and editing. **Vinicius A. C. de Abreu**: Conceptualization; Data curation; formal analysis; software; investigation; supervision; project administration; writing—review and editing. **Rafaely Pantoja Oliveira**: Formal analysis; investigation; review and editing. **João Victor dos Anjos Almeida**: Formal analysis; investigation; review and editing. **Mauro de Medeiros de Oliveira**: Resources; review and editing. **Saura R. Silva**: Resources; review and editing. **Alexandre R. Paschoal:** Investigation; methodology; software, review and editing. **Sintia Almeida:** investigation; review and editing. **Pedro A. F. de Souza:** investigation; review and editing. **Jesus A. Ferro**: resources; review and editing. **Vitor F. O. Miranda**: Resources, review and editing. **Antonio Figueira**: Investigation; writing—review and editing. **Douglas S. Domingues**: Investigation; writing—review and editing. **Alessandro M. Varani**: Conceptualization; Data curation; formal analysis; software; investigation; methodology; resources; project administration; supervision; writing—review and editing.

Acknowledgments

We thank the Arizona Genomics Institute (Tucson, AZ – USA) for providing all the HMW DNA extraction and PacBio Sequel IIe sequencing support. We would also like to express our appreciation to Vitor Gregorio for his assistance with ncRNA annotation, and to Lucilia Helena Marcellino for providing the RNA extraction protocol.

Authors' information

Rafael Moysés Alves: 0000-0002-9826-4690

Vinicius A. C. de Abreu: 0000-0002-4243-2421

Rafaely Pantoja Oliveira: 0000-0003-4907-3289

João Victor dos Anjos Almeida: 0000-0003-1255-5831

Mauro de Medeiros de Oliveira: 0000-0002-2048-6664

Saura R. Silva: 0000-0002-6333-5268

Alexandre R. Paschoal: 0000-0002-8887-0582

Sintia Almeida: 0000-0003-0270-9059

Pedro A. F. de Souza: 0009-0002-9870-0023

Jesus A. Ferro: 0000-0002-3966-1303

Vitor F. O. Miranda: 0000-0003-0574-9865

Antonio Figueira: 0000-0001-8641-2556

Douglas S. Domingues: 0000-0002-1290-0853

Alessandro M. Varani: 0000-0002-8876-3269

References

1. Cuatrecasas J. Cacao and Its Allies: A Taxonomic Revision of the Genus Theobroma. Smithsonian Inst;

2. The Angiosperm Phylogeny Group. An update of the Angiosperm Phylogeny Group classification for the orders and families of flowering plants: APG IV. *Bot J Linn Soc*. 2016; doi: 10.1111/boj.12385.

3. da Silva RA, Souza G, Lemos LSL, Lopes UV, Patrocínio NGRB, Alves RM, et al.. Genome size, cytogenetic data and transferability of EST-SSRs markers in wild and cultivated species of the genus Theobroma L. (Byttnerioideae, Malvaceae). *PLoS One*. 2017; doi: 10.1371/journal.pone.0170799.

4. Freitas ÍR, Pirani JR, Colli-Silva M. CACAU PARA QUÊ? LEVANTAMENTO BIBLIOGRÁFICO SOBRE OS USOS MATERIAIS E SIMBÓLICOS DAS ESPÉCIES DE CACAUS DO BRASIL. *Ethnoscientia - Brazilian Journal of Ethnobiology and Ethnoecology*. 2023; doi: 10.18542/ethnoscientia.v8i1.12940.

5. Garcia TB, Potiguara RC de V, Kikuchi TYS, Demarco D, Aguiar-Dias ACA de. Leaf anatomical features of three Theobroma species (Malvaceae s.l.) native to the Brazilian Amazon. *Acta Amaz*. Instituto Nacional de Pesquisas da Amazônia; 2014; doi: 10.1590/1809-4392201300653.

6. Colli-Silva M, Richardson JE, Neves EG, Watling J, Figueira A, Pirani JR. Domestication of the Amazonian fruit tree cupuaçu may have stretched over the past 8000 years. *Commun Earth Environ*. Nature Publishing Group; 2023; doi: 10.1038/s43247-023-01066-z.

7. Alves RM, Chaves SF da S. Selection of Theobroma grandiflorum clones adapted to agroforestry systems using an additive index. *Acta Scientiarum Agronomy*. 2023; doi: 10.4025/actasciagron.v45i1.57519.

8. Alves RM, Chaves SF da S. BRS Careca, BRS Fartura, BRS Duquesa, BRS Curinga, and BRS Golias: new cupuassu tree cultivars. *Crop Breed Appl Biotechnol*. Crop Breeding and Applied Biotechnology; 2020; doi: 10.1590/1984-70332020v20n4c66.

9. Leal GA, Albuquerque PSB, Figueira A. Genes differentially expressed in Theobroma cacao associated with resistance to witches' broom disease caused by Crinipellis perniciosa. *Mol Plant Pathol*. 2007; doi: 10.1111/j.1364-3703.2007.00393.x.

10. Falcão LL, Silva-Werneck JO, Albuquerque PSB, Alves RM, Grynberg P, Togawa RC, et al.. Comparative transcriptomics of cupuassu (Theobroma grandiflorum) offers insights into the early defense mechanism to Moniliophthora perniciosa, the causal agent of witches' broom disease. *Journal of Plant Interactions*. Taylor & Francis; 2022; doi: 10.1080/17429145.2022.2144650.

11. Argout X, Salse J, Aury J-M, Guiltinan MJ, Droc G, Gouzy J, et al.. The genome of Theobroma cacao. *Nat Genet*. 2011; doi: 10.1038/ng.736.

12. Argout X, Martin G, Droc G, Fouet O, Labadie K, Rivals E, et al.. The cacao Criollo genome v2.0: an improved version of the genome for genetic and functional genomic studies. *BMC Genomics*. 2017; doi: 10.1186/s12864-017-4120-9.

13. Argout X, Droc G, Fouet O, Rouard M, Labadie K, Rhoné B, et al.. Pangenomic exploration of Theobroma cacao: New Insights into Gene Content Diversity and Selection During Domestication. *bioRxiv*. Cold Spring Harbor Laboratory; 2023; doi: 10.1101/2023.11.03.565324.

14. Motamayor JC, Mockaitis K, Schmutz J, Haiminen N, Livingstone D, Cornejo O, et al.. The genome sequence of the most widely cultivated cacao type and its use to identify candidate genes regulating pod color. *Genome Biol*. 2013; doi: 10.1186/gb-2013-14-6-r53.

15. Morrissey J, Stack JC, Valls R, Motamayor JC. Low-cost assembly of a cacao crop genome is able to resolve complex heterozygous bubbles. *Hortic Res*. Nature Publishing Group; 2019; doi: 10.1038/s41438-019-0125-7.

16. Hämälä T, Wafula EK, Guiltinan MJ, Ralph PE, dePamphilis CW, Tiffin P. Genomic structural variants constrain and facilitate adaptation in natural populations of Theobroma cacao, the chocolate tree. *Proc Natl Acad Sci U S A*. 2021; doi: 10.1073/pnas.2102914118.

17. Colli-Silva M, Richardson J, Pirani J. A taxonomic dataset of preserved specimen occurrences of Theobroma and Herrania (Malvaceae, Byttnerioideae) stored in 2020. *Biodiversity Data Journal*. Pensoft Publishers; 2023; doi: 10.3897/BDJ.11.e99646.

18. Mournet P, de Albuquerque PSB, Alves RM, Silva-Werneck JO, Rivallan R, Marcellino LH, et al.. A reference high-density genetic map of Theobroma grandiflorum (Willd. ex Spreng) and QTL detection for resistance to witches' broom disease (Moniliophthora perniciosa). *Tree Genetics & Genomes*. 2020; doi: 10.1007/s11295-020-01479-3.

19. Niu Y-F, Ni S-B, Liu J. The complete chloroplast genome of Theobroma grandiflorum, an important tropical crop. *Mitochondrial DNA B Resour*. 2019; doi: 10.1080/23802359.2019.1693291.

20. de Abreu VAC, Moysés Alves R, Silva SR, Ferro JA, Domingues DS, Miranda VFO, et al.. Comparative analyses of Theobroma cacao and T. grandiflorum mitogenomes reveal conserved gene content embedded within complex and plastic structures. *Gene*. 2023; doi: 10.1016/j.gene.2022.146904.

21. Santana Silva RJ, Alves RM, Peres Gramacho K, Marcellino LH, Micheli F. Involvement of structurally distinct cupuassu chitinases and osmotin in plant resistance to the fungus Moniliophthora perniciosa. *Plant Physiol Biochem*. 2020; doi: 10.1016/j.plaphy.2020.01.009.

22. Doyle JJ, Doyle JL, editors. A rapid DNA isolation procedure for small quantities of fresh leaf tissue. *PHYTOCHEMICAL BULLETIN*.

23. Ranallo-Benavidez TR, Jaron KS, Schatz MC. GenomeScope 2.0 and Smudgeplot for reference-free profiling of polyploid genomes. *Nat Commun*. 2020; doi: 10.1038/s41467-020-14998-3.

24. Kokot M, Dlugosz M, Deorowicz S. KMC 3: counting and manipulating k-mer statistics. *Bioinformatics*. 2017; doi: 10.1093/bioinformatics/btx304.

25. Cheng H, Concepcion GT, Feng X, Zhang H, Li H. Haplotype-resolved de novo assembly using phased assembly graphs with hifiasm. *Nat Methods*. 2021; doi: 10.1038/s41592-020-01056-5.

26. Wood DE, Lu J, Langmead B. Improved metagenomic analysis with Kraken 2. *Genome Biology*. 2019; doi: 10.1186/s13059-019-1891-0.

27. Li H, Durbin R. Fast and accurate short read alignment with Burrows–Wheeler transform. *Bioinformatics*. 2009; doi: 10.1093/bioinformatics/btp324.

28. Durand NC, Shamim MS, Machol I, Rao SSP, Huntley MH, Lander ES, et al.. Juicer Provides a One-Click System for Analyzing Loop-Resolution Hi-C Experiments. *Cell Syst*. 2016; doi: 10.1016/j.cels.2016.07.002.

29. Dudchenko O, Batra SS, Omer AD, Nyquist SK, Hoeger M, Durand NC, et al.. De novo assembly of the Aedes aegypti genome using Hi-C yields chromosome-length scaffolds. *Science*. American Association for the Advancement of Science; 2017; doi: 10.1126/science.aal3327.

30. Zimin AV, Puiu D, Luo M-C, Zhu T, Koren S, Marçais G, et al.. Hybrid assembly of the large and highly repetitive genome of Aegilops tauschii, a progenitor of bread wheat, with the MaSuRCA mega-reads algorithm. *Genome Res*. 2017; doi: 10.1101/gr.213405.116.

31. Zhou C, McCarthy SA, Durbin R. YaHS: yet another Hi-C scaffolding tool. *Bioinformatics*. 2023; doi: 10.1093/bioinformatics/btac808.

32. Rhie A, Walenz BP, Koren S, Phillippy AM. Merqury: reference-free quality, completeness, and phasing assessment for genome assemblies. *Genome Biol*. 2020; doi: 10.1186/s13059-020-02134-9.

33. Chen Y, Zhang Y, Wang AY, Gao M, Chong Z. Accurate long-read de novo assembly evaluation with Inspector. *Genome Biol*. 2021; doi: 10.1186/s13059-021-02527-4.

34. Ou S, Chen J, Jiang N. Assessing genome assembly quality using the LTR Assembly Index (LAI). *Nucleic Acids Res*. 2018; doi: 10.1093/nar/gky730.

35. Kriventseva EV, Kuznetsov D, Tegenfeldt F, Manni M, Dias R, Simão FA, et al.. OrthoDB v10: sampling the diversity of animal, plant, fungal, protist, bacterial and viral genomes for evolutionary and functional annotations of orthologs. *Nucleic Acids Res*. 2019; doi: 10.1093/nar/gky1053.

36. Manni M, Berkeley MR, Seppey M, Zdobnov EM. BUSCO: Assessing Genomic Data Quality and Beyond. *Curr Protoc*. 2021; doi: 10.1002/cpz1.323.

37. Haas BJ, Papanicolaou A, Yassour M, Grabherr M, Blood PD, Bowden J, et al.. De novo transcript sequence reconstruction from RNA-seq using the Trinity platform for reference generation and analysis. *Nat Protoc*. Nature Publishing Group; 2013; doi: 10.1038/nprot.2013.084.

38. Kim D, Paggi JM, Park C, Bennett C, Salzberg SL. Graph-based genome alignment and genotyping with HISAT2 and HISAT-genotype. *Nat Biotechnol*. Nature Publishing Group; 2019; doi: 10.1038/s41587-019-0201-4.

39. Li H. Minimap2: pairwise alignment for nucleotide sequences. *Bioinformatics*. 2018; doi: 10.1093/bioinformatics/bty191.

40. Kovaka S, Zimin AV, Pertea GM, Razaghi R, Salzberg SL, Pertea M. Transcriptome assembly from long-read RNA-seq alignments with StringTie2. *Genome Biology*. 2019; doi: 10.1186/s13059-019-1910-1.

41. Haas BJ, Delcher AL, Mount SM, Wortman JR, Smith RK, Hannick LI, et al.. Improving the Arabidopsis genome annotation using maximal transcript alignment assemblies. *Nucleic Acids Res*. 2003; doi: 10.1093/nar/gkg770.

42. Vuruputoor VS, Monyak D, Fetter KC, Webster C, Bhattarai A, Shrestha B, et al.. Welcome to the big leaves: Best practices for improving genome annotation in non-model plant genomes. *Appl Plant Sci*. 2023; doi: 10.1002/aps3.11533.

43. Ou S, Su W, Liao Y, Chougule K, Agda JRA, Hellinga AJ, et al.. Benchmarking transposable element annotation methods for creation of a streamlined, comprehensive pipeline. *Genome Biol*. 2019; doi: 10.1186/s13059-019-1905-y.

44. Gabriel L, Brůna T, Hoff KJ, Ebel M, Lomsadze A, Borodovsky M, et al.. BRAKER3: Fully automated genome annotation using RNA-Seq and protein evidence with GeneMark-ETP, AUGUSTUS and TSEBRA. *bioRxiv*. 2023; doi: 10.1101/2023.06.10.544449.

45. Haas BJ, Salzberg SL, Zhu W, Pertea M, Allen JE, Orvis J, et al.. Automated eukaryotic gene structure annotation using EVidenceModeler and the Program to Assemble Spliced Alignments. *Genome Biology*. 2008; doi: 10.1186/gb-2008-9-1-r7.

46. Conesa A, Götz S, García-Gómez JM, Terol J, Talón M, Robles M. Blast2GO: a universal tool for annotation, visualization and analysis in functional genomics research. *Bioinformatics*. 2005; doi: 10.1093/bioinformatics/bti610.

47. Lin Y, Ye C, Li X, Chen Q, Wu Y, Zhang F, et al.. quarTeT: a telomere-to-telomere toolkit for gap-free genome assembly and centromeric repeat identification. *Horticulture Research*. 2023; doi: 10.1093/hr/uhad127.

48. Nie S, Zhao S-W, Shi T-L, Zhao W, Zhang R-G, Tian X-C, et al.. Gapless genome assembly of azalea and multi-omics investigation into divergence between two species with distinct flower color. *Horticulture Research*. 2023; doi: 10.1093/hr/uhac241.

49. Wang Y, Jia L, Tian G, Dong Y, Zhang X, Zhou Z, et al.. shinyCircos-V2.0: Leveraging the creation of Circos plot with enhanced usability and advanced features. *iMeta*. 2023; doi: 10.1002/imt2.109.

50. Qiao X, Li Q, Yin H, Qi K, Li L, Wang R, et al.. Gene duplication and evolution in recurring polyploidization–diploidization cycles in plants. *Genome Biology*. 2019; doi: 10.1186/s13059-019-1650-2.

51. Katoh K, Standley DM. MAFFT multiple sequence alignment software version 7: improvements in performance and usability. *Mol Biol Evol*. 2013; doi: 10.1093/molbev/mst010.

52. Suyama M, Torrents D, Bork P. PAL2NAL: robust conversion of protein sequence alignments into the corresponding codon alignments. *Nucleic Acids Res*. 2006; doi: 10.1093/nar/gkl315.

53. Wang D-P, Wan H-L, Zhang S, Yu J. Gamma-MYN: a new algorithm for estimating Ka and Ks with consideration of variable substitution rates. *Biol Direct*. 2009; doi: 10.1186/1745-6150-4-20.

54. Wang D, Zhang Y, Zhang Z, Zhu J, Yu J. KaKs_Calculator 2.0: a toolkit incorporating gamma-series methods and sliding window strategies. *Genomics Proteomics Bioinformatics*. 2010; doi: 10.1016/S1672-0229(10)60008-3.

55. Tamura K, Nei M. Estimation of the number of nucleotide substitutions in the control region of mitochondrial DNA in humans and chimpanzees. *Mol Biol Evol*. 1993; doi: 10.1093/oxfordjournals.molbev.a040023.

56. Wang Y, Tang H, Debarry JD, Tan X, Li J, Wang X, et al.. MCScanX: a toolkit for detection and evolutionary analysis of gene synteny and collinearity. *Nucleic Acids Res*. 2012; doi: 10.1093/nar/gkr1293.

57. Bandi V, Gutwin C, Siri JN, Neufeld E, Sharpe A, Parkin I. Visualization Tools for Genomic Conservation. *Methods Mol Biol*. 2022; doi: 10.1007/978-1-0716-2067-0_16.

58. Tang H, Bowers JE, Wang X, Ming R, Alam M, Paterson AH. Synteny and Collinearity in Plant Genomes. *Science*. 2008; doi: 10.1126/science.1153917.

59. Chao J, Li Z, Sun Y, Aluko OO, Wu X, Wang Q, et al.. MG2C: a user-friendly online tool for drawing genetic maps. *Mol Hortic*. 2021; doi: 10.1186/s43897-021-00020-x.

60. Teresi SJ, Teresi MB, Edger PP. TE Density: a tool to investigate the biology of transposable elements. *Mobile DNA*. 2022; doi: 10.1186/s13100-022-00264-4.

61. Mauri M, Elli T, Caviglia G, Uboldi G, Azzi M. RAWGraphs: A Visualisation Platform to Create Open Outputs. *Proceedings of the 12th Biannual Conference on Italian SIGCHI Chapter*. New York, NY, USA: Association for Computing Machinery;

62. Sun J, Lu F, Luo Y, Bie L, Xu L, Wang Y. OrthoVenn3: an integrated platform for exploring and visualizing orthologous data across genomes. *Nucleic Acids Res*. Oxford Academic; 2023; doi: 10.1093/nar/gkad313.

63. Emms DM, Kelly S. OrthoFinder: phylogenetic orthology inference for comparative genomics. *Genome Biology*. 2019; doi: 10.1186/s13059-019-1832-y.

64. Buchfink B, Reuter K, Drost H-G. Sensitive protein alignments at tree-of-life scale using DIAMOND. *Nat Methods*. Nature Publishing Group; 2021; doi: 10.1038/s41592-021-01101-x.

65. Mendes FK, Vanderpool D, Fulton B, Hahn MW. CAFE 5 models variation in evolutionary rates among gene families. *Bioinformatics*. 2021; doi: 10.1093/bioinformatics/btaa1022.

66. Paterson AH, Wendel JF, Gundlach H, Guo H, Jenkins J, Jin D, et al.. Repeated polyploidization of Gossypium genomes and the evolution of spinnable cotton fibres. *Nature*. 2012; doi: 10.1038/nature11798.

67. Cheng C-Y, Krishnakumar V, Chan AP, Thibaud-Nissen F, Schobel S, Town CD. Araport11: a complete reannotation of the Arabidopsis thaliana reference genome. *Plant J*. 2017; doi: 10.1111/tpj.13415.

68. Kumar S, Suleski M, Craig JM, Kasprowicz AE, Sanderford M, Li M, et al.. TimeTree 5: An Expanded Resource for Species Divergence Times. *Mol Biol Evol*. Oxford Academic; 2022; doi: 10.1093/molbev/msac174.

69. Klopfenstein DV, Zhang L, Pedersen BS, Ramírez F, Vesztrocy AW, Naldi A, et al.. GOATOOLS: A Python library for Gene Ontology analyses. *Sci Rep*. 2018; doi: 10.1038/s41598-018-28948-z.

70. Colonges K, Loor Solorzano RG, Jimenez J-C, Lahon M-C, Seguine E, Calderon D, et al.. Variability and genetic determinants of cocoa aromas in trees native to South Ecuadorian Amazonia. *PLANTS, PEOPLE, PLANET*. 2022; doi: 10.1002/ppp3.10268.

71. Binns D, Dimmer E, Huntley R, Barrell D, O'Donovan C, Apweiler R. QuickGO: a web-based tool for Gene Ontology searching. *Bioinformatics*. 2009; doi: 10.1093/bioinformatics/btp536.

72. Dantas LG, Guerra M. Chromatin differentiation between Theobroma cacao L. and T. grandiflorum Schum. *Genet Mol Biol*. 2010; doi: 10.1590/S1415-47572009005000103.

73. Jiao Y, Leebens-Mack J, Ayyampalayam S, Bowers JE, McKain MR, McNeal J, et al.. A genome triplication associated with early diversification of the core eudicots. *Genome Biol*. 2012; doi: 10.1186/gb-2012-13-1-r3.

74. Richardson JE, Whitlock BA, Meerow AW, Madriñán S. The age of chocolate: a diversification history of Theobroma and Malvaceae. *Frontiers in Ecology and Evolution*. 32015;

75. Hardie DG. PLANT PROTEIN SERINE/THREONINE KINASES: Classification and Functions. *Annu Rev Plant Physiol Plant Mol Biol*. 1999; doi: 10.1146/annurev.arplant.50.1.97.

76. Jedlicka P, Lexa M, Kejnovsky E. What Can Long Terminal Repeats Tell Us About the Age of LTR Retrotransposons, Gene Conversion and Ectopic Recombination? *Frontiers in Plant Science*. 112020;

77. Pedro DLF, Amorim TS, Varani A, Guyot R, Domingues DS, Paschoal AR. An Atlas of Plant Transposable Elements. *F1000Res*. 2021; doi: 10.12688/f1000research.74524.1.

78. Bourque G, Burns KH, Gehring M, Gorbunova V, Seluanov A, Hammell M, et al.. Ten things you should know about transposable elements. *Genome Biology*. 2018; doi: 10.1186/s13059-018-1577-z.

79. Lanaud C, Fouet O, Legavre T, Lopes U, Sounigo O, Eyango MC, et al.. Deciphering the Theobroma cacao self-incompatibility system: from genomics to diagnostic markers for self-compatibility. *Journal of Experimental Botany*. 2017; doi: 10.1093/jxb/erx293.

80. Stirnimann CU, Petsalaki E, Russell RB, Müller CW. WD40 proteins propel cellular networks. *Trends Biochem Sci*. 2010; doi: 10.1016/j.tibs.2010.04.003.

81. Alandete-Saez M, Ron M, Leiboff S, McCormick S. Arabidopsis thaliana GEX1 has dual functions in gametophyte development and early embryogenesis. *Plant J*. 2011; doi: 10.1111/j.1365-313X.2011.04713.x.

82. Alvim P de T. CHAPTER 10 - Cacao. In: Alvim P de T, Kozlowski TT, editors. *Ecophysiology of Tropical Crops*. Academic Press;

83. Romero Vergel AP, Camargo Rodriguez AV, Ramirez OD, Arenas Velilla PA, Gallego AM. A Crop Modelling Strategy to Improve Cacao Quality and Productivity. *Plants (Basel)*. 2022; doi: 10.3390/plants11020157.

84. Nicolau M, Picault N, Descombin J, Jami-Alahmadi Y, Feng S, Bucher E, et al.. The plant mobile domain proteins MAIN and MAIL1 interact with the phosphatase PP7L to regulate gene

expression and silence transposable elements in Arabidopsis thaliana. *PLoS Genet*. 2020; doi: 10.1371/journal.pgen.1008324.

85. G EA, A WM, P EM, Rojano BA, A JJM. Caracterización y extracción lipídica de las semillas del cacao amazónico [Theobroma grandiflorum]. *Ciencia en Desarrollo*. 2016; doi: 10.19053/01217488.4237.

86. Lam KC, Ibrahim RK, Behdad B, Dayanandan S. Structure, function, and evolution of plant O-methyltransferases. *Genome*. 2007; doi: 10.1139/g07-077.

87. Guillaumie S, Ilg A, Réty S, Brette M, Trossat-Magnin C, Decroocq S, et al.. Genetic analysis of the biosynthesis of 2-methoxy-3-isobutylpyrazine, a major grape-derived aroma compound impacting wine quality. *Plant Physiol*. 2013; doi: 10.1104/pp.113.218313.

88. Mathiazhagan M, Chidambara B, Hunashikatti LR, Ravishankar KV. Genomic Approaches for Improvement of Tropical Fruits: Fruit Quality, Shelf Life and Nutrient Content. *Genes (Basel)*. 2021; doi: 10.3390/genes12121881.

89. Wu B, Liu X, Xu K, Zhang B. Genome-wide characterization, evolution and expression profiling of UDP-glycosyltransferase family in pomelo (Citrus grandis) fruit. *BMC Plant Biol*. 2020; doi: 10.1186/s12870-020-02655-2.

90. Mendez-Yañez A, Ramos P, Morales-Quintana L. Role of Glycoproteins during Fruit Ripening and Seed Development. *Cells*. 2021; doi: 10.3390/cells10082095.

91. Bilal Tufail M, Yasir M, Zuo D, Cheng H, Ali M, Hafeez A, et al.. Identification and Characterization of Phytocyanin Family Genes in Cotton Genomes. *Genes (Basel)*. 2023; doi: 10.3390/genes14030611.

92. Hashiguchi T, Sakakibara Y, Hara Y, Shimohira T, Kurogi K, Akashi R, et al.. Identification and characterization of a novel kaempferol sulfotransferase from Arabidopsis thaliana. *Biochem Biophys Res Commun*. 2013; doi: 10.1016/j.bbrc.2013.04.022.

93. Melo CWB de, Bandeira M de J, Maciel LF, Bispo E da S, Souza CO de, Soares SE. Chemical composition and fatty acids profile of chocolates produced with different cocoa (*Theobroma cacao L.)* cultivars. *Food Sci Technol*. Sociedade Brasileira de Ciência e Tecnologia de Alimentos; 2020; doi: 10.1590/fst.43018.

94. Cohen K de O, Jackix M de NH. Características químicas e física da gordura de cupuaçu e da manteiga de cacau. Planaltina, DF: Embrapa Cerrados, 2009.; 2009;

95. Mostafa S, Wang Y, Zeng W, Jin B. Floral Scents and Fruit Aromas: Functions, Compositions, Biosynthesis, and Regulation. *Frontiers in Plant Science*. 132022;

96. Forlani S, Masiero S, Mizzotti C. Fruit ripening: the role of hormones, cell wall modifications, and their relationship with pathogens. *Journal of Experimental Botany*. 2019; doi: 10.1093/jxb/erz112.

97. Qiao X, Zhang S, Paterson AH. Pervasive genome duplications across the plant tree of life and their links to major evolutionary innovations and transitions. *Computational and Structural Biotechnology Journal*. 2022; doi: 10.1016/j.csbj.2022.06.026.

98. Wang J, Tao F, Marowsky NC, Fan C. Evolutionary Fates and Dynamic Functionalization of Young Duplicate Genes in Arabidopsis Genomes. *Plant Physiol*. 2016; doi: 10.1104/pp.16.01177.

99. Kono TJY, Brohammer AB, McGaugh SE, Hirsch CN. Tandem Duplicate Genes in Maize Are Abundant and Date to Two Distinct Periods of Time. *G3 (Bethesda)*. 2018; doi: 10.1534/g3.118.200580.

100. Duarte JM, Wall PK, Edger PP, Landherr LL, Ma H, Pires JC, et al.. Identification of shared single copy nuclear genes in Arabidopsis, Populus, Vitis and Oryza and their phylogenetic utility across various taxonomic levels. *BMC Evol Biol*. 2010; doi: 10.1186/1471-2148-10-61.

101. Panchy N, Lehti-Shiu M, Shiu S-H. Evolution of Gene Duplication in Plants1[OPEN]. *Plant Physiol*. 2016; doi: 10.1104/pp.16.00523.

102. Renny-Byfield S, Rodgers-Melnick E, Ross-Ibarra J. Gene Fractionation and Function in the Ancient Subgenomes of Maize. *Mol Biol Evol*. 2017; doi: 10.1093/molbev/msx121.

103. Rastogi S, Liberles DA. Subfunctionalization of duplicated genes as a transition state to neofunctionalization. *BMC Evol Biol*. 2005; doi: 10.1186/1471-2148-5-28.

104. Zamora A, Sun Q, Hamblin MT, Aquadro CF, Kresovich S. Positively selected disease response orthologous gene sets in the cereals identified using Sorghum bicolor L. Moench expression profiles and comparative genomics. *Mol Biol Evol*. 2009; doi: 10.1093/molbev/msp114.

105. Rech GE, Vargas WA, Sukno SA, Thon MR. Identification of positive selection in disease response genes within members of the Poaceae. *Plant Signal Behav*. 2012; doi: 10.4161/psb.22362.

106. Yoshikuni Y, Martin VJJ, Ferrin TE, Keasling JD. Engineering cotton (+)-delta-cadinene synthase to an altered function: germacrene D-4-ol synthase. *Chem Biol*. 2006; doi: 10.1016/j.chembiol.2005.10.016.

107. Karaca M, Ince AG. Grafting-induced seed gossypol levels by demethylation of (+)-delta-cadinene synthase genes in upland cotton. *Plant Breeding*. 2023; doi: 10.1111/pbr.13066.

108. Colonges K, Jimenez J-C, Saltos A, Seguine E, Loor Solorzano RG, Fouet O, et al.. Two Main Biosynthesis Pathways Involved in the Synthesis of the Floral Aroma of the Nacional Cocoa Variety. *Front Plant Sci*. 2021; doi: 10.3389/fpls.2021.681979.

109. Zhu B-Q, Cai J, Wang Z-Q, Xu X-Q, Duan C-Q, Pan Q-H. Identification of a plastid-localized bifunctional nerolidol/linalool synthase in relation to linalool biosynthesis in young grape berries. *Int J Mol Sci*. 2014; doi: 10.3390/ijms151221992.

110. Kiryu M, Hamanaka M, Yoshitomi K, Mochizuki S, Akimitsu K, Gomi K. Rice terpene synthase 18 (OsTPS18) encodes a sesquiterpene synthase that produces an antibacterial (E)-nerolidol against a bacterial pathogen of rice. *J Gen Plant Pathol*. 2018; doi: 10.1007/s10327-018-0774-7.

111. Salehi B, Fokou PVT, Sharifi-Rad M, Zucca P, Pezzani R, Martins N, et al.. The Therapeutic Potential of Naringenin: A Review of Clinical Trials. *Pharmaceuticals (Basel)*. 2019; doi: 10.3390/ph12010011.

112. Carmona-Hernandez JC, Le M, Idárraga-Mejía AM, González-Correa CH. Flavonoid/Polyphenol Ratio in Mauritia flexuosa and Theobroma grandiflorum as an Indicator of Effective Antioxidant Action. *Molecules*. 2021; doi: 10.3390/molecules26216431.

113. Liu Y-W, Han C-H, Lee M-H, Hsu F-L, Hou W-C. Patatin, the tuber storage protein of potato (Solanum tuberosum L.), exhibits antioxidant activity in vitro. *J Agric Food Chem*. 2003; doi: 10.1021/jf030016j.

114. Gambuti A, Rinaldi A, Moio L. Use of patatin, a protein extracted from potato, as alternative to animal proteins in fining of red wine. *Eur Food Res Technol*. 2012; doi: 10.1007/s00217-012-1791-y.

115. Gelley S, Lankry H, Glusac J, Fishman A. Yeast-derived potato patatins: Biochemical and biophysical characterization. *Food Chem*. 2022; doi: 10.1016/j.foodchem.2021.130984.

116. Liu W, Zhang R, Xiang C, Zhang R, Wang Q, Wang T, et al.. Transcriptomic and Physiological Analysis Reveal That α-Linolenic Acid Biosynthesis Responds to Early Chilling Tolerance in Pumpkin Rootstock Varieties. *Front Plant Sci*. 2021; doi: 10.3389/fpls.2021.669565.

117. Teerawanichpan P, Qiu X. Fatty acyl-CoA reductase and wax synthase from Euglena gracilis in the biosynthesis of medium-chain wax esters. *Lipids*. 2010; doi: 10.1007/s11745-010-3395-2.

118. Li W, Liu J, Zhang H, Liu Z, Wang Y, Xing L, et al.. Plant pan-genomics: recent advances, new challenges, and roads ahead. *Journal of Genetics and Genomics*. 2022; doi: 10.1016/j.jgg.2022.06.004.

119. Clark JW. Genome evolution in plants and the origins of innovation. *New Phytol*. 2023; doi: 10.1111/nph.19242.

120. Flagel LE, Wendel JF. Gene duplication and evolutionary novelty in plants. *New Phytol*. 2009; doi: 10.1111/j.1469-8137.2009.02923.x.

121. Birchler JA, Yang H. The multiple fates of gene duplications: Deletion, hypofunctionalization, subfunctionalization, neofunctionalization, dosage balance constraints, and neutral variation. *Plant Cell*. 2022; doi: 10.1093/plcell/koac076.

Figures and Tables Legends

Figure 1. A. Depiction of the genomic landscape of *Theobroma grandiflorum*, illustrating gene and TE density across the ten chromosomes. **B.** High-throughput chromosome conformation capture (Hi-C) contact map revealing the assembled chromosomes of *T. grandiflorum*. **C.** Whole genome duplication analyses indicating the shared whole genome triplication among *T. grandiflorum*, *T. cacao*, and *H. umbratica*, and confirming the absence of additional WGD events in these species.

Figure 2. Transposable Elements Distribution in *Theobroma grandiflorum.* **A.** Distribution of autonomous and non-autonomous TE from Class I and Class II. **B.** Distribution of all evolutionary lineages of LTR elements. **C.** Phylogenetic analysis and distribution of each full-length LTR element identified in *Theobroma grandiflorum*. The age of LTR insertions was estimated using the default rate of 1.3×10^{-8} substitutions per site per year, making this calculation an approximate estimation.

Figure 3: Comparative Genomic Analysis of *Theobroma grandiflorum* with *T. cacao* and *Herrania umbratica.* **A.** Macrosyntenic patterns between *Theobroma grandiflorum* and *T. cacao*, revealing conserved genome structures. **B.** Comparative idiogram map between *Theobroma grandiflorum* and *T. cacao*, and between *T. grandiflorum* and *Herrania umbratica*. The idiograms

illustrate gene-rich regions (blue), TE-rich regions (red), and potential location of centromeres (black circles) identified by the quarTeT and Centromics tools. Blue bars on the left of each idiogram represent microsynteny between *T. grandiflorum* and *T. cacao*, while red bars on the right indicate microsynteny between *T. grandiflorum* and *H. umbratica*.

Figure 4: Microsyntenic Analysis of the Self-Incompatibility Loci (CH1 and CH4) in Theobroma and *Herrania*. A. CH1 loci. **B.** CH4 loci. Genes marked in bold are considered central to self-incompatibility reactions, as previously described [79]. The GEX1 locus, containing the complete and homologous genes, is marked with dotted lines.

Figure 5: Comparative Analyses Across Malvaceae Species Focusing on Functions Related to Plant Differentiation, Fruit and Seed Development, and Organoleptic and Physicochemical Qualities. A. A Venn diagram illustrates the shared and exclusive orthologous clusters (gene families) identified across four Malvaceae species and *Arabidopsis thaliana*. **B.** The identification of gene families and singletons encompasses a range of functions with predicted roles in various aspects of plant and fruit development. These include Cytochrome P450 and ABC transporters, which are pivotal in synthesizing secondary metabolites and nutrient uptake, respectively, influencing plant growth and fruit quality. Plant Mobile Domain (PMD) proteins and disease resistance genes play roles in stress response and plant health, indirectly impacting fruit quality. Serine/threonine kinase, protein kinase domain-containing proteins, and several metabolism-related genes (flavonoid, chalcone, terpene, sesquiterpenes) regulate pathways critical for plant growth, development, and the organoleptic properties of fruits. Genes related to defense mechanisms (chitin receptor/chitinase, defensin, ubiquitin-like protease) and cell wall composition (methylesterase, polygalacturonase, pectinesterase, expansin, laccase, xyloglucan endotransglucosylase/hydrolase) are also identified, reflecting their roles in maintaining plant health and influencing fruit texture and

firmness. Furthermore, genes involved in seed development (vicilin, legume-related protein, lipid storage) and various transcription factors (including MADS-box) are noted for their influence on plant growth and developmental processes. **C.** A phylogenetic tree delineates the evolutionary timeline of the Malvaceae species with *A. thaliana* serving as the outgroup. An accompanying pie chart displays the proportions of gene families that have expanded or contracted, indicating evolutionary dynamics. The divergence time and its confidence interval, when available, were obtained from the TimeTree5 database. **D.** The analysis of expanded and contracted gene families focuses on their common functions and roles, as detailed in section **B**, shedding light on the evolutionary adaptations of these species.

Figure 6: Gene Ontology Enrichment and Comparative Analysis Across *Theobroma grandiflorum*, *T. cacao*, and *Herrania umbratica*. Black arrows highlight GO terms that are exclusively enriched in *T. grandiflorum*, either in duplicated genes or singletons. These terms provide insights into the unique biological processes, cellular components, and molecular functions connected with fruit and seed quality and defense mechanism that are particularly prominent in *T. grandiflorum* compared to the other species.

Figure 7: Positive Selection Analysis of Duplicated Genes in *Theobroma grandiflorum*, *T. cacao*, and *Herrania umbratica.* A. A violin plot displays the distribution of the Ka/Ks ratios for gene pairs resulting from dispersed, proximal, and tandem duplication in *Theobroma grandiflorum*, *T. cacao*, and *Herrania umbratica*. The number above each plot indicates the percentage of duplicated genes under purifying selection. **B.** A swarmplot illustrates the Ka/Ks ratio distributions for selected Gene Ontology (GO) terms associated with fruit traits and defense mechanisms in *T. grandiflorum*. This plot provides insights into the selective pressures acting on genes related to these specific functions.

Table 1. Statistics of *Theobroma grandiflorum* genome sequencing and assembly.

Genome Sequencing Statistics	Value
**HiC Sequencing**	
Number of HiC reads	445,532,022 (2x150 bp)
Average Phred Value	Q38
GC content of the HiC reads	38%
Same strand high-quality read pairs *	24.33% (expected > 1.5%)
Informative read pairs **	45.87% (expected > 5%)
**HiFi Sequencing**	
Number of HiFi reads	1,983,315 (30 Gbp)
N50 HiFi reads	15,327 bp
Average Phred Value	Q60
GC content of the HiFi reads (%)	35.29
k-mer heterozygosity rate	0.61%
**Genome Assembly Statistics**	
Genome length	423,916,809 bp
GC content of the genome (%)	34.01
Assembly Gaps	3
Chromosomes	10
Predicted Centromeres	10 (one for each chromosome)
Predicted Telomeres	17
**BUSCO analysis**	embryophyta_odb10 (1,614)
Complete	98.4% (1,588)
Complete and single copy	97.5% (1,574)
Complete and duplicated	0.9% (14)
Fragmented	0.9% (15)
Missing	0.7% (11)
**LTR Assembly Index (LAI)*** **	15.6
**Merqury analysis**	
Estimate base level Quality Value (QV)	67.907
k-mer completeness	88.4602
k-mer Error rate	0.0000161919%
**Inspector analysis**	
Mapping rate	95.99%
Depth	68.1158
QV	47.8364
Error rate (E, from $QV = -10\log 10E$)	0.00165%

*High-quality read pairs have minimum mapping quality >= 20, maximum edit distance <= 5, and are not duplicates.
**Informative read pairs are read pairs which have MAPQ > 0, are not PCR duplicates, and map to different contigs or >10kb apart.
*** To enhance Long Terminal Repeat (LTR) identification in *Theobroma grandiflorum*, the maximum distance between LTRs was set to 20,000 base pairs, which is expected to increase the size of intact elements. Consequently, this adjustment is anticipated to result in a marginally higher LTR Assembly Index (LAI) value [34]. For further details, please refer to Supplementary Information 1.

Table 2. *Theobroma grandiflorum* transcriptome sequencing and annotation features.

Features	Value
**HiFi Sequencing IsoSeq**	
Number of IsoSeq reads	4,632,516
N50 HiFi reads	2,050 bp
Average Phred Value	Q80
GC content of the IsoSeq reads (%)	44
**RNAseq - Illumina**	
Number of reads (2x100bp)	46,414,378
GC content of the reads (%)	44
**BUSCO analysis (transcriptome)**	embryophyta_odb10 (1,614)
Complete	98.7% (1,593)
Complete and single copy	22.5% (363)
Complete and duplicated	76.2% (1,230)
Fragmented	0.5% (8)
Missing	0.8% (13)
**Genome Annotation**	
-Number of genes	31,381
-- Number of CDSs (including isoforms)	46,671
---- complete CDS	46,625
---- start, no stop CDS	8
---- stop, no start CDS	22
---- no stop, no start CDS	16
-mean gene length	3,374 bp
-mean CDS length	1,331 bp
-mean exons per gene	6
-mean introns per gene	5
-tRNAs	446
-snRNAs	976
-miRNAs	109
% of genome covered by genes	25%
% of genome covered by CDS	14.70%
% of genome covered by TEs	53.93%
---Class I Elements	43.86%
LTR Gypsy	13.18%
LTR Copia	18.31%
LTR non-autonomous	12.37%
non-LTR	0.58%
---Class II Elements	2.36%
TIRs	1.21%
Helitron	1.15%
Other repeats	7.13%
**BUSCO analysis (Annotation)**	
Complete	99.8% (1,610)
Complete and single copy	59.5% (960)
Complete and duplicated	40.3% (650)
Fragmented	0.1% (1)
Missing	0.3% (3)

Figure1

Click here to access/download;Figure;Figure1.pdf

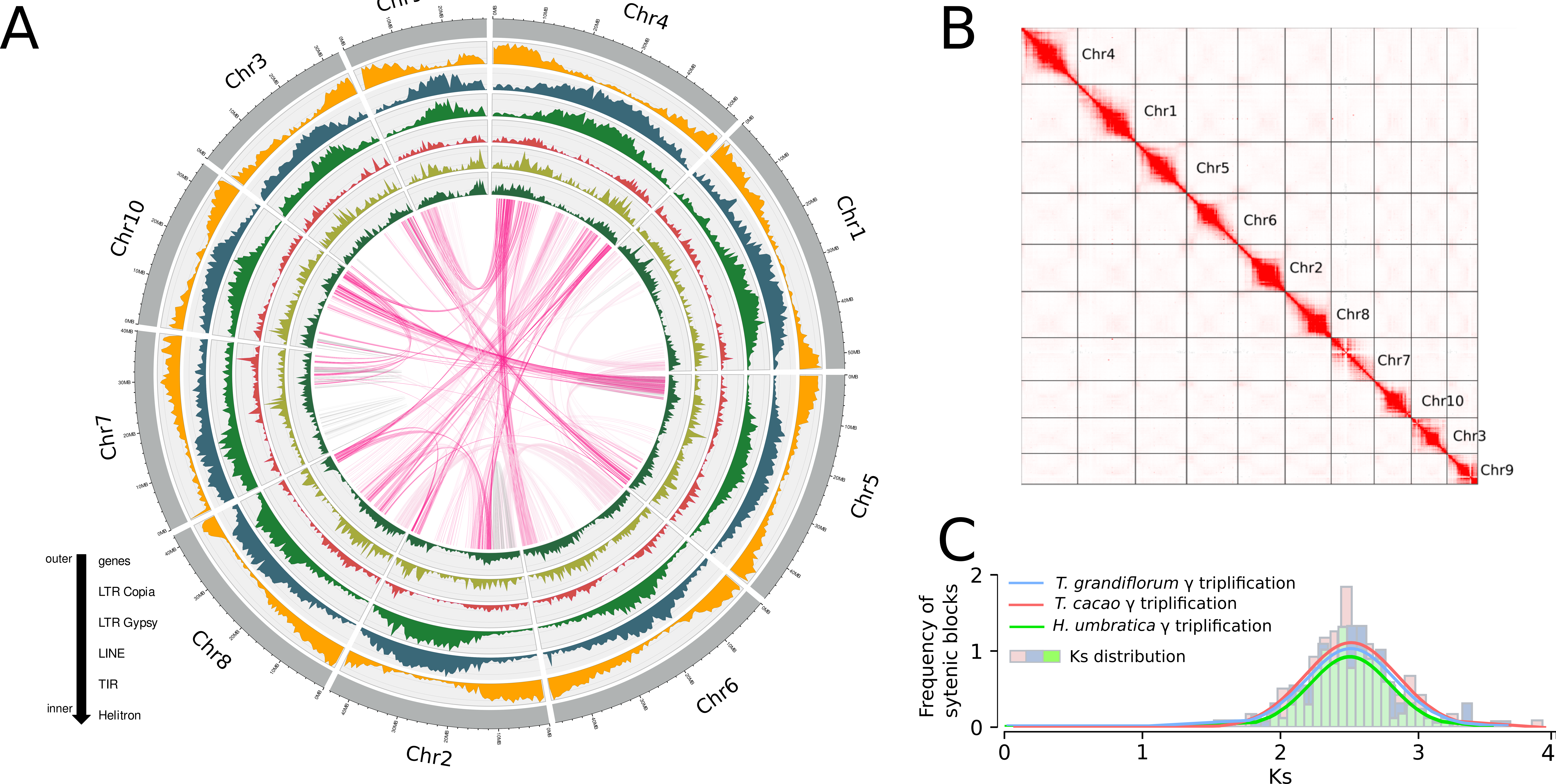

Figure2

Click here to access/download;Figure;Figure2.pdf

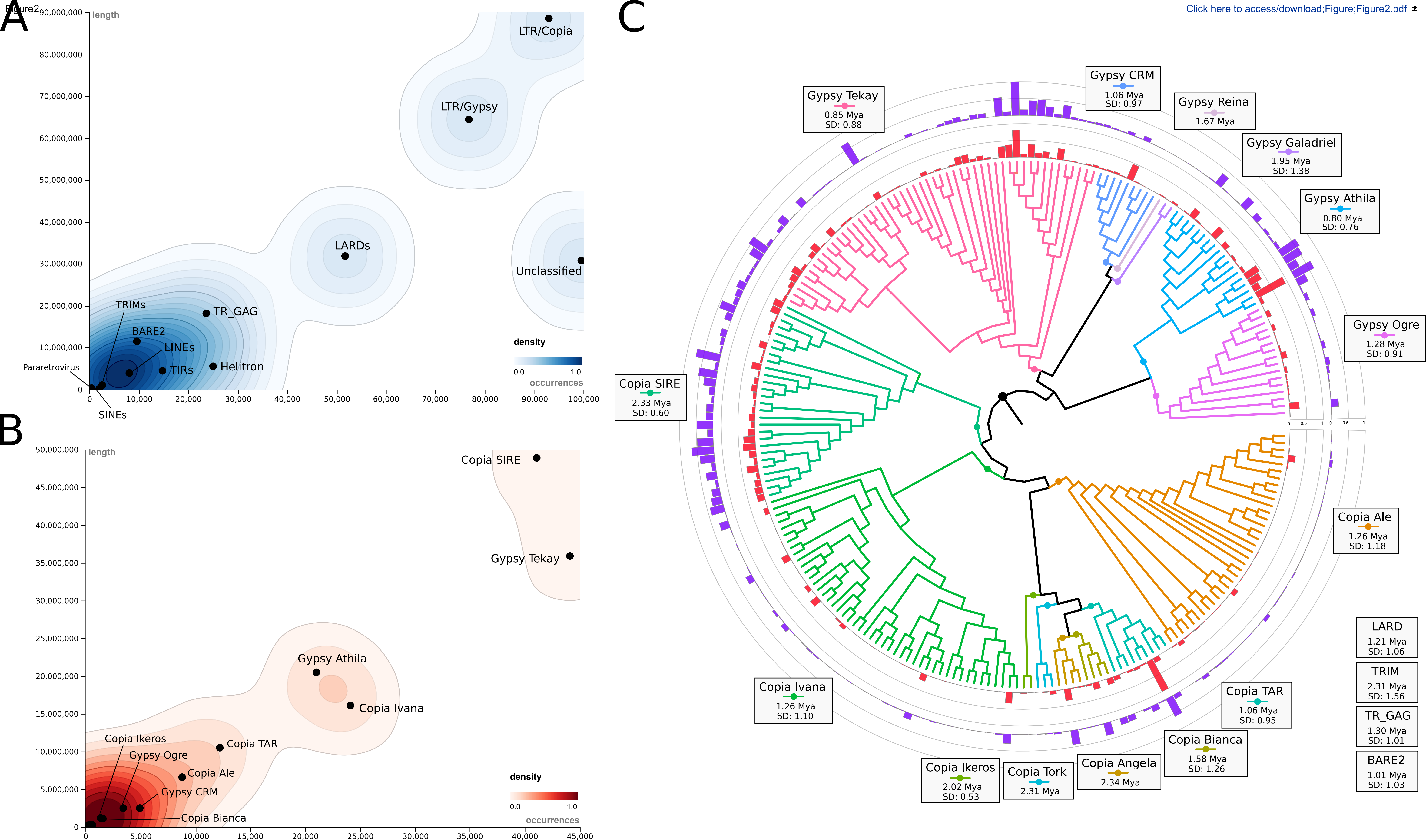

Figure3
Click here to access/download;Figure;Figure3.pdf

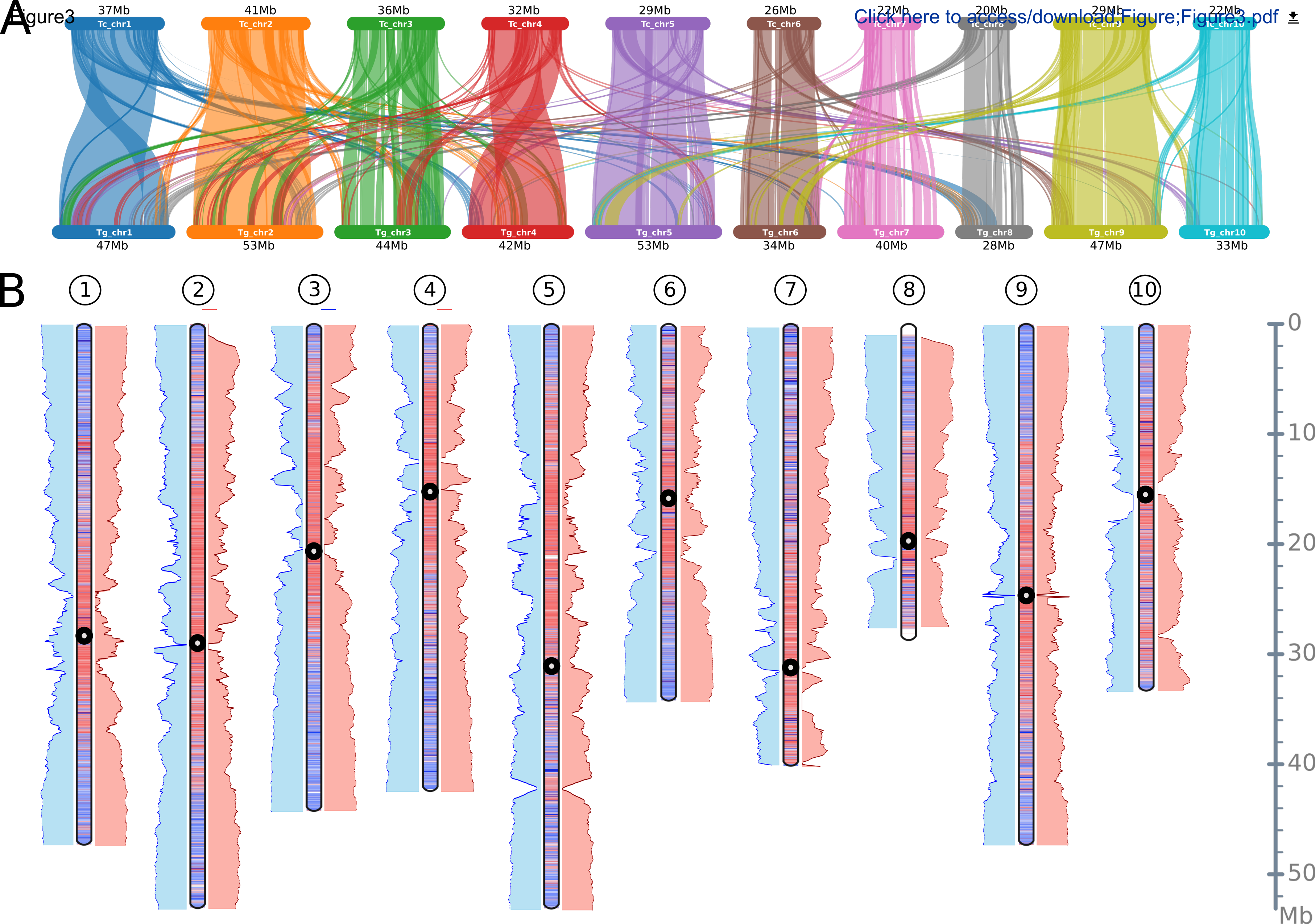

Figure4

Click here to access/download;Figure;Figure4.pdf

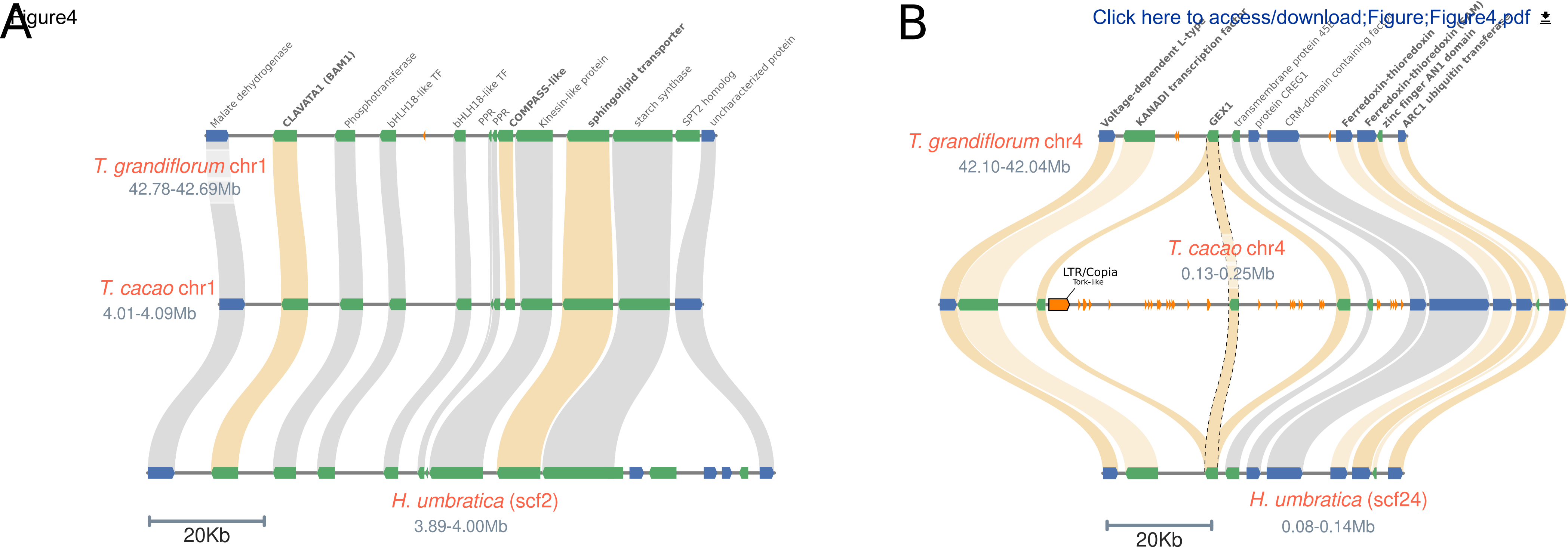

Figure5

Click here to access/download;Figure;Figure5.pdf

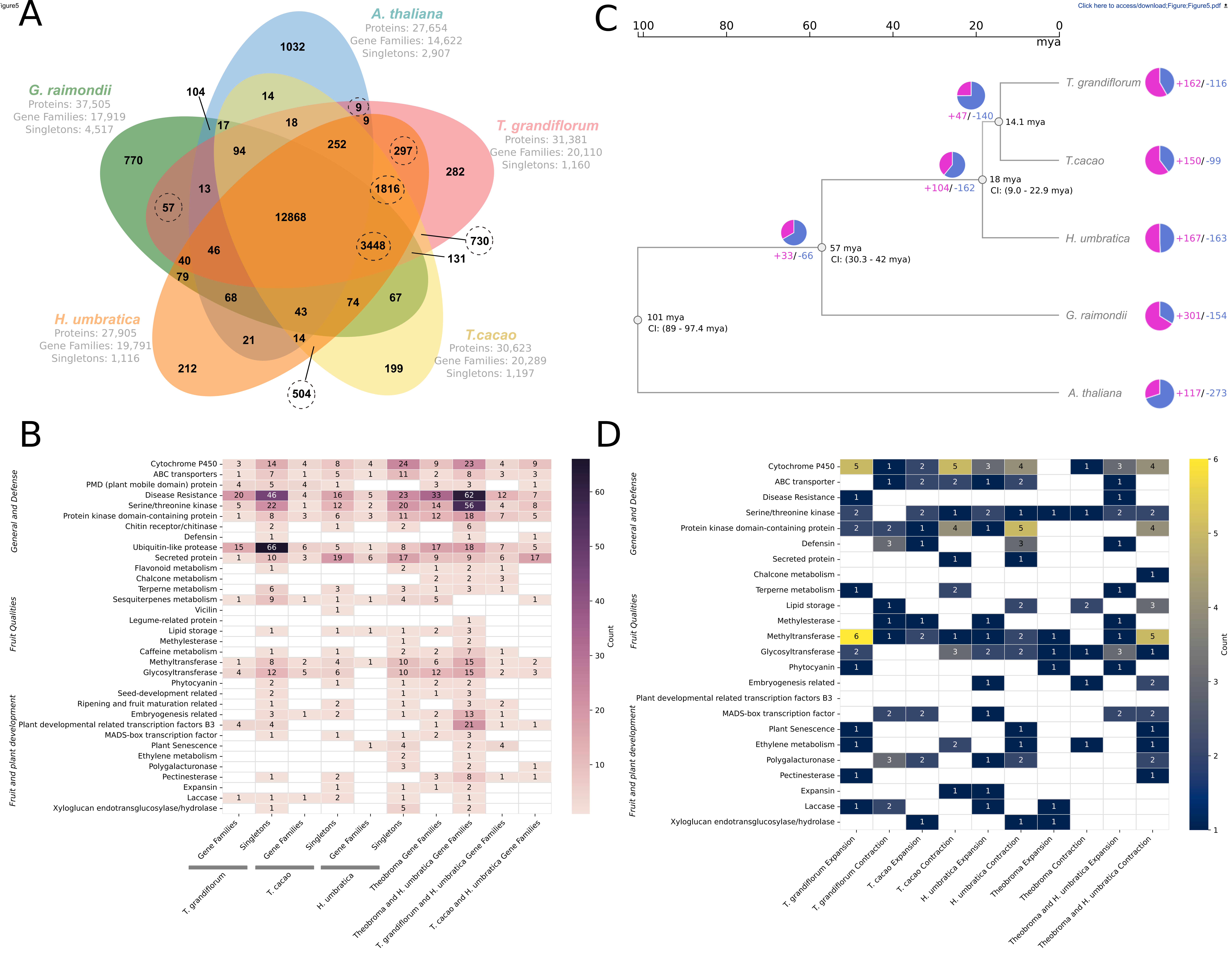

Figure6

Click here to access/download;Figure;Figure6.pdf

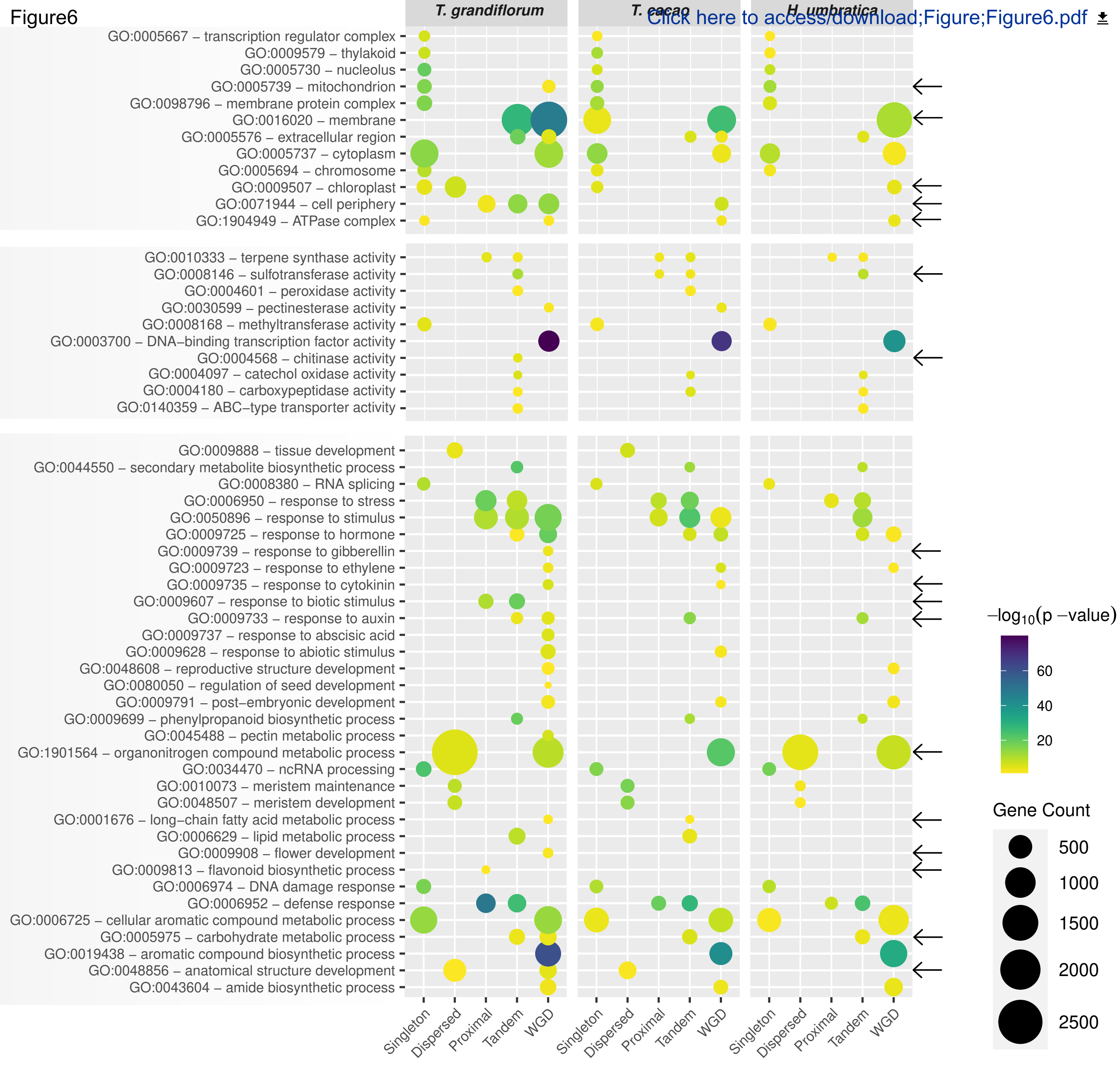

Figure7

Click here to access/download;Figure;Figure7.pdf

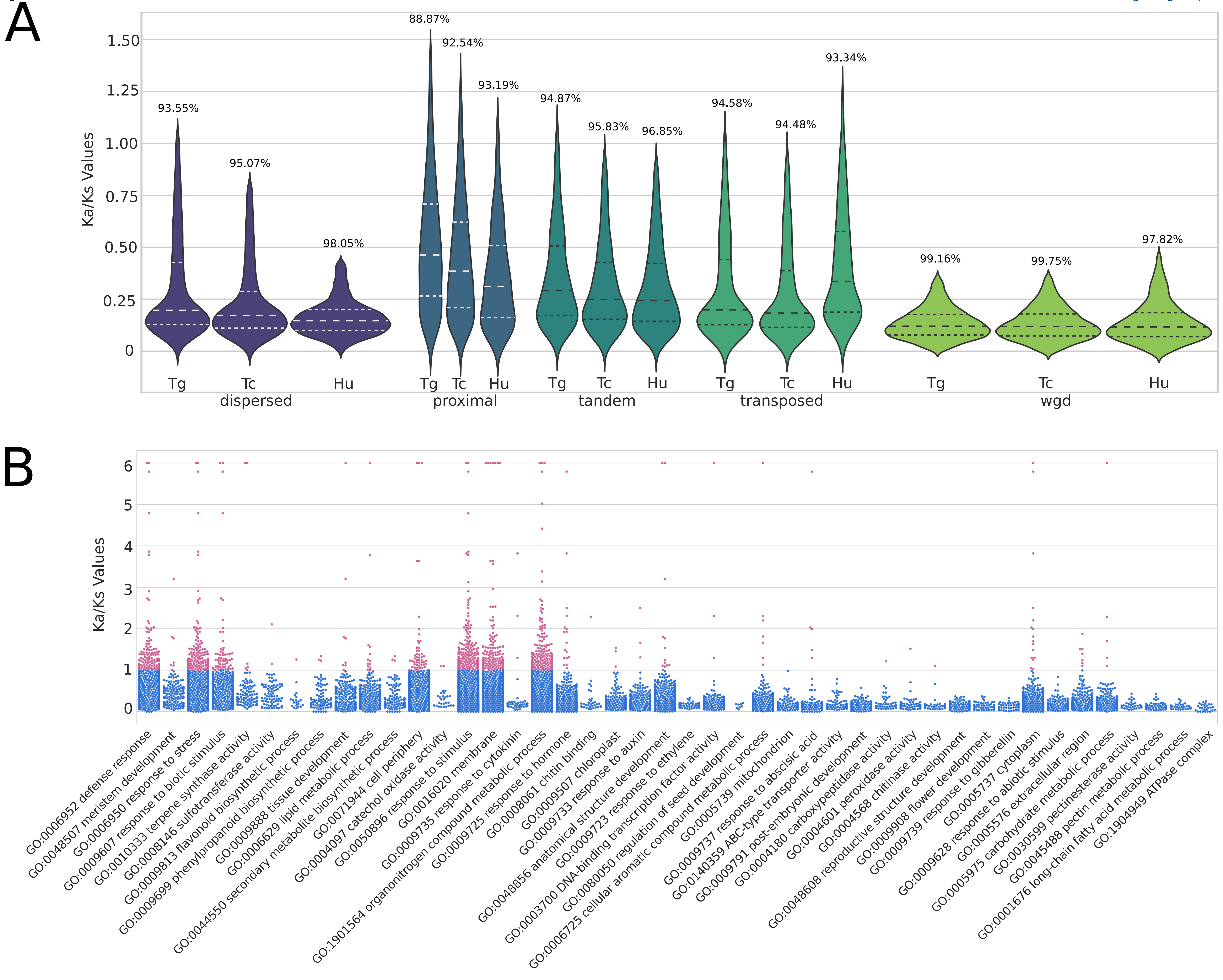

Supplementary Information 1

Click here to access/download
Supplementary Material
Supplementary_Information-GigaSciences-review1.pdf

FigureS3

Click here to access/download
Supplementary Material
FigureS3.pdf

Click here to access/download
Supplementary Material
FigureS5.pdf

FigureS6

Click here to access/download
Supplementary Material
FigureS6.pdf

FigureS7

Click here to access/download
Supplementary Material
FigureS7.pdf

Click here to access/download
Supplementary Material
TableS7.xlsx

TableS9

Click here to access/download
Supplementary Material
TableS9.xlsx

TableS10

Click here to access/download
Supplementary Material
TableS10.xls

TableS11

Click here to access/download
Supplementary Material
TableS11.xls

TableS12

Click here to access/download
Supplementary Material
TableS12.xlsx

Click here to access/download
Supplementary Material
TableS13.xlsx

TableS14

Click here to access/download
Supplementary Material
TableS14.xls

TableS15

Click here to access/download
Supplementary Material
TableS15.xls

Personal Cover
Click here to access/download;Personal Cover;cover_letter_review1.pdf

UNIVERSIDADE ESTADUAL PAULISTA
"JÚLIO DE MESQUITA FILHO"
Câmpus de Jaboticabal
Department of Agricultural and Environmental Biotechnology

Jaboticabal, 14th March 2024

Scott Edmunds Editor-in-Chief

GigaSciences

Dear Scott Edmunds, Editor-in-Chief of GigaSciences Journal,

We are pleased to resubmit our manuscript titled "**Genomic Decoding of *Theobroma grandiflorum* (Cupuassu) at Chromosomal Scale: Evolutionary Insights for Horticultural Innovation**" for consideration in GigaSciences. This submission meticulously addresses all the concerns raised by the reviewers, as detailed in our accompanying rebuttal letter. We believe that the revisions and additional data provided significantly enhance our study and underscore its contribution to the field of plant genomics.

The cornerstone of this research is the unveiling of one of the first chromosomal scale genome of a commercially significant Amazonian plant, marking a pivotal advancement in plant genetics and biotechnology. Through telomere-to-telomere sequencing of the *Theobroma grandiflorum* genome, an indigenous species to the Amazon with valuable applications in food and cosmetics, we have unveiled critical insights into its evolution and agronomic potential. The 65% synteny with *T. cacao* underscores a conserved evolutionary history, interspersed with unique genomic variations essential for understanding the diversification of fruit and seed traits, disease resistance, and evolutionary dynamics within the *Theobroma* genus.

Our response to the reviewers includes an enhanced genome quality assessment incorporating Merqury statistics as suggested, along with additional clarifications and supplementary data. These enhancements highlight the accuracy and relevance of our work, not only for academia but also for practical applications in the Brazilian Amazon bioeconomy and in addressing global challenges like climate change and food security.

UNIVERSIDADE ESTADUAL PAULISTA
"JÚLIO DE MESQUITA FILHO"
Câmpus de Jaboticabal
Department of Agricultural and Environmental Biotechnology

We firmly believe that our revised manuscript aligns well with the scope of GigaSciences. We are confident that our study will provide a robust foundation for future breeding programs.

Enclosed with this letter are the revised manuscript and all supplementary materials for your review. We confirm that this work has not been published elsewhere and is not under consideration by another journal. All authors have approved the manuscript for submission and agree with its submission to GigaSciences.

We sincerely appreciate the opportunity to revise our work and look forward to the possibility of contributing to the esteemed collection of research in GigaSciences.

Sincerely,

Alessandro M. Varani

Alessandro M. Varani
UNESP-FCAV
