# Supplementary material for: Genomic decoding of Theobroma grandiflorum (cupuassu) at chromosomal scale: evolutionary insights for horticultural innovation
Source: Gigascience. 2024 Jun 5;13:giae027. doi: 10.1093/gigascience/giae027 (PMC11152179; doi:10.1093/gigascience/giae027)
Supplement: giae027_Response_to_Reviewer_Comments_Original_Submission [file giae027_response_to_reviewer_comments_original_submission.pdf]

Author's Response To Reviewer Comments

Rebuttal letter

Editor comment

Please also note, after assessing the paper with the reviewers, we feel the main advance of the paper is the presentation of a genome assembly and some more basic analyses, rather than answering a specific biological research question. We will therefore consider the revised manuscript for publication in our "Data Note" section. Please submit the revised paper as "Data Note" and change the format accordingly (see the instructions for authors on our homepage). You do not need to shorten the article, but the order and titles of sections are slightly different in a Data Note.

In response to your guidance and after careful consideration of the reviewers' assessments, we have revised our manuscript as suggested. We have formatted our submission with the "Data Note" requirements, adhering to the specific order and titles of sections as outlined in the instructions for authors. We appreciate this opportunity to present our manuscript on genome assembly and analyses within the framework of a "Data Note."
Please note that all modifications are highlighted with a light gray background or indicated by track changes.

Reviewer reports:

Reviewer #1

Reviewer #1: 1. The Line or page number should be added in the revised manuscript, it is hard to point the comment to definite line.

We apologize for the inconvenience. We have now included line and page numbers in this revised version of the manuscript.

2. The methods and parameters of TE analysis should be detailed in the main text or supplementary file, especially for the LAI calculation, the LAI output by our pipeline is 11.47 and the pipeline was built according to default parameters of LTR_retiever (https://github.com/oushujun/LTR_retriever).

Our transposable element (TE) pipeline is based on the EDTA version with AnnoSINE and MGEScan-non-LTR software for SINE and LINE detections, respectively. It also uses TEsorter for element validation and classification. This pipeline is described in Supplementary Information 1. We have also provided instructions for running this pipeline and replicating our results in our GitHub repository (https://github.com/amvarani/Plant_Annotation_TEs).
This pipeline employs adjusted parameters designed to identify as many LTR elements as possible, including non-autonomous elements such as LARDs, TRIMs, and others considered important evolutionary features in plant genomes.
One initial consideration is that the LTR motifs may become degenerated through mutations depending on the age of the element. Therefore, we used two distinct approaches with ltrharvest:
• In the first approach, we used the common TG...CA motif, which is already implemented in EDTA. The command line for Round #1, considering the TGCA motif, was: -minlenltr 100 -maxlenltr 7000 -maxdistltr 20000 -mintsd 4 -maxtsd 6 -motif TGCA -motifmis 1 -similar 85 -vic 10 -seed 20 -seqids yes
• For Round #2, not considering the TGCA motif (non-canonical motifs), the command line was: -minlenltr 100 -maxlenltr 7000 -maxdistltr 20000 -mintsd 4 -maxtsd 6 -similar 85 -vic 10 -seed 20 -seqids yes
For ltr_finder, we accommodated the same parameters: -w 2 -C -D 20000 -d 1000 -L 7000 -l 100 -p 20 -M 0.85

In LTR_retriever, we used the flag -nonTGCA for non-canonical motifs identified by ltrharvest.
Please note that the maximum distance between LTRs was set to 20,000 bp, which is 5 kb longer than the values used in EDTA.
Additionally, our pipeline implements TEsorter to classify all identified LTR elements into Copia and Gypsy superfamilies and lineages, using TEsorter's default parameters with the flags -db rexdb-plant and --hmm-database rexdb-plant, and applying the 80-80-80 (identity-coverage-length) rule.
Using this approach, we are able to determine the completeness and intactness of LTR elements, to classify them into lineages, and to identify potential non-autonomous elements such as TR_GAG, BARE-2, LARD, and TRIMs according to PlantLTRdb (https://doi.org/10.3389/fpls.2023.1134627, please see figure 1).
Regarding the LAI calculation, the reviewer is correct about their verification of the LAI score using default parameters. However, the differences noted here may be related to modifications implemented in our pipeline. According to the LAI manuscript (Ou et al., 2018), long internal regions of LTR elements and high-quality assemblies may positively impact the LAI score. Therefore, we believe that the parameter of the maximum distance between LTRs, set here to 20,000 (with 15,000 being the default), combined with the higher genome coverage and quality of the assembly (please see the responses to Reviewer #2), could be the reason for our slightly higher LAI score reported in the manuscript.
For example, we have calculated the internal regions of LTR elements using our pipeline and identified at least 20 intact LTR elements with long internal regions spanning more than 15,000 bp. Collectively, these elements account for up to 12,850 occurrences, spanning 14,686,032 bp, and representing 3.46% of the T. grandiflorum genome. Thus, a considerable fraction of the genome is now considered for LAI calculation, which may impact its results.
In fact, an LAI score between 10 and 20 is sufficient to consider a genome of reference quality, and T. grandiflorum exhibits an LAI pattern between 10 and 20, thus it can be considered well-assembled and of high quality.
We have explained in Supplementary Information 1 the parameters used in our pipeline and their potential impact on the LAI score.

3. What was the mutation rate (r) used for TE insert time calculation? If the insertion time were from the original files of EDTA, please notice that the default r is 1.3e-8 of grass family once --u was not set with promoting EDTA, that should be converted with the correct r value.

To the best of our knowledge, there is no average substitution rate available for the Theobroma genus, and the generic molecular clock is not suitable for this kind of calculation. Therefore, we decided to use the default value for the mutation rate (1.3e-8), which is commonly used for grasses and assumes at least a 2-fold higher mutation rate in TEs than in coding regions. We understand that our genus is quite distant from grasses.
Upon reviewing the literature, we found a recent study describing a plant LTR database, PlantLTRdb (see Mokthar et al., 2023; https://doi.org/10.3389/fpls.2023.1134627). Since an average substitution rate is not available for many plants, Mokthar et al. (2023) implemented a substitution rate of 1.5e-8 for plants other than grasses. This substitution rate of 1.5e-8 was based on previous studies, including LTR dating in Arabidopsis, flax (Malpighiales), and Eucalyptus (for more details, please see the “2.2 LTR-RT identification and classification” section of PlantLTRdb).
Although the difference between these rates is minimal (2e-9), we recalculated the age of the T. grandiflorum LTR elements using the 1.5e-8 rate for verification and validation purposes. We observed a reduction of approximately 15%, indicating that the age estimated using this rate are slightly younger. This minor adjustment does not significantly affect the overall conclusions of our results.
Given the lack of a specific substitution rate for Theobroma, we have chosen to retain the default mutation rate used in EDTA (1.3e-8), considering this an area for future research.
We have detailed this approach in the supplementary information 1 and included a discussion on this topic in the relevant section of our results. We have added the statement: “the age of LTR insertions was estimated using the default rate of 1.3×10−8 8 substitutions per site per year, making this calculation an approximate estimation.”

4. Generally, the Gypsy content was usually more than Copia content in plant genome, please check it. If it were correct, please infer the reason.

Previous analyses on Theobroma cacao have observed similar trends, as highlighted in the referenced study (see Table S15 in doi: 10.1186/gb-2013-14-6-r53). However, it is important to acknowledge that these results were generated over a decade ago, during which time both the transposable element (TE) pipelines and classification systems have significantly evolved. Nowadays, plant TEs can be classified with greater precision, encompassing evolutionary lineages and non-autonomous LTR elements such as SoloLTRs, LARD, TRIM, TR-GAG, and BARE-2, among others.

The goal of our TE pipeline is to comprehensively label all identified elements, thereby enhancing our understanding of the real diversity and impact of these elements within a specific plant genome. Utilizing the constraints of our pipeline based on the TEsorter 80-80-80 rule, we discovered that many LTR elements previously classified as Copia or Gypsy by tools like LTR_retriever and RepeatModeler are in fact non-autonomous elements, like LARD, TRIM, BARE2, and TR_GAG.

Our findings indicate that non-autonomous LTR elements constitute 12.37% of the T. grandiflorum genome, while Copia and Gypsy elements represent 18.31% and 13.18%, respectively. Notably, nearly all TR_GAG elements exhibit Gypsy-like GAG characteristics, accounting for almost 4% of the genome. Conversely, many TRIM and LARD elements were initially classified as Gypsy or Copia by LTR_retriever. However, upon closer inspection and using TEsorter, we did not identify complete domains but rather relics resembling mostly Gypsy, and in minor number Copia elements. Consequently, we have opted to reclassify these elements as TRIM or LARDs, which we believe is more appropriate for comparative and evolutionary studies.

In summary, our findings suggest that although the Gypsy content in T. grandiflorum appears to be younger than Copia, these elements seem to be decaying at a faster rate, resulting in the generation of non-autonomous representatives. Moreover, and equally importantly, we have tested our pipeline with the same parameters and settings on several other plant genomes. We consistently observed a trend where the Gypsy content is usually higher than that of Copia. This finding may also supports the above mentioned hypothesis.
However, we acknowledge that further analyses, and a new and dedicated study is required to more appropriately substantiate this hypothesis.
Therefore, we have decided not to discuss this result in the manuscript, as it necessitates further and more in-depth analyses that are beyond the scope of this study.

5. All results of GO enrichment were better enriched with KEGG.

We agree that KEGG enrichment analyses are indeed a powerful tool for investigating genomic evolutionary trends. However, while we acknowledge the potential value of KEGG enrichment to our analysis, we have opted not to include it at this stage for several practical reasons. Firstly, incorporating KEGG enrichment analyses would require the generation of new tables, figures, and additional analyses, which would significantly expand the size of our manuscript, already considerable in length.
Additionally, it is crucial to consider that our manuscript is designed to provide a concise and focused narrative on Gene Ontology, exclusive gene families and positive gene selection. Incorporating KEGG enrichment analyses, while potentially enriching, could lead to a more extensive discussion, thereby significantly increasing the length of the manuscript. Our aim is to maintain a streamlined presentation of our findings, ensuring clarity and accessibility for the readers.
Therefore, while we appreciate the valuable suggestion from the reviewer, we have chosen to maintain the current scope of the manuscript, focusing on the analyses that have already been performed and discussed.
We hope that Reviewer #1 will understand our arguments and perspective on this matter.

6. The results about enrichment were written hastily, lots of GO function or GO numbers were just listed, the details should be abundant. Cite the Figures or tables or references in these sections.

Reviewer #1 is correct regarding the presentation of the enrichment results. We acknowledge that the initial version may have appeared hastily composed, with numerous Gene Ontology (GO) functions and

numbers merely listed without sufficient detail. Taking this constructive feedback into account, we have thoroughly revised this section to provide a more comprehensive and detailed explanation of the enrichment results. We have also made sure to cite the relevant figures, tables, and references to offer a clearer and more informative context. We appreciate this valuable input and believe that these adjustments have significantly improved the clarity and depth of our manuscript.

7. In Figure 1C, the Ks distribution needs correction, the authors can refer the polyploidization of durian genome published in Plant physiology in 2019.

We believe the observed differences in the Ks peaks between our study and that of Wang et al. (Durian) can be attributed to distinct methodological approaches.
Our methodology follows the Qiao et al. approach (doi: 10.1186/s13059-019-1650-2), which essentially employs MAFFT with the L-INS-i option for sequence alignment and utilizes PAL2NAL to convert protein alignments into codon alignments. Subsequently, the Ka/Ks ratio is calculated using the γ-MYN method, which incorporates the Tamura–Nei model. Conversely, the Durian study employs the Nei-Gojobori method with additional corrections for their calculations.
Furthermore, our approach to inferring gene colinearity differs as well. We adhere to the Qiao approach, integrating it with the DupGen_finder pipeline, whereas the Durian study utilizes the ColinearScan tool for their analyses.
More importantly, our Ka/Ks calculation for T. cacao aligns precisely with that inferred by Qiao et al., as detailed in Additional File 4 of their paper, and corroborating the core eudicot γ whole-genome triplication event. To prevent any misinterpretation of the results we have presented, we have explicitly described the approach used in our work in the Material and Methods section. We have also added the Qiao et al. reference in the appropriate section of the results.

8. In Figure 2C, why some orders of TE loss the SD?

Indeed, for some elements, calculating the standard deviation was not feasible due to a low number of copies with complete LTRs, thereby lacking sufficient data for an accurate SD age estimation.

9. In Figure 3A, T. grandiflorum and T. cacao present highly syntenic at gene level, the software of Liftoff might detect extra genes to T. grandiflorum genome based on the T. cacao genome. This is just a suggestion.

This suggestion was valuable, and we indeed utilized Liftoff and BLAST in an attempt to identify any missing genes. However, we did not discover any additional missing gene in the T. grandiflorum genome.

10. In Figure 5A, there were 282 special genes in T. grandiflorum, please enrichment them with GO and KEGG.

Indeed, there are 282 gene families. We conducted several Gene Ontology (GO) enrichment analyses across all combinations presented in Figure 5A and did not find any statistically significant enrichment. This information has been clearly stated in the revised version of the manuscript.
Moreover, the 282 gene families list can be easily retrieved in the Table S10, and further explored in terms of important and relevant genes related to agronomical traits in Figure 5B (please see comment 11 below).

11. Figure 5B and D were from the GO enrichment, the GO numbers should be added around annotation or list them in the supplementary files.

Actually, Figure 5B and 5D are not the result of Gene Ontology (GO) enrichment analyses. Figure 5B illustrates exclusive gene families that are associated with genes important for agronomical traits, while Figure 5D showcases gene families that have expanded or contracted, also in relation to agronomical traits. These figures are accompanied by their respective supplementary Tables, specifically Tables S10, S11, and S12. To further clarify the significance of these results, we have incorporated an additional paragraph in the corresponding section of the manuscript.

12. In Figure 5C, the confidence interval of divergence time should be added.

In Figure 5C, we utilized the TimeTree5 database (doi: 10.1093/molbev/msac174) to estimate the divergence time among species. According to the TimeTree5 Frequently Asked Questions:

“Q: How did you derive confidence intervals for molecular time estimates?

A: Our primary aim is to inform users about differences that exist among studies in estimated time through the presentation of "confidence intervals." We based our confidence interval on the Empirical Rule (95%) in statistics, which states that, assuming a normal distribution, approximately 95% of the times reported across studies will fall within two standard deviations of the mean time. That is, we used the 95% Empirical Rule for a population of studies, which has an interpretation similar to confidence intervals and hypothesis testing. Intuitively, we expect that the among-study variance, obtained based on study node times as single data points, captures a variety of lower-level errors including differences in calibrations and sampling of genes and taxa. We present this confidence interval for nodes where times are available from five or more studies - otherwise a min-max range of time estimates is given (or no range if only a single estimate is available). We provide The Empirical Rule confidence interval as a quick guide to the amount of variation among studies, and we strongly recommended that researchers review individual studies and their methodologies before using any time estimates in downstream research.”

Therefore, the confidence interval was added to Figure 5C whenever this information was available. Additionally, we have incorporated details regarding this in both the Material and Methods section and the Figure legend.

13.In the data availability, the weblink is not for everyone, GigaDB will record your data, so the unopened weblink might not necessary. [EDITOR's note: Indeed, our data curators can host your data in GigaDB - we can sort this out after submission of the revised version]

Ok, we will await further instructions before proceeding with any changes.

14. In the MS, disease resistance were mentioned repeatedly, the GO enrichment has been provided some evidence, it will be better to perform the KEGG analysis with the special genes and expanded or contracted genes to verify, especially stat the changes in the ko04626.

We have recently sequenced the chromosome-scale genome of another T. grandiflorum genotype, which, in contrast to the genome discussed in this manuscript, exhibits resistance to witches' broom disease (Clone C174). Consequently, we are currently undertaking comprehensive analyses, including those related to plant-pathogen interaction pathways, resistant genes, and transcriptome, for inclusion in another manuscript that is under preparation.

15. The language must be improved and modified by native academic English speaker.

In this revised version, the manuscript has been reviewed by a native English speaker.

We would like to thank Reviewer #1 for their time and dedication in reviewing our manuscript.

Reviewer #2

Reviewer #2: Rafael et al. contributed their study, "Genomic decoding of Theobroma grandiflorum (cupuassu) at chromosomal scale: Evolutionary insights for horticultural innovation". In this study, high-quality genome assembly for an important plant was generated and the authors further investigated genome characterization, genome evolution, gene families etc. The data quality is high, though some points need to be clarified. And the reported data and investigations could provide valuable inference for

following studies.

This paper is generally well-prepared.

We would like to thank Reviewer #2 for their time and dedication in reviewing our manuscript.

Major comments:
1. Quality control of genome assembly. The quality of genome assembly could be better evaluated with more stringent parameters. On assembly quality control, I will recommend to always follow criteria established in Earth Biogenome Project (Report on Assembly Standards, https://www.earthbiogenome.org/assembly-standards). Please evaluate the present assemblies with the criteria from EBP project, I think, on at least some if not all the items. At least, I think Merqury results would be very informative.

Merqury: reference-free quality, completeness, and phasing assessment for genome assemblies https://genomebiology.biomedcentral.com/articles/10.1186/s13059-020-02134-9

We appreciate the reviewer comment. We have incorporated the Merqury and Inspector analysis reports (https://doi.org/10.1186/s13059-021-02527-4) in the new version of Table 1, and reorganized the first section of the results, describing the genome assembly and quality control in more detail as suggested. Additionally, we have added a new Table 1 and provided the complete output of the BUSCO scores in Supplementary Table S2.

2. Gaps in each pseudo-chromosome. Not clear if gaps are still remained, or the genome is of gap-free?

There are only three remaining gaps, each spanning approximately 100 bp. These gaps are located near the telomeric regions of chromosomes 8 and 6, and around the centromeric regions of chromosome 5. We have now stated this information in the results section and provided a new supplementary table (Table S3) to detail these findings.

3. Centromere region. How centromeres were identified? Centromeres were shown, but no description on how you did identify them. Given the high quality of genome assembly, it would be very interesting to incorporate the investigation into distribution of centromeres. A pipeline (https://github.com/ShuaiNIEgithub/Centromics (identifying centromere with multi-oimcs data, such as repeat profiling, and Hi-C chromatin contact) is helpful, and it was generally described at https://academic.oup.com/hr/article/10/1/uhac241/6775201?login=true) has already been prepared and widely applied in data analyses in some just published T2T assemblies.

Thanks for the valuable suggestion. Initially, we identified centromeres using the Tandem Repeat Finder tool, as described in our GitHub pipeline (https://github.com/amvarani/Plant_Annotation_TEs). However, we acknowledge that our previous method was simplistic and based on an outdated approach (refer to DOI: 10.1186/gb-2013-14-1-r10). Subsequently, we adopted the recommended pipeline and identified a recent publication detailing a new pipeline, named quarTeT (Lin et al., 2023), specifically designed for identifying telomeric and centromeric repeats. Employing both pipelines enabled us to predict the telomeric and centromeric regions of the genome effectively. In summary, both pipelines yielded comparable results, which we combined to enhance the robustness of our predictions for telomeric and centromeric regions. The centromeric repeats we identified were present in all chromosomes and aligned with findings from previous cytogenetic studies (e.g. https://doi.org/10.1590/S1415-47572009005000103). We incorporated some minor adjustments to Figure 3 and included the necessary details in the legend of Figure 3, as well as in the Results and Material and Methods sections. Additionally, we have added two new supplementary tables (Table S4 and S5) that provide a summary of the identified telomeres and centromeres, now available for reference.
