# Supplementary material for: Genomic decoding of Theobroma grandiflorum (cupuassu) at chromosomal scale: evolutionary insights for horticultural innovation
Source: Gigascience. 2024 Jun 5;13:giae027. doi: 10.1093/gigascience/giae027 (PMC11152179; doi:10.1093/gigascience/giae027)
Supplement: giae027_Response_to_Reviewer_Comments_Revision_1 [file giae027_response_to_reviewer_comments_revision_1.pdf]

Author's Response To Reviewer Comments

1) The reviewer has a few minor follow-up points and wording suggestions. Please have a look over the list below and revise the paper where applicable. (Note: the follow-up comments are only suggestions and not mandatory to address, especially if they are about word choice/style etc.).

All comments were accepted

2) If you have a photograph of the sequenced species, please feel free to include an image of the plant as a Figure in the paper. (Note: you need permission to publish the photo under the "creative commons attribution" [cc-by] licence.

We have added a new Figure 1, showing the species sequenced. We have the permissions to publish these photos

3) Please add ORCIDs for all authors on the title page, where available. We have the following on our files: ORCID iDs: Rafael Moysés Alves [0000-0002-9826-4690]; Vinicius A C de Abreu [0000-0002-4243-2421]; Rafaely Pantoja Oliveira [0000-0003-4907-3289]; João Victor dos Anjos Almeida [0000-0003-1255-5831]; Mauro de Medeiros de Oliveira [0000-0002-2048-6664]; Saura R Silva [0000-0002-6333-5268]; Alexandre R Paschoal [0000-0002-8887-0582]; Sintia S de Almeida [0000-0003-0270-9059]; Pedro A F de Souza [0009-0002-9870-0023]; Jesus A Ferro [0000-0002-3966-1303]; Vitor F O Miranda [0000-0003-0574-9865]; Douglas S Domingues [0000-0002-1290-0853]; Antonio Figueira [0000-0001-8641-2556]; Alessandro M Varani [0000-0002-8876-3269]

OK

4) Please add the NCBI taxonID for the species when you first mention the species in the methods section. I think the ID is NCBI:txid108881, but please double check.

OK

5) At GigaScience, we treat online sources of code and data as citable items. Please move in-text URLs for code/data sources to the bibliography as separate items and cite them by reference number in the text. e.g. JABU, line 163, and all github repos for code mentioned should have their own, numbered citation in the bibliography, including the github URL. Please refer to our citation guidelines in our Information for Authors on the GigaScience homepage.

Done

6) Our data curators will now assess the submission's data and, if applicable, they will contact you shortly to prepare a supporting data set for our repository GigaDB (which should then be cited from the Data Availability section).

OK

7) At this stage, you can remove all highlighting / tracking of changes that was made for the purpose of peer review.

OK

Reviewer report:
Reviewer #1: 1. Line 52, we present a telomere-to telomere assembly of cupuassu genome and disclosed its features of ……

Here, we present the sequenced telomere-to-telomere genome of cupuassu, disclosing its genomic structure, evolutionary features, and phylogenetic relationships within the Malvaceae family.

2. Line 57, are is were, burst and expansion were repetitive, please retained only one of them.

OK

3. Line 73-82, key point should be complied with single format and style, such as same tense and brief sentence pattern. Here is some suggestion:

The chromosome-scale of Theobroma grandiflorum was generated and revealed a 65% synteny with T. cacao.
LTR retrotransposon expansion was a pivotal factor for post-divergence genomic evolution between Theobroma species.
Comparative genomics provided evolutionary insights of the genes associated with key agronomic traits.
Positive selection pressure of retained duplicated genes implicated in adaptive functions and fruit-seed trait diversity.

OK

4. Line 97, first word "for" might be "of".

OK

5. Line 97-100, please consider the following:
We conducted a comprehensive sequencing of the cupuassu genome employing technologies of PacBio HiFi, genome-wide chromatin interaction analysis via Hi-C alongside Illumina sequencing.

OK

6. Line 65, "Sequencing" could be deleted.

OK

7. Line 66, "breeding improvement and conservation biology", this is just a suggestion.

OK

8. Cupuassu genome is suggested to add in the key words.

OK

9. Line 101-103 was dissected with its context. The function of the iso-seq and RNA-seq with short reads should be linked with assist predication and annotation of genes.

OK

10. Line 105-106, is the similarity based on gene synteny level or genomic sequence level?

OK

11. Line 118, "Both" could be replaced with "Both of them".

OK

12. Line 147, the word "sequencing" could be deleted.

OK

13. Line 173, GenomeScope 2.0 and KMC were employed to evaluate the ploidy and size of genome.

OK

14. Line 174, Total RNA was extracted and purified using…..

OK

15. IsoSeq could be replaced with Iso-seq in the total text.

OK

16. Line 250-252, the software of OrthoFinder2 is before Ortho Venn3.

Not changed

17. Line 255, analyzed species could be replaced with species-selected.

OK

18. Based on the pipeline in methods, the section of TE analysis is before the section of gene analysis or enrichment.

That is correct

19. Line 337, molecular dating means molecular clock?

FIXED

20. Line 355-356, it is recommended to revise as "possibly impacting gene expression patterns and their regulator networks".

OK

21. Line 418, chalcone is one of types of flavonoids, so delete it will be better.

OK

22. Line 420, the word "seeds" might be deleted. These compound not only impact the aromas of seeds, but also might be contributed to the flavor of flower, fruit, even to the taste.

OK

23. Line 430, delete the In summary,

OK

24. Line 438, "Spatial arrangement and" was recommended to delete. The duplicate types of plant genes were analyzed in whole genome were just based on their similarity of amino acids or linearity, there were no evidence to confirm the spatial arrangement in this analysis.

OK

25. Line 445-446, the percentages of each duplicate types from T. grandiflorum should be described in the main text.
This information is provided in details in the supplementary material

26. The word "Most of" refers to the statistics were recommended to replace with numbers and their percentages in the whole text.

Not really

27. Line 564-616, there are lots of citation and derived vast words of "may" or "might", please confirm the results in the citation is obtained from experiment or just speculation, there are recommended to replace with "indicated"、"demonstrated" if they were from experiment and the others could be retained or replace with "suggested".

OK

28. Line 619-625, this paragraph might not necessary.

We decide to maintain this paragraph

29. Line 627, enhancing "its" genetics resources.

OK

30. There were lots of "we" words in the main text, it will be more objective to describe the results and discussion with passive voice.

OK
