# Supplementary material for: Genomic decoding of Theobroma grandiflorum (cupuassu) at chromosomal scale: evolutionary insights for horticultural innovation
Source: Gigascience. 2024 Jun 5;13:giae027. doi: 10.1093/gigascience/giae027 (PMC11152179; doi:10.1093/gigascience/giae027)
Supplement: giae027_Reviewer_1_Report_Original_Submission — Xupo Ding -- 2/21/2024 [file giae027_reviewer_1_report_original_submission.pdf]

Reviewer Report

Title: Genomic decoding of Theobroma grandiflorum (cupuassu) at chromosomal scale: Evolutionary insights for horticultural innovation

Version: Original Submission **Date:** 2/21/2024

Reviewer name: Xupo Ding

Reviewer Comments to Author:

1. The Line or page number should be added in the revised manuscript, it is hard to point the comment to definite line.2. The methods and parameters of TE analysis should be detailed in the main text or supplementary file, especially for the LAI calculation, the LAI output by our pipeline is 11.47 and the pipeline was built according to default parameters of LTR_retiever (https://github.com/oushujun/LTR_retriever).3. What was the mutation rate (r) used for TE insert time calculation? If the insertion time were from the original files of EDTA, please notice that the default r is 1.3e-8 of grass family once --u was not set with promoting EDTA, that should be converted with the correct r value.4. Generally, the Gypsy content was usually more than Copia content in plant genome, please check it. If it were correct, please infer the reason.5. All results of GO enrichment were better enriched with KEGG.6. The results about enrichment were wrote hastily, lots of GO function or GO numbers were just list, the details should be abundant. Cite the Figures or tables or references in these sections.7. In Figure 1C, the Ks distribution need corrective, the authors can refer the polyploidization of durian genome published on Plant physiology in 2019.8. In Figure 2C, why some orders of TE loss the SD?9. In Figure 3A, T. grandiflorum and T. cacao present highly syntenic at gene level, the software of Liftoff might detect extra genes to T. grandiflorum genome based on the T. cacao genome. This is just a suggestion.10. In Figure 5A, there were 282 special genes in T. grandiflorum, please enrichment them with GO and KEGG.11. Figure 5B and D were from the GO enrichment the GO numbers should be added around annotation or list them in the supplementary files.12. In Figure 5C, the confidence interval of divergence time should be added.13. In the data availability, the weblink is not for everyone, GigaDB will record your data, so the unopened weblink might not necessary.14. In the MS, disease resistance were mentioned repeatedly, the GO enrichment has been provided some evidence, it will be better to perform the KEGG analysis with the special genes and expanded or contracted genes to verify, especially stat the changes in the ko04626.15. The language must be improved and modified by naive academic English speaker.

Level of Interest

Please indicate how interesting you found the manuscript: Choose an item.

Quality of Written English

Please indicate the quality of language in the manuscript: Choose an item.

Declaration of Competing Interests

Please complete a declaration of competing interests, considering the following questions:

- Have you in the past five years received reimbursements, fees, funding, or salary from an organisation that may in any way gain or lose financially from the publication of this manuscript, either now or in the future?
- Do you hold any stocks or shares in an organisation that may in any way gain or lose financially from the publication of this manuscript, either now or in the future?
- Do you hold or are you currently applying for any patents relating to the content of the manuscript?
- Have you received reimbursements, fees, funding, or salary from an organization that holds or has applied for patents relating to the content of the manuscript?
- Do you have any other financial competing interests?
- Do you have any non-financial competing interests in relation to this paper?

If you can answer no to all of the above, write 'I declare that I have no competing interests' below. If your reply is yes to any, please give details below.

I declare that I have no competing interests.

I agree to the open peer review policy of the journal. I understand that my name will be included on my report to the authors and, if the manuscript is accepted for publication, my named report including any attachments I upload will be posted on the website along with the authors' responses. I agree for my report to be made available under an Open Access Creative Commons CC-BY license (http://creativecommons.org/licenses/by/4.0/). I understand that any comments which I do not wish to be included in my named report can be included as confidential comments to the editors, which will not be published.

Choose an item.

To further support our reviewers, we have joined with Publons, where you can gain additional credit to further highlight your hard work (see: https://publons.com/journal/530/gigascience). On publication of this paper, your review will be automatically added to Publons, you can then choose whether or not to claim your Publons credit. I understand this statement.

Yes Choose an item.
