# Supplementary material for: Genomic decoding of Theobroma grandiflorum (cupuassu) at chromosomal scale: evolutionary insights for horticultural innovation
Source: Gigascience. 2024 Jun 5;13:giae027. doi: 10.1093/gigascience/giae027 (PMC11152179; doi:10.1093/gigascience/giae027)
Supplement: giae027_Reviewer_1_Report_Revision_1 — Xupo Ding -- 3/29/2024 [file giae027_reviewer_1_report_revision_1.pdf]

Reviewer Report

Title: Genomic decoding of Theobroma grandiflorum (cupuassu) at chromosomal scale: Evolutionary insights for horticultural innovation

Version: Revision 1 **Date:** 3/29/2024

Reviewer name: Xupo Ding

Reviewer Comments to Author:

1. Line 52, we present a telomere-to telomere assembly of cupuassu genome and disclosed its features of ……2. Line 57, are is were, burst and expansion were repetitive, please retained only one of them.3. Line 73-82, key point should be complied with single format and style, such as same tense and brief sentence pattern. Here is some suggestion:The chromosome-scale of Theobroma grandiflorum was generated and revealed a 65% synteny with T. cacao.LTR retrotransposon expansion was a pivotal factor for post-divergence genomic evolution between Theobroma species.Comparative genomics provided evolutionary insights of the genes associated with key agronomic traits.Positive selection pressure of retained duplicated genes implicated in adaptive functions and fruit-seed trait diversity.4. Line 97, first word "for" might be "of".5. Line 97-100, please consider the following:We conducted a comprehensive sequencing of the cupuassu genome employing technologies of PacBio HiFi, genome-wide chromatin interaction analysis via Hi-C alongside Illumina sequencing.6. Line 65, "Sequencing" could be deleted.7. Line 66, "breeding improvement and conservation biology", this is just a suggestion.8. Cupuassu genome is suggested to add in the key words.9. Line 101-103 was dissected with its context. The function of the iso-seq and RNA-seq with short reads should be linked with assist predication and annotation of genes.10. Line 105-106, is the similarity based on gene synteny level or genomic sequence level?11. Line 118, "Both" could be replaced with "Both of them".12. Line 147, the word "sequencing" could be deleted.13. Line 173, GenomeScope 2.0 and KMC were employed to evaluate the ploidy and size of genome.14. Line 174, Total RNA was extracted and purified using…..15. IsoSeq could be replaced with Iso-seq in the total text.16. Line 250-252, the software of OrthoFinder2 is before Ortho Venn3.17. Line 255, analyzed species could be replaced with species-selected.18. Based on the pipeline in methods, the section of TE analysis is before the section of gene analysis or enrichment.19. Line 337, molecular dating means molecular clock?20. Line 355-356, it is recommended to revise as "possibly impacting gene expression patterns and their regulator networks".21. Line 418, chalcone is one of types of flavonoids, so delete it will be better.22. Line 420, the word "seeds" might be deleted. These compound not only impact the aromas of seeds, but also might be contributed to the flavor of flower, fruit, even to the taste.23. Line 430, delete the In summary,24. Line 438, "Spatial arrangement and" was recommended to delete. The duplicate types of plant genes were analyzed in whole genome were just based on their similarity of amino acids or linearity, there were no evidence to confirm the spatial arrangement in this analysis.25. Line 445-446, the percentages of each duplicate types from T. grandiflorum should be described in the main text.26. The word "Most of" refers to the statistics were recommended to replace with numbers and their percentages in the whole text.27. Line 564-616, there are lots of citation and derived vast words of "may" or "might", please confirm the results in the citation is obtained from

experiment or just speculation, there are recommended to replace with "indicated"ã€"demonstrated" if they were from experiment and the others could be retained or replace with "suggested".28. Line 619-625, this paragraph might not necessary.29. Line 627, enhancing "its" genetics resources.30. There were lots of "we" words in the main text, it will be more objective to describe the results and discussion with passive voice.

Level of Interest

Please indicate how interesting you found the manuscript: Choose an item.

Quality of Written English

Please indicate the quality of language in the manuscript: Choose an item.

Declaration of Competing Interests

Please complete a declaration of competing interests, considering the following questions:

- Have you in the past five years received reimbursements, fees, funding, or salary from an organisation that may in any way gain or lose financially from the publication of this manuscript, either now or in the future?
- Do you hold any stocks or shares in an organisation that may in any way gain or lose financially from the publication of this manuscript, either now or in the future?
- Do you hold or are you currently applying for any patents relating to the content of the manuscript?
- Have you received reimbursements, fees, funding, or salary from an organization that holds or has applied for patents relating to the content of the manuscript?
- Do you have any other financial competing interests?
- Do you have any non-financial competing interests in relation to this paper?

If you can answer no to all of the above, write 'I declare that I have no competing interests' below. If your reply is yes to any, please give details below.

I declare that I have no competing interests.

I agree to the open peer review policy of the journal. I understand that my name will be included on my report to the authors and, if the manuscript is accepted for publication, my named report including any attachments I upload will be posted on the website along with the authors' responses. I agree for my report to be made available under an Open Access Creative Commons CC-BY license (http://creativecommons.org/licenses/by/4.0/). I understand that any comments which I do not wish to be included in my named report can be included as confidential comments to the editors, which will not be published.

Choose an item.

To further support our reviewers, we have joined with Publons, where you can gain additional credit to further highlight your hard work (see: https://publons.com/journal/530/gigascience). On publication of this paper, your review will be automatically added to Publons, you can then choose whether or not to claim your Publons credit. I understand this statement.

Yes Choose an item.
