# Supplementary material for: Genomic decoding of Theobroma grandiflorum (cupuassu) at chromosomal scale: evolutionary insights for horticultural innovation
Source: Gigascience. 2024 Jun 5;13:giae027. doi: 10.1093/gigascience/giae027 (PMC11152179; doi:10.1093/gigascience/giae027)
Supplement: giae027_Reviewer_2_Report_Original_Submission — Jian-Feng Mao -- 2/26/2024 [file giae027_reviewer_2_report_original_submission.pdf]

Reviewer Report

Title: Genomic decoding of Theobroma grandiflorum (cupuassu) at chromosomal scale: Evolutionary insights for horticultural innovation

Version: Original Submission **Date:** 2/26/2024

Reviewer name: Jian-Feng Mao

Reviewer Comments to Author:

Rafael et al. contributed their study, "Genomic decoding of Theobroma grandiflorum (cupuassu) at chromosomal scale:Evolutionary insights for horticultural innovation". In this study, high-quality genome assembly for an important plant was generated and the authors further investigated genome characterization, genome evolution, gene families etc. The data quality is high, though some points need to be clarified. And the reported data and investigations could provide valuable inference for following studies.This paper is generally well-prepared.Major comments:1. Quality control of genome assembly. The quality of genome assembly could be better evaluated with more stringent parameters. On assembly quality control, I will recommend to always follow criteria established in Earth Biogenome Project (Report on Assembly Standards, https://www.earthbiogenome.org/assembly-standards). Please evaluate the present assemblies with the criteria from EBP project, I think, on at least some if not all the items. At least, I think Merqury results would be very informative.Merqury: reference-free quality, completeness, and phasing assessment for genome assemblies https://genomebiology.biomedcentral.com/articles/10.1186/s13059-020-02134-92. Gaps in each pseudo-chromosome. Not clear if gaps are still remained, or the genome is of gap-free?3. Centromere region. How centromeres were identified? Centromeres were shown, but no description on how you did identify them. Given the high quality of genome assembly, it would be very interesting to incorporate the investigation into distribution of centromeres. A pipeline (https://github.com/ShuaiNIEgithub/Centromics (identifying centromere with multi-oimcs data, such as repeat profiling, and Hi-C chromatin contact) is helpful, and it was generally described at https://academic.oup.com/hr/article/10/1/uhac241/6775201?login=true) has already been prepared and widely applied in data analyses in some just published T2T assemblies.

Level of Interest

Please indicate how interesting you found the manuscript: Choose an item.

Quality of Written English

Please indicate the quality of language in the manuscript: Choose an item.

Declaration of Competing Interests

Please complete a declaration of competing interests, considering the following questions:

- Have you in the past five years received reimbursements, fees, funding, or salary from an organisation that may in any way gain or lose financially from the publication of this manuscript, either now or in the future?
- Do you hold any stocks or shares in an organisation that may in any way gain or lose financially from the publication of this manuscript, either now or in the future?
- Do you hold or are you currently applying for any patents relating to the content of the manuscript?
- Have you received reimbursements, fees, funding, or salary from an organization that holds or has applied for patents relating to the content of the manuscript?
- Do you have any other financial competing interests?
- Do you have any non-financial competing interests in relation to this paper?

If you can answer no to all of the above, write 'I declare that I have no competing interests' below. If your reply is yes to any, please give details below.

none.

I agree to the open peer review policy of the journal. I understand that my name will be included on my report to the authors and, if the manuscript is accepted for publication, my named report including any attachments I upload will be posted on the website along with the authors' responses. I agree for my report to be made available under an Open Access Creative Commons CC-BY license (http://creativecommons.org/licenses/by/4.0/). I understand that any comments which I do not wish to be included in my named report can be included as confidential comments to the editors, which will not be published.

Choose an item.

To further support our reviewers, we have joined with Publons, where you can gain additional credit to further highlight your hard work (see: https://publons.com/journal/530/gigascience). On publication of this paper, your review will be automatically added to Publons, you can then choose whether or not to claim your Publons credit. I understand this statement.

Yes Choose an item.
